# MBASED: allele-specific expression detection in cancer tissues and cell lines

**DOI:** 10.1186/s13059-014-0405-3

**Published:** 2014-08-07

**Authors:** Oleg Mayba, Houston N Gilbert, Jinfeng Liu, Peter M Haverty, Suchit Jhunjhunwala, Zhaoshi Jiang, Colin Watanabe, Zemin Zhang

**Affiliations:** Department of Bioinformatics and Computational Biology, Genentech Inc, South San Francisco, CA 94080 USA; Department of Biostatistics, Genentech Inc, South San Francisco, CA 94080 USA

## Abstract

Allele-specific gene expression, ASE, is an important aspect of gene regulation. We developed a novel method MBASED, meta-analysis based allele-specific expression detection for ASE detection using RNA-seq data that aggregates information across multiple single nucleotide variation loci to obtain a gene-level measure of ASE, even when prior phasing information is unavailable. MBASED is capable of one-sample and two-sample analyses and performs well in simulations. We applied MBASED to a panel of cancer cell lines and paired tumor-normal tissue samples, and observed extensive ASE in cancer, but not normal, samples, mainly driven by genomic copy number alterations.

**Electronic supplementary material:**

The online version of this article (doi:10.1186/s13059-014-0405-3) contains supplementary material, which is available to authorized users.

## Background

Transcriptional activity at the different alleles of a gene in a non-haploid genome can differ considerably. Both genetic and epigenetic determinants govern this allele-specific expression (ASE) [1] and impairment of this highly regulated process can lead to disease [2]. To understand the biological role of ASE and its underlying mechanisms, a comprehensive identification of ASE events is required. Recent advances in sequencing technology enable investigation of entire genomes at increasingly fine resolution. Whole exome DNA sequencing (WES) or whole genome DNA sequencing (WGS) allows identification of single nucleotide mutations or polymorphisms in all exonic regions or the entire human genome, respectively, while messenger RNA sequencing (RNA-Seq) enables quantitative analysis of gene expression. The expression state of the heterozygous loci detected in WES or WGS assays can be investigated in a matched RNA-Seq sample from the same individual, leading to a detailed map of the ASE activity. This approach allows the investigator to uncover the instances of complete or near allele silencing, which would be impossible using only RNA-Seq data.

Next-generation sequencing of short reads is prone to technical biases, for example, over- or under-representation of certain sequence motifs or inhomogeneous mapping, which must be overcome for effective ASE detection [3-5]. In addition, data from multiple heterozygous single nucleotide variants (SNVs) in the same gene must be integrated, and the large number of tested genes requires appropriate statistical treatment of the multiplicity of tested hypotheses. Despite these obstacles, next-generation sequencing technology has been recently used to identify putative sites of ASE within and between samples [4,6-14]. Previous work using short reads to detect ASE focused either on model organisms [11,13] or on normal human tissues or cell lines [4,10,12], although limited studies have explored the ASE landscape in cancer [15,16]. Further, there is currently no standard and robust way to aggregate information across SNVs into a single measure of ASE for an entire transcript isoform or gene. Most published studies either tested ASE at the SNV-level, sometimes requiring agreement across SNVs within a gene [3,6,7,10,12,17,18], or used available phasing information to sum reads across SNVs [4]. A recent study [13] incorporated phased SNV-level information into a gene-level statistical model, allowing for extra variability due to alternative splicing effects on allelic ratios at individual SNVs. However, with the exception of limited samples such as those from the HapMap Project [19], most specimens do not have SNV phasing information. In some cases, population genetics-based approaches and existing databases can be used to phase common single nucleotide polymorphisms (SNPs) [20]. However, the ability to phase common SNPs into individual haplotypes, whether based on previous knowledge or a statistical method, does not apply to somatic mutations in cancer. This makes it challenging to assign the ASE status to the mutant allele and reduces the ability to study the ASE of mutation-carrying genes.

To overcome these difficulties, we developed a novel ASE detection method, called MBASED. MBASED assesses ASE by combining information across individual heterozygous SNVs within a gene without requiring *a priori* knowledge of haplotype phasing; therefore, it can be applied to a wide array of existing RNA-Seq data sets, most of which do not have phasing information available. When phasing information is present, MBASED takes advantage of it to increase the power of ASE detection. In practice, even with modest sequencing depths, a large number of genes show more than one detectable heterozygous exonic SNV in RNA-Seq data, highlighting the importance of having a framework for aggregating expression information across individual loci.

To robustly estimate gene-level ASE from SNV-level RNA-Seq read counts, MBASED employs a meta-analytic approach [21], used originally to combine information from several studies into a global effect estimate. Our approach can be used in both one-sample and two-sample analyses, making MBASED a versatile tool for investigating allele-specific expression, both within an individual sample and in the context of differential ASE.

We applied MBASED to a panel of human lung cancer cell lines and paired tumor-normal lung and liver tissue samples. None of our samples had haplotype phasing information available, exemplifying a typical situation in gene expression studies. Our goal was to investigate the landscape of ASE in cancer and to identify potential instances of ASE contributing to cancer phenotypes. Previous studies of ASE in cancer were limited by sample size [15] (three paired tumor-normal samples) or concentrated on detecting monoallelic expression in the context of loss of heterozygosity events [16]. In this study we present a general view of ASE, monoallelic or otherwise, in a panel of 25 cancer samples across 2 tissue types, including direct tumor/normal comparisons. We observed high rates of ASE (9 to 26%) in tumor tissue samples relative to normal tissue samples (0.5 to 2%), as well as variable ASE rates in cancer cell lines (1 to 31%). We found the observed elevated ASE rates in cancer samples to be mainly driven by underlying changes in genomic copy number and allelic composition. Numerous instances of genes with recurrent ASE in cancer were attributed to recurrent genomic alterations involving known cancer genes, for example, *TP53* and *KRAS*. We found a number of mutations with known or suspected roles in cancer, including L858R mutation in *EGFR* and several G12A and G12C mutations in *KRAS*, to exhibit overexpression of the mutant allele, highlighting potential ASE contribution to cancer phenotypes. Joint analysis of tumors and matched normal samples did not reveal any instances of loss of imprinting, although several instances of loss of ASE in tumor were observed, including a switch of the overexpressed allele in the mono-allelically expressed pro-apoptotic factor *BCL2L10*. Our comprehensive analysis revealed a rich landscape of ASE in cancer and highlighted the flexibility and usefulness of our proposed method MBASED for ASE detection.

## Results and discussion

### MBASED: meta-analysis based allele-specific expression detection

First, we give an overview of our method, MBASED, with detailed descriptions provided in Materials and methods and in Supplementary methods in Additional file 1. Given RNA read counts supporting reference and alternative alleles at individual SNVs within a unit of expression, MBASED provides an estimate of ASE and a corresponding *P*-value. A unit of expression can be a gene, a transcript isoform, an exon, or an individual SNV: MBASED is agnostic with respect to the nature of the unit provided by the user. In this work, we choose the gene as a unit of ASE, which we define as the union of all exons forming individual transcript isoforms.

**Figure 1:**
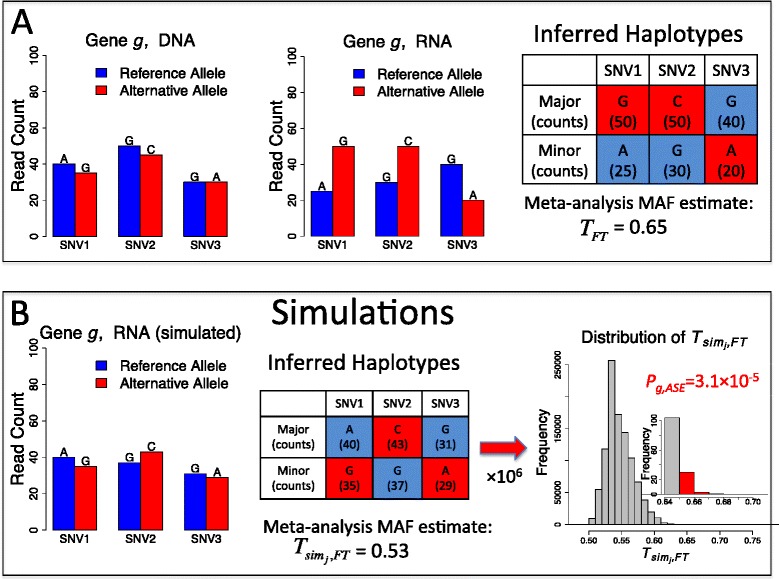
**Overview of MBASED algorithm (one-sample analysis).** The two-sample approach is similar and is described in the text. **(A)** When true haplotypes are unknown, MBASED pseudo-phases SNVs within a gene by creating a major haplotype out of the alleles with larger RNA read counts at each SNV. A meta-analytic approach is then used to aggregate ASE information across individual SNVs to produce a meta-analysis estimate of major haplotype frequency (MAF), *T*_*FT*_. **(B)** Keeping total read counts at each SNV constant, we simulate reference allele counts from a null distribution with an underlying haplotype frequency ratio of 1:1, and then pseudo-phase the alleles into haplotypes based on simulated read counts. Repeating this process 10^6^ times we obtain an estimate of null distribution of *T*_*FT*_ and assign a final ASE *P*-value, *p*_*g*,*ASE*_, to gene *g* as the observed fraction of simulated estimates that are as extreme as or more extreme than *T*_*FT*_.

For a given gene, MBASED provides a framework for aggregation of SNV-level information into a single measure of ASE. The meta-analytic approach adopted by MBASED relies on specification of gene haplotypes, which may be unknown for many data sets. In one-sample ASE analysis, when true haplotypes are unknown, MBASED uses RNA read counts at individual SNVs within a gene to phase SNVs into two haplotypes. We adopt a pseudo-phasing approach that assigns an allele with a larger read count at each SNV to the ‘major’ haplotype, with the implicit assumption that ASE is consistent in one direction along the length of the gene. This procedure is not intended to faithfully reconstruct the true underlying haplotypes in all cases, but we expect it to do so for genes showing sufficiently strong ASE. We quantify the allelic imbalance within a sample as the major allele (haplotype) frequency (MAF) of the gene. The ASE detection then becomes a problem of identifying genes with MAF >0.5. Phased counts from the ‘major’ haplotype are transformed into normally distributed scores, and scores from individual SNVs are combined into a single gene-level score using a meta-analytic approach. This score is then used to obtain an estimate of underlying allelic imbalance. The meta-analytic statistical inference requires the correct specification of gene haplotypes in order to assign proper statistical significance to the observed ASE. Consequently, the pseudo-phasing procedure employed by MBASED in cases of uknown true haplotypes leads to anti-conservative nominal *P*-values (Materials and methods). We address this problem by employing internal simulations to adjust the reported significance levels. For genes showing strong ASE, we expect our phasing procedure to result in an accurate estimate of MAF, while internal simulations will eliminate most of the allelically balanced genes that may exhibit strong nominal significance due to pseudo-phasing. The basic principles of MBASED in absence of phasing information are illustrated in Figure 1.

In two-sample ASE analysis, the goal is to detect differential allelic imbalance between paired samples from the same individual. MBASED treats this problem in an asymmetric way, by designating one of the two samples as the sample of interest, for example, tumor sample in a tumor versus normal comparison. If true haplotypes are unknown, then for any gene that exhibits tumor-specific ASE, only the tumor read counts are informative for separating haplotypes into ‘major’ and ‘minor’. In such cases, MBASED phases alleles at individual SNVs into two haplotypes based exclusively on the sample of interest (tumor, in this case). If normal-specific ASE is under study, for example, when investigating loss of imprinting, then the normal sample can be designated as the sample of interest. Differences between ‘major’ allele frequencies at individual SNVs in the two samples are used as measures of between-sample ASE. SNV-level scores are combined into a gene-level score using meta-analysis, analogous to the one-sample approach. This composite score provides an estimate of gene-level MAF difference between the samples. As in our one-sample approach, internal simulations are used to assign statistical significance to the observed allelic imbalance, in cases of uknown true haplotypes.

We adopt the approach of DerSimonian and Laird [21] in establishing a meta-analysis framework for combining information across SNVs. This approach views the true unobserved treatment effect (in our case, ASE) at each observational unit of a common phenomenon (SNVs of a gene) as a random variable with a common mean. The estimate of that mean is obtained by combining information across individual units and represents a measure of the global effect (gene-level ASE). Within this framework, MBASED also reports the *P*-value corresponding to the constancy of the treatment effect statistic, *Q*, for multi-SNV genes in both one-sample and two-sample analyses. *Q* measures the observed extent of inter-SNV variability of ASE within a single gene (heterogeneity). The small reported *P*-values indicate genes with individual SNVs showing significantly inconsistent estimates of ASE. Such patterns can arise due to differences in ASE between various transcript isoforms of a gene [13], and therefore MBASED provides metrics for assessing the extent of isoform-specificity of the observed gene-level ASE.

Situations where one allele is favored in the read count data, even in absence of underlying ASE, have been reported in RNA-Seq data, due, for instance, to enrichment protocols, technological artifacts or a choice of a short read aligner [3,4]. We refer to such cases as instances of pre-existing allelic bias. When supplied with the values of probabilities of observing each allele at individual SNVs under conditions of no ASE, MBASED can incorporate such pre-existing biases into its estimates of ASE (Supplementary methods in Additional file 1), and we further provide functionality to estimate these probabilities from the data set itself. Our algorithm is implemented in the R [22] package MBASED. Further details are found in Materials and methods, Supplementary methods in Additional file 1, and the package vignette.

### Robust allele-specific expression detection by MBASED

To demonstrate the performance of MBASED in the absence of phasing information, we analyzed multiple sets of simulated data, in which artificially introduced allele-specific expression patterns were assigned to different genes at various allele preferences and expression levels. We selected a pair of matched tumor-normal samples from our panel (HCC individual 2) and recorded all of the detected exonic heterozygous SNVs in both samples, retaining information about the total RNA coverage of each SNV, while discarding the observed reference and alternative allele counts in each sample. We chose a real data set as the basis for our simulations to ensure that the simulated data sets had realistic distributions of both the number of heterozygous SNVs per gene and the read coverage per SNV.

**Figure 2:**
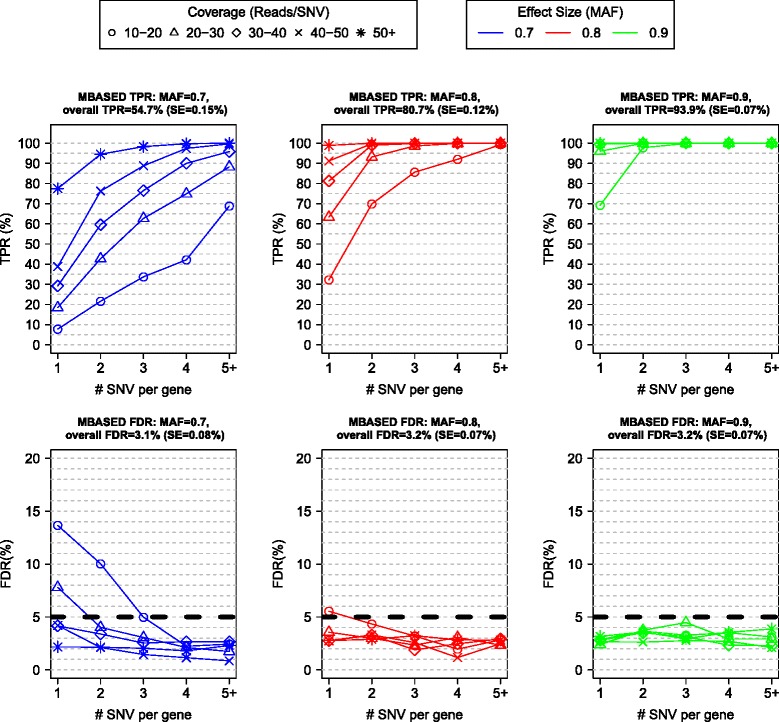
**Performance of MBASED on simulated data in one-sample analysis.** Genes were broken into 25 strata, based on number of SNVs in a gene and average number of reads per SNV. Within each strata 25% of genes were randomly chosen to exhibit ASE. For each SNV in a true positive ASE gene, one allele was randomly assigned to major haplotype and the corresponding read counts were simulated as described in Materials and methods. MBASED was run on the simulated data and genes with Benjamini-Hochberg adjusted *P*-values ≤0.05 (false discovery rate (FDR) control of 5%) were declared ASE. The average (across 100 simulations) true positive rate (TPR) and FDR within each strata and for each level of ASE signal (MAF used for ASE true positive genes) are shown. The overall TPR and FDR levels are obtained by giving each stratum weight proportional to the fraction of genes in that stratum (that is, these values are heavily weighted towards genes with few SNVs and low coverage, common in our data), and the average values are given in panel titles, along with their estimated standard errors (SE). MBASED performs very well at higher coverage levels and higher ASE extent.

We assessed the performance of the one-sample MBASED algorithm using the tumor sample. Briefly, we divided all tested genes in the sample into 25 strata based on 5 levels of each of the 2 covariates of interest: the number of SNVs in a gene (1, 2, 3, 4, or 5+) and the average coverage of SNVs in a gene (10 to 20, 20 to 30, 30 to 40, 40 to 50, or 50+ reads/SNV). The stratification was done to ensure that we tested MBASED across a variety of settings. Within each stratum, we randomly assigned a specified fraction *f* of the genes (for example, 25%) to be ASE true positives (TPs), and the rest of the genes were assigned to be ASE true negatives (TNs). We then simulated read counts for SNVs in ASE TN genes from the null distribution (MAF = 0.5), while for SNVs in ASE TP genes the counts were simulated from a signal distribution, where we varied signal strength (MAF) from 0.7 to 0.9 (Materials and methods). We then ran MBASED on each simulated data set and declared any gene with a Benjamini-Hochberg [23] (BH) adjusted *P*-value ≤0.05 to exhibit ASE. We performed 100 simulations for each combination of simulation settings, and Figure 2 illustrates average (over simulations) MBASED performance for *f* = 25%.

We found that the true positive rate (TPR) increased with read coverage and underlying ASE strength (MAF), as well as with the number of SNVs in a gene. We controlled the overall false discovery rate (FDR) at the nominal level of 5%, indicating that the *P*-value adjustment was effective. MBASED performed well even in low information settings. For example, >90% of ASE TP genes with 2 SNVs and 20 to 30 reads/SNV were recovered in simulations with MAF = 0.8. In analyzing real data, we required that a gene exhibit an estimated MAF ≥0.7 in addition to passing the statistical significance cutoff in order to be declared as exhibiting ASE (Materials and methods). As expected, this additional effect size cutoff reduced the TPR drastically for underlying ASE strength MAF = 0.7 (overall TPR fell from 55% to 37%), but had no appreciable effect on the TPR for higher values of MAF (data not shown).

**Figure 3:**
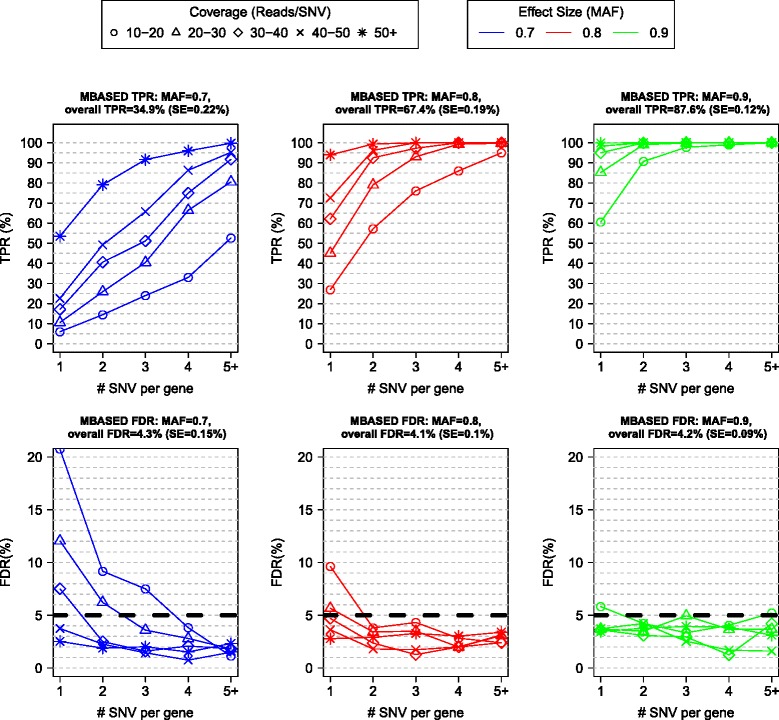
**Performance of MBASED on simulated data in two-sample analysis.** Simulations were performed similar to the one-sample case, as described in Materials and methods. MBASED was run on the simulated data and genes with Benjamini-Hochberg adjusted *P*-values ≤0.05 (false discovery rate (FDR) control of 5%) were declared ASE. The average (across 100 simulations) true positive rate (TPR) and FDR within each strata and for each level of ASE signal (MAF used for ASE true positive genes) are shown. The overall TPR and FDR levels are obtained by giving each stratum weight proportional to the fraction of genes in that stratum (that is, these values are heavily weighted towards genes with few SNVs and low coverage, common in our data), and the average values are given in panel titles, along with their estimated standard errors (SE). MBASED performs well at higher coverage levels and higher ASE extent, but its power is limited for the low-coverage, low-signal scenarios.

Similarly, we performed simulations in the two-sample setting (Figure 3; Materials and methods). We observed the dependence of the TPR on read coverage and the number of SNVs per gene similar to one-sample simulations, although for a given combination of simulation settings the two-sample method had somewhat lower power.

MBASED employs beta-binomial distribution to model read count data (Materials and methods), which accounts for extra-binomial variability (overdispersion) often observed in allelic counts in RNA-Seq data sets [4,13,14]. We used the levels of overdispersion similar to those observed in real data (Materials and methods) while performing simulations, and note that MBASED performance improves as the amount of overdispersion decreases and the separation between signal and noise distributions of test statistic increases (Figures S1 to S4 in Additional file 1).

We also tested the performance of MBASED in the setting of pre-existing allelic bias, by assuming that at each SNV under conditions of no ASE the probability of observing reference allele count, *P*_*ref*_, was >0.5 (global reference bias). We found the results to be very close to those observed in the no-bias simulations (Figures S5 and S6 in Additional file 1). We conclude that the MBASED method is robust in detecting ASE genes in samples with unknown true haplotypes, with detection power increasing with observed gene coverage and the number of detected heterozygous SNVs in a gene.

We further assessed the performance of MBASED in a situation where the true underlying haplotypes are known. We obtained previously published lymphoblastoid cell line RNA-Seq data and a list of phased genomic variants for (non-cancer) individual NA12878, genotyped together with both parents as part of the 1000 Genomes Project [12]. We pre-processed the data analogously to other samples in our panel (Materials and methods) and applied MBASED both with (‘phased’) and without (‘non-phased’) specifying the true haplotypes. Overall, we tested 2,560 genes for ASE, including 1,104 (40%) with >1 heterozygous loci. Using the cutoffs of 0.7 on estimated MAF and 0.05 on adjusted *P*-value, MBASED found 110 genes to show ASE in the ‘phased’ setting and 115 genes in the ‘non-phased’ setting, of which 108 were in common, indicating a high degree of consistency (Figure S7 in Additional file 1; Additional file 2). The small number of observed discrepancies was due to higher power of MBASED to detect ASE in general, and isoform-specific ASE in particular, when true haplotypes are known. A detailed discussion of the observed differences between running MBASED with and without prior knowledge of true haplotypes is provided in the Supplementary discussion in Additional file 1. We further note that running MBASED without supplying the true haplotypes resulted in correct haplotype reconstruction of 40/47 (85%) ASE genes with multiple SNVs. Further investigation revealed that of seven instances where haplotype reconstruction failed, six were likely due to alignment artifacts (Supplementary discussion in Additional file 1).

Finally, we compared the performance of MBASED with that of Skelly *et al*. [13], which is to our knowledge the only currently published ASE detection method that allows for variable ASE within a gene. Since the method of Skelly *et al*. requires that the true haplotypes be known, we used NA12878 RNA-Seq data for this comparison and supplied the true haplotypes to MBASED (Materials and methods). The method of Skelly *et al.* identified 103 ASE genes (posterior P(ASE) >0.95, posterior median MAF ≥0.7), compared to 110 identified by MBASED, including 94 that were common to both methods (Additional file 2). Of the nine genes identified as ASE by the method of Skelly *et al.* only, all have estimated MBASED MAF ≥0.8, but fall short of the significance cutoff due to low read coverage (10 to 12 reads/SNV, MBASED ASE *P*-values 0.05 to 0.17). Of the 16 genes identified as ASE by MBASED only, 15 show posterior P(ASE) >0.95 according to the method of Skelly *et al.*, with posterior median MAF values of 0.58 to 0.7. The lower MAF estimates of Skelly *et al.* are due to its no-ASE prior imposed on the data. A detailed discussion of the observed differences between MBASED and the method of Skelly *et al.* is provided in the Supplementary discussion in Additional file 1. We conclude that the two methods produce qualitatively and quantitatively similar results on this data set. We note, however, that MBASED can perform in situations when the true haplotypes are unknown, a major advantage over the method of Skelly *et al.* In addition, MBASED allows for the effects of pre-existing allelic bias and disambiguates the technical and biological contributions to overdispersion in the data (Materials and methods), while the method of Skelly *et al.* combines the two.

### Cancer samples exhibit high levels of allele-specific expression

We applied the MBASED method to a panel consisting of 18 lung cancer cell lines, 3 non-small cell lung cancer (NSCLC) tumor tissue samples, 4 hepatocellular carcinoma (HCC) tumor tissue samples, and 7 matched normal samples for the tumor tissues (Table S1 in Additional file 3) for a total of 25 cancer (21 lung and 4 liver) and 7 normal samples. None of the samples in the panel had known haplotypes. One-sample MBASED analysis was performed for each of the 32 samples and two-sample analysis was performed for tumor/normal and normal/tumor comparisons of 7 paired samples. Within each sample (or a pair of samples for two-sample analysis) only the genes containing informative heterozygous SNVs were tested for ASE (Materials and methods). Any gene with a BH adjusted MBASED *P*-value ≤0.05 and estimated MAF ≥0.7 was declared as exhibiting ASE in one-sample analysis. Similarly, any gene with a BH adjusted MBASED *P*-value ≤0.05 and estimated MAF difference ≥0.2 was declared as exhibiting sample-of-interest-specific ASE in two-sample analysis. This assignment provided one way of determining a set of genes in which to further characterize ASE in downstream analysis. All genes with adjusted inter-SNV ASE variability *P*-value ≤0.05 were flagged as possibly subject to isoform-specific ASE effects. Further details of the analysis pipeline are provided in Materials and methods, and the full results of MBASED application to the samples in our panel are available in Additional files 4 and 5. We note that the power of MBASED to detect mild levels of ASE is limited in the low coverage setting (right panels of Figures 2 and 3), common in our data, and the ASE levels reported here likely underestimate the true extent of ASE in samples under study.

**Figure 4:**
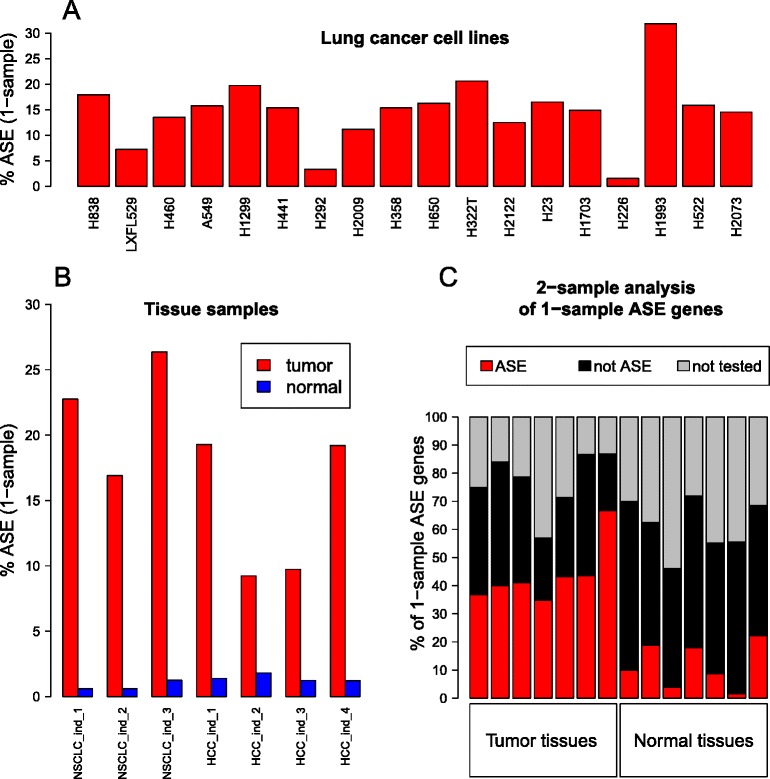
**Summary of ASE extent in the studied samples. (A)** Percentage of tested genes declared ASE in one-sample analysis of lung cancer cell lines. **(B)** Percentage of tested genes declared ASE in one-sample analysis of tissue samples (both tumor and normal). Considerably more genes are found to show ASE in tumor than in normal samples. **(C)** Tumor-specificity of ASE found in one-sample analysis of tissue samples. All genes found to be ASE in one-sample analysis are broken down into genes that are (a) not tested in a two-sample comparison (due to lack of common sufficiently covered SNVs, gray), (b) found to show ASE in two-sample comparison (red), and (c) not found to show ASE in two-sample comparison (black).

We found evidence for extensive ASE in the majority of cancer samples in the panel (Figure 4A,B). One-sample analysis revealed 9 to 26% of all tested genes in 7 tumor samples as showing ASE, considerably higher than the 0.5 to 2% ASE rate observed in 7 matched normal samples. The extent of ASE in lung cancer cell lines was highly variable (1 to 32%) and was correlated with the sample RNA-Seq coverage levels (data not shown). In contrast, no such correlation was observed for tissue samples, which had higher RNA sequencing depth (Table S1 in Additional file 3).

Of genes that exhibited ASE in the one-sample analysis of tumors and that also were tested for ASE in the two-sample analysis, 48 to 77% showed tumor-specific ASE (Figure 4C). By comparison, a much smaller fraction of genes showing ASE in one-sample analysis of normal samples were found to show normal-specific ASE (3 to 32%), despite higher RNA-Seq coverage of the normal sample in five out of seven sample pairs (Table S1 in Additional file 3). This indicates that while most of ASE observed in normal samples is retained in the tumor, a large fraction of the ASE observed in the tumors has developed during the tumorigenesis process.

Across our 32 samples, we found that in one-sample MBASED analysis 22 out of 2,080 ASE genes with multiple heterozygous SNVs showed evidence of isoform-specific ASE. We note that the significance test based on heterogeneity statistic *Q* has lower power in the settings of low read coverage and few SNVs, common in our data, and we likely underestimated the extent of isoform-specific ASE. Since 20 of these genes were found in the liver samples (7 in the normal, 13 in the tumor), there might be more isoform-specific ASE occurring in the liver, although none of these genes exhibited liver-specific expression. Alternatively, it is possible that we were hindered in our detection of isoform-specific ASE by the low sequencing depth, since liver samples had the highest RNA-Seq coverage in our data set. In the two-sample MBASED analysis, 16 out of 701 ASE genes with multiple heterozygous SNVs showed evidence of isoform-specific ASE, including 12 in the liver samples (11 in the normal, 1 in the tumor). The biological significance of the observed instances of isoform-specific ASE is unclear and is further complicated by the observation that 10 out of 22 genes with one-sample isoform-specific ASE and 5 out of 16 genes with two-sample isoform-specific ASE were represented by only one transcript isoform. This observation may be due to incompleteness of the current set of gene models or to the variance of SNV-level measures of ASE in those genes exceeding what is allowed by our statistical model.

Overall, the normal samples exhibited limited extent of ASE, using our chosen cutoffs, while the cancer samples showed much higher ASE rates, with isoform-specific ASE playing a limited role, if any.

### Allele-specific expression in cancer is linked to large-scale genomic changes

**Figure 5:**
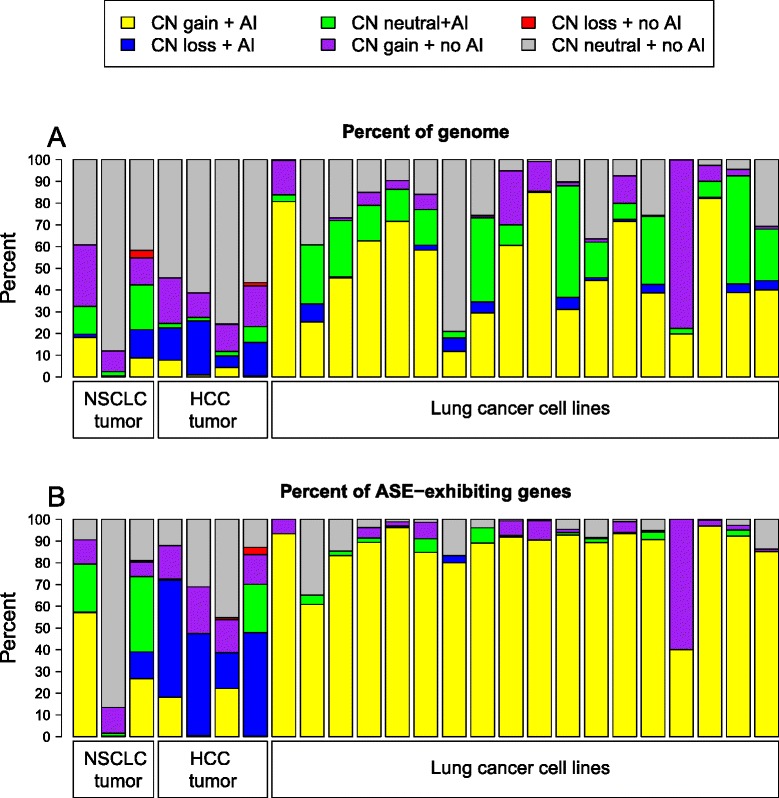
**Summary of genomic state of genes showing ASE in cancer samples. (A)** Proportion of autosomal genome falling into different categories of underlying copy number (CN) and allelic imbalance (AI) states. Cell lines show more CN gains and fewer CN losses than tissue samples. **(B)** Proportion of ASE autosomal genes falling into different categories of underlying CN and AI states. CN gain (cell lines) and loss (tissues) regions are enriched for ASE genes. The large extent of ASE genes in regions of CN loss in tissues is most likely explained by heterozygosity detection due to normal tissue admixture. The vast majority of ASE genes in tumor tissue samples from NSCLC individual 2 (second from left) fall into CN-neutral, no-AI regions, but the sample exhibits ASE levels comparable to the other two NSCLC patients (compare Figure 4B).

We assessed the copy number (CN) state and allelic imbalance (AI) at the DNA level for all cancer samples (Materials and methods). We found that a large fraction of observed ASE in cancer samples could be attributed to underlying changes in genomic composition. This observation has previously been reported in a single sample of oral cancer [15]. The profiles of these changes appeared to be markedly different between cell lines and tissue samples, with cell lines showing more genomic CN gains and AI (both in CN-gained and CN-neutral regions), but fewer CN losses than tumor tissue samples (Figure 5A). However, these observed differences might be due to different platforms used for CN and AI assessments of tissue samples and cell lines (Materials and methods). Genomic AI and CN changes accounted for >65% of ASE-exhibiting genes in all 18 cell lines (including 17 cell lines with >83%), and >55% of ASE-exhibiting genes in 6 out of 7 tumor tissue samples (Figure 5B), showing cancer ASE to be a phenomenon mainly driven by large-scale DNA alterations. The single exception among the tumor tissues came from NSCLC individual 2, which exhibited ASE in 17% of tested genes (similar to other cancer tissue samples; Figure 4B), but had 87% of these ASE-exhibiting genes fall outside of regions of CN alteration or detected AI. This suggests that alternative mechanisms for upregulation of ASE may exist in cancer (for example, allele-specific silencing through DNA methylation) and may be at play here. However, we cannot rule out the alternative possibility that the CN calling algorithm did not perform well on this sample. In 5 out of 7 tumor tissue samples, 6 to 25% of ASE-exhibiting genes fell into regions of CN loss, indicating that the detected ASE in those genes might be due to normal contamination or tumor heterogeneity, as no heterozygous variants should be detected in such regions in the absence of admixture.

### Recurrent instances of cancer-specific ASE point to regions of recurrent genomic alterations

**Figure 6:**
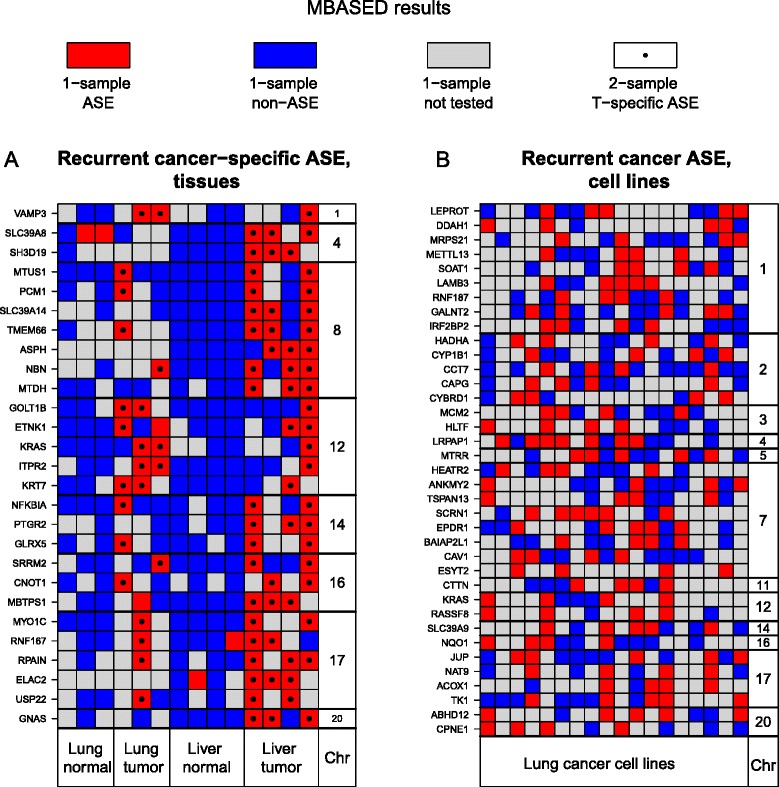
**Selected instances of recurrent cancer-specific ASE.** For both panels, columns are samples and rows are genes. Gene status in each sample is plotted. Note that the non-ASE category (blue) may include genes that fall just short of our ASE cutoffs, as well as genes where low coverage reduces our power to detect ASE. Genes in the non-tested category (gray) lack informative SNVs for ASE assessment in that sample. **(A)** Selected genes that show tumor-specific ASE (black dot) in multiple cancer tissue samples. Genes were chosen based on whether ASE was gained in tumor tissue samples relative to normal tissue samples, according to two-sample MBASED analysis. We require that the gain of ASE occurs in at least 3/7 tumor samples. Genes are grouped by chromosome (rightmost column) and ordered top-to-bottom in order of chromosomal location. Note that gene *RNF167* shows ASE in both normal and tumor samples in HCC individual 4; however, different haplotypes are overexpressed in the two samples. **(B)** Selected genes that show ASE in multiple lung cancer cell lines. Genes were chosen based on whether ASE was detected based on one-sample MBASED analysis. We require that the ASE occurs in at least 4/18 cell lines and does not occur in any of the 7 normal samples (to exclude, for example, imprinted genes). Genes are grouped by chromosome (rightmost column) and ordered top-to-bottom in order of chromosomal location.

We identified instances of cancer-specific ASE based on the MBASED two-sample analysis of tumor-normal pairs. A selection of such genes is shown in Figure 6A. Generally, genes with recurrent cancer-specific ASE tended to cluster together when found on the same chromosome. For example, chromosome 12 genes *ETNK1*, *GOLT1B* and *ITPR2* are located in close proximity to *KRAS* and we found the *KRAS*-containing segment of chromosome 12 to be lost in two samples and gained in another sample, while an additional two samples exhibited allelic imbalance of the entire chromosome 12 (Figure S8 in Additional file 1). As the result, all four genes (*ETNK1*, *GOLT1B*, *KRAS*, and *ITPR2*) were found to show recurrent cancer-specific ASE. In another example, all five chromosome 17 genes showing recurrent cancer-specific ASE were located on a portion of the chromosome with lower CN than the rest of chromosome 17 in all 7 tumor tissue samples (Figure S9 in Additional file 1). This frequently lost genomic segment also contained the known tumor suppressor gene *TP53*, consistent with the recurrent CN loss. In this instance, it is likely that normal admixture gave rise to detected heterozygous variants in these tumor samples, and that we would not detect any ASE under conditions of high tumor purity. In the case of chromosome 8, a segment was commonly present in a lower CN than the rest of the chromosome, but we were unable to definitively associate it with a known oncogenic driver. Finally, in some cases (for example, chromosomes 14 and 16) most of the chromosomes showed AI in multiple samples, giving rise to recurrent ASE. Genes with recurrent ASE in cancer cell lines also showed enrichment for certain chromosomes (Figure 6B). However, it was difficult to associate these recurrent events with common genomic aberrations, due to a considerably richer and more complicated pattern of CN alterations in cell lines (Figure 5A).

Based on these and similar observations, we conclude that the instances of recurrent ASE in our cancer samples were often driven by recurrent modifications of the underlying genomic CN state, affecting known driver genes in some cases.

### Selective overexpression of mutant alleles in cancer samples

We further investigated the interaction between the ASE and mutations in cancer. The ability of MBASED to perform ASE detection without prior knowledge of haplotype phasing allowed us to assess ASE of mutation-containing genes based on information from both SNPs and mutations. We then used MBASED haplotype calls to assign a mutation to the ‘major’ or ‘minor’ haplotype. From the 25 cancer samples, we identified 691 non-synonymous somatic (or putative somatic in the case of cell lines; Materials and methods) variants that are potentially ‘functional’, that is, variants that were classified as ‘deleterious’ by SIFT [24] or ‘damaging’ by PolyPhen [25] or were predicted to result in translation stop gain or loss by Variant Effect Predictor [26] (Table S2 in Additional file 3). Of these variants, 291 presented the mutant allele as major, including 41 that fell into ASE-exhibiting genes. Overexpressed functional mutant alleles included a number of known or suspected contributors to oncogenesis (Table 1).

**Table 1:** **Some instances of overexpressed functional mutant alleles**

**Gene**	**Mutations (sample)**	**Affected domain**	**CN**	**AI**	**ASE**		**Details**
					**MAF**	***P*** **-value (adj.)**	
*KRAS*	G12C (NSCLC ind. 1)	GTP-binding	Gain	Yes	0.87	<1e-6	Reported in COSMIC, known activating mutations
	G12C (H358)	GTP-binding	Gain	Yes	0.73	1e-2	
	G12C (H23)	GTP-binding	Gain	Yes	0.75	<1e-6	
	G12A (H2009)	GTP-binding	Gain	Yes	0.66	1e-1	Falls short of significance cutoffs
*EGFR*	Q787K (NSCLC ind. 2)	Kinase	Gain	No	0.74	2e-5	Both mutations in same sample, on same haplotype. L858R is reported in COSMIC
	L858R (NSCLC ind. 2)	Kinase	Gain	No	0.74	2e-5	
*RAD18*	Q59H (HCC ind. 1)	RING-type ZF	Gain	No	0.75	6e-5	Mutations in this motif results in hypersensitivity to mutagens
*EAF2*	S207F (HCC ind. 1)	Transactivation	Gain	No	0.77	2e-5	Tumor suppressor (inducer of apoptosis via p53)
*MTOR*	V1801G (H522)	FAT	Gain	Yes	0.88	5e-3	Mutated domain is a binding site for MTOR inhibitor
*MYH9*	K1248N (NSCLC ind. 2)	Coiled coil	Neutral	No	0.82	<1e-6	Outside of CN gain/loss or AI regions
*MYO18A*	R426C (HCC ind. 1)	Unannotated	Neutral	No	0.71	4e-2	
*TIMP1*	R136H (HCC ind. 2)	NTR	Neutral	No	0.89	<1e-6	
*FAS*	T319I (HCC ind. 4)	Unannotated	Neutral	No	0.87	7e-3	
*CCDC50*	T459A (H650)	Unannotated	Neutral	No	0.82	<1e-6	

Affected domain: based on canonical RefSeq transcript information and UniProt annotation. CN: genomic copy number status. AI: presence of genomic allelic imbalance. ASE MAF and *P*-value (adj.): MBASED-derived estimate of major haplotype frequency and the corresponding (adjusted) *P*-value.

We observed five instances of functional mutations that alter codon 12 of *KRAS*, a known oncogenesis-driving event [27]. In three out of five cases, the mutant allele was significantly over-represented, while in another instance (NSCLC cell line H2009) the over-representation was borderline significant (MAF = 0.66, BH adjusted *P*-value = 0.1). This suggests the selective pressure to produce a large number of constitutively activated forms of *KRAS*. We also observed the over-representation of known activating mutation L858R in the kinase domain of *EGFR* (as well as a novel mutation in the same domain in the same individual), and a mutation in the FAT domain of *mTOR*, a major regulator of cell-signaling pathways. The FAT domain is a binding site for the *mTOR* inhibitor *DEPTOR* [28], suggesting that this mutation might also be constitutively activating. The potential instances of overexpressed inactivating mutant alleles include a mutation in the transactivation domain of the tumor suppressor *EAF2* [29], and a mutation in the ring-finger motif of gene *RAD18*, which is involved in post-replication DNA damage repair [30].

Out of 41 instances of functional mutations with the mutant allele in the major haplotype of ASE-exhibiting genes, five fell outside of regions of genomic copy number change and/or allelic imbalance (Table 1, last 5 rows), including mutations in cancer-related genes *MYH9*, *TIMP1*, and *FAS*. However, it is unclear what the exact consequences of these mutations were for protein functionality, and what advantage to tumorigenesis, if any, was conferred by overexpression of the mutant allele.

The overexpression of mutant alleles that might confer some advantage to tumor cells was not universal. We found a small number of examples of functional mutant alleles that were expected to contribute to tumor phenotype but were not overexpressed. For example, cell line H441 contained a mutation in codon 12 of *KRAS*, but unlike the other four instances of this mutation (Table 1), this mutant allele was under-represented relative to the wild-type allele. We also found two instances of known activating mutations in residue 61 of *NRAS* [27], with no evidence of ASE in one case and strong evidence for overexpression of the wild-type allele in the other.

In summary, we found multiple examples of ASE where the overexpressed allele was either a known or suspected activating mutation in an oncogene or an inactivating mutation in a tumor suppressor gene. In almost all such cases the observed ASE arose from underlying DNA CN alterations. We observed some instances where the mutations expected to contribute to the cancer phenotype were underexpressed. It is possible that in such cases the mutation was crucial to early oncogenic processes, but that at later stages of tumor evolution the dependence of the cells on the mutant form of the protein was reduced.

### Tumor samples show loss of ASE in approximately 15% of normal ASE genes and elevated ASE on chromosome X

We observed 89 instances of monoallelic expression (ASE with estimated MAF >0.9) in 74 genes across 7 normal samples in our panel. In 36 of these instances (corresponding to 28 genes) the matched tumor sample contained at least one common informative SNV in that gene, enabling us to test these genes for tumor-normal allelic imbalance using the two-sample MBASED approach. We found that in 5 out of 36 tested cases (13.9%), the observed monoallelic ASE (MAE) was specific to a normal sample and was lost in a tumor (Figure S10A in Additional file 1), but there were no instances of recurrent loss of MAE in tumor tissue samples. One example was gene *ABP1* in HCC individual 4 (Figure S10B in Additional file 1). A previously described translocation event adjacent to *ABP1* in this sample [31] might be a contributing factor to the observed loss of MAE. We also found one instance of an MAE pattern reversed between normal and tumor, in the *BCL2L10* gene in HCC individual 4 (Figure S10C in Additional file 1). *BCL2L10* encodes a pro-apoptotic factor and has been implicated in 5-azacytidine resistance in acute myeoloid leukemia and myelodysplastic syndrome patients [32]. The two alleles differ by a pair of SNVs in a 3’ UTR, but it is not clear if the observed switch of ASE pattern was due to differential functional efficiency of the two alleles or if one of the alleles was more oncogenic.

Extending this analysis to all instances of ASE in normal samples, including non-monoallelic, we found 161 cases of ASE in normal samples that could be tested for tumor-normal allelic imbalance. In 30 (18.6%) cases, the observed ASE was normal-specific, with 16 (53.3%) such instances not attributable to underlying CN alterations, including four out of five loss-of-MAE cases. The extended analysis also did not reveal any genes with recurrent normal-specific ASE.

Loss of imprinting has been previously reported in cancer [33]. We cross-referenced a list of known imprinted genes [34] against the list of genes with loss of ASE in tumor samples, and found no instances of loss of imprinting (Figure S10A in Additional file 1). In general, we found that out of 55 known imprinted genes, only 7 could be tested for ASE in 3 or more normal samples. We found that two of these seven genes (*FAM50B* and *NDN*) showed monoallelic expression in all tested instances, while the other five genes (*GNAS*, *IGF2*, *NAA60*, *SLC22A18*, and *SLC22A3*) did not show any evidence of ASE. These observed patterns could be due to the previously reported tissue-specificity of imprinting [35].

In addition to imprinting, another known source of ASE is chromosome X inactivation in female cells. We found that all but one of our nine female samples showed much higher rates of ASE on chromosome X than in the rest of the genome (Figure S11 in Additional file 1; Fisher exact test *P*-value <0.02 for chromosome X versus autosomal ASE rate comparison for all eight samples). The sole exception was a female cell line, H2009, that suffered a loss of a copy of chromosome X and exhibited no ASE on that chromosome. We found that the rates of ASE in chromosome X genes in the two normal female tissue samples were low (<8%), consistent with the existence of several clonal lines in each sample, with different copies of the chromosome inactivated in different clones [17]. On the other hand, all female cancer samples (after excluding H2009) showed high rates of ASE on chromosome X (54 to 100% of tested genes). In some cases, including both female tumor tissue samples, this elevated rate could be attributed to underlying CN alterations. However, two of the cell lines did not show any CN changes or AI on chromosome X (data not shown), suggesting that a monoclonal expansion took place in these samples, giving rise to cell mixture, where one copy of chromosome X was preferentially silenced [17].

Overall, we observed a moderate extent of loss of normal ASE in tumors, with approximately 15% of normal ASE genes being normal-specific. We did not find any instances of recurrent loss of ASE and we also did not detect any instances of loss of imprinting. The observed loss of ASE did not appear to be driven by the underlying CN alterations, although the exact mechanism and biological significance of this process remain unclear. On the other hand, we observed elevated rates of ASE on chromosome X in cancer samples, occasionally accompanied by underlying genomic allelic imbalance. These latter cases might be due to the previously described high extent of chromosome X inactivation following monoclonal expansion. Our analysis was limited by a small sample size and low sequencing coverage. Larger-scale studies are needed to investigate these issues further.

## Conclusions

We developed a novel method, MBASED, for the detection of allele-specific gene expression, both in a single-sample analysis setting and in the context of two-sample comparison (differential ASE). MBASED leverages all available information to determine the extent of ASE in a given gene by combining evidence across SNVs within a gene using a meta-analysis-based approach. In our study, a high fraction of genes showed evidence of more than one heterozygous expressed SNV, highlighting the importance of having an information aggregation framework. For the eight liver tissue samples we have examined, which had the highest levels of both WGS and RNA-Seq coverage among our samples, 45% to 55% of the genes were multi-SNV, and we expect these higher percentages to be typical of all deeply sequenced samples.

A main advantage of MBASED is that it does not rely on known phasing information and is therefore capable of using both SNPs and mutations in the analysis. While the haplotype reconstruction approach employed by MBASED is far from robust, the use of internal simulations allows the assignment of proper statistical significance to the resulting estimates of ASE. Using a sample with known haplotypes as a control, we find that running MBASED without supplying the haplotype information leads to correct haplotype recovery for 40/41 multi-SNV ASE genes. Further, out of 115 genes declared to exhibit ASE when haplotypes are withheld, only 7 are not supported when haplotype information is taken into account. Of these, six genes either show ASE just below our significance cutoffs or are cases of spurious ASE likely due to alignment artifacts. These observations indicate that lack of knowledge of true haplotypes leads to a very minor increase in type 1 error rate.

The framework of MBASED supports both within-sample and between-sample ASE analyses. The latter functionality allows the user to, for example, identify differential ASE in tumor/normal comparisons, or to detect instances of ASE not attributable to DNA copy number changes in RNA versus DNA allelic comparisons. The meta-analytic approach taken by MBASED also allows the user to detect instances of isoform-specific ASE. Since MBASED is agnostic with respect to the unit of expression, future studies might look at measuring ASE of individual transcripts directly. Finally, the algorithm is capable of handling pre-existing allelic biases (for example, global reference bias due to enrichment protocol or alignment technique that favors the reference allele), without sacrificing performance.

A potential limitation of MBASED is its assumption of exactly two haplotypes of a gene, but there might be rare situations of a chromosomal duplication of a variant-containing gene followed by a further mutation, giving rise to three distinct haplotypes. In this case MBASED will then attempt to resolve SNV-level information into two haplotypes. Another limitation is the reliance of MBASED on various approximations when incorporating pre-existing allelic bias and overdispersion into the model (Supplementary methods in Additional file 1), but this might not be suitable for extreme values of allelic bias or overdispersion levels. Finally, unlike the one-sample MBASED approach, the two-sample MBASED algorithm does not utilize a variance-stabilizing transformation prior to employing meta-analytic data-combining procedure (Supplementary methods in Additional file 1), exposing the overall estimate of ASE to potential influence of outliers. Further work is needed to properly address these limitations.

Applying MBASED to a combined panel of cell lines and paired tissue-normal samples, we found a large extent of ASE in cancer samples, driven primarily by underlying changes in DNA CN or composition (AI). As the result, the observed instances of recurrent ASE could often be attributed to recurrent genomic alterations.

The ability of MBASED to include somatic mutations in the ASE analysis allowed us to look in depth at the expression patterns of mutant alleles. We discovered evidence for significant preferential expression of the activating allele in known oncogenes (for example, *KRAS* and *EGFR*). In the case of *KRAS*, we observed significant mutant allele overexpression in three out of five mutated samples, with another sample showing borderline significance, suggesting that the overexpression might be the result of positive selection in tumor evolution. Our analysis shows that in almost every instance (36 out of 41), the observed significant overexpression of a mutant allele that was predicted to have functional consequences in the protein product was due to a CN change or an allelic imbalance event in the underlying genomic regions. This suggests a limited role for alternative ASE mechanisms (for example, pre- or post-transcriptional suppression of transcripts carrying wild-type alleles) as drivers of overexpression events giving rise to cancer phenotype. Further work is needed to clarify whether the findings reported here are a general feature of the cancer landscape, and what role ASE plays in a variety of cancer tissue types and indications.

A number of technical biases may give rise to false positives when the ASE state of transcriptomes is assayed with RNA-Seq [3,5,8]. In an initial analysis we discovered a large number of genes that showed recurrent ASE across multiple samples, including some genes with monoallelic ASE in 20 or more samples. We investigated those genes in more detail and discovered that we could attribute ASE recurrence to various artifacts, which we subsequently eliminated from the data (Materials and methods; Supplementary methods in Additional file 1). In some instances, we believe that the observed recurrent ASE might be due to the errors on the part of the aligner (for example, if there exists a known highly homologous region in the genome). However, in other cases we found evidence that the detected heterozygous variants in the gene were due to the presence in the sample genome of a homologous nonexpressed region that was absent from the reference genome. Since most of those variants are reported in dbSNP (v.132), an investigator might be led to believe that such a gene is imprinted or shows monoallelic expression in a *cis*-determined fashion. Thus, care needs to be taken in order to prevent the detection of spurious instances of ASE, which are likely to dominate any list of recurrent ASE events.

In addition to presenting a new method for ASE detection in both one-sample and two-sample analyses, the current study presents, to the best of our knowledge, the most comprehensive look at ASE patterns in cancer to date. As more samples across different cancer types become available, a comprehensive picture of the extent and role of ASE in oncological diseases will emerge. Simultaneously, the patterns and role of ASE and imprinting in normal tissues will be elucidated and will in turn shed light on why these would be disrupted in cancer. However, until the sequencing technologies mature to the point of allowing the investigator to obtain uninterrupted sequences of entire transcripts, ASE calling methods will continue to rely on aggregating information across several loci. Therefore, the many advantages presented by the MBASED algorithm make it well-suited for the current stage of ASE studies of both normal and tumor samples.

## Materials and methods

### Data collection and processing

We performed WGS on 18 NSCLC cell lines and paired tumor-normal samples from 3 NSCLC patients, as well as 4 paired tumor-normal samples from 4 HCC patients, using Complete Genomics technologies [36], for a total of 7 normal and 25 cancer samples. All 32 samples have been previously published [31,37]. We performed RNA-Seq (75 or 76 bp paired-end) using Illumina GA-IIx for all samples, and the resulting short reads were aligned to the hg19 version of human genome using the GSNAP algorithm [38]. All duplicate reads have been reduced to a single copy in order to avoid the detection of spurious ASE due to biases in PCR amplification steps of sequencing protocol. CN and AI information on the 18 cell lines was assayed with Illumina OMNI 2.5 M SNP array and processed with a modified version of PICNIC algorithm [37]. This analysis produced integer estimates of total and major allele CN in the segmented genome. Any region with total CN >2 was declared to be a region of CN gain, while any region with total CN <2 was declared to be a region of CN loss. Similarly, any region with (Major allele CN)/(Total CN) > 0.5 was declared to be in the state of AI. In addition, CN status and AI information for the seven tumor tissue samples relative to the paired normal samples was inferred from WGS data using a dedicated pipeline, as previously described [31,37]. Detailed information about the samples is provided in Table S1 in Additional file 3.

For each sample, we obtained a list of called SNVs from the output of the Complete Genomics processing pipeline and tabulated the reference and alternative allele counts at each SNV from the aligned DNA and RNA reads. We eliminated potential homozygous SNVs by requiring that both the reference and alternative allele be supported by at least five WGS reads each as well as by at least 10% of all WGS reads aligned to that SNV. To avoid spurious SNV calls due to nearby indels [8], we also eliminated any SNVs falling within 10 bp of another variant. SNVs were assigned to RefSeq genes and only exonic SNVs were retained, with any SNVs falling into exons of more than one gene discarded. We further required that SNVs be covered by at least 10 reads in RNA-Seq data to ensure sufficient power to detect ASE and any excessive inter-loci variability. If multiple SNVs were overlapped by common WGS or RNA-Seq reads, they were merged into a single locus to ensure that the observed read counts at individual SNVs (loci) of a gene were independent, as required by the statistical model.

A number of authors have reported the existence of false positive ASE calls produced by various biases in short read aligners [3,4]. In order to filter out potential alignment artifacts we adopted some additional filters based on Self Chain alignments of the genome to itself [39-41], the reported frequency of structural variants in genomic regions provided in the Database of Genomic Variation [42,43], and on frequency of detected variants within a gene (see Supplementary methods in Additional file 1 for in-depth discussion of the filtering pipeline). The selfChain alignments and DGV variants were downloaded from the UCSC Genome Browser database [44] on 29 November 2012.

For tissue samples, somatic mutations in cancer samples were identified based on WGS using the CallDiff tool provided by Complete Genomics. For cell lines, a filter based on a large database of known common variants was applied to SNVs and any SNV passing the filter was declared ‘putative somatic’, as previously described [37].

Variant consequences were obtained for each SNV and each affected transcript with Variant Effect Predictor [26]. Effect predictors SIFT [24] and PolyPhen [25] were used to identify deleterious variants among the SNVs. Any variant predicted to be deleterious by either SIFT or PolyPhen or predicted to result in stop codon gain/loss by Variant Effect Predictor was declared to have a possible effect on protein function (‘functional’). The affected protein domains were determined by consulting the UniProt database [45].

RNA-Seq data for NA12878 individual was obtained from the Gene Expression Omnibus (GEO) using accession number GSE30401 [12]. Only FASTQ files corresponding to paired-end data were used. Since all sequencing was done on the same Illumina flow cell, the individual lane sequencing results were pooled. Phased genomic variants in hg19 coordinates were downloaded from [46]. The data were aligned and filtered analogously to our own panel of samples (see Supplementary discussion in Additional file 1 for detailed description).

A set of known imprinted genes was downloaded from geneimprint.com [34] on 15 October 2012.

### Statistical methods

#### One-sample analysis

We employ the gene as a unit of ASE, which we define as a union of all exons forming individual transcript isoforms. We assume that for each gene with at least one heterozygous exonic SNV there are exactly two haplotypes, and at each such SNV we observe *n*_*SNV*_ total RNA-seq reads, with *x*_*ref*,*SNV*_ reads mapping to the reference allele and *x*_*alt*,*SNV*_ reads mapping to the alternative allele such that *x*_*ref*,*SNV*_ + *x*_*alt*,*SNV*_ = *n*_*SNV*_. For gene *g*, we denote the true underlying frequencies of transcript haplotypes by *p*_*hap*1,*g*_ and *p*_*hap*2,*g*_. MBASED models haplotype 1 allele-supporting counts at individual SNVs as:

$$ {X}_{hap1, SNV}\sim \mathrm{Beta}\hbox{-} \mathrm{Binomial}\left( n={n}_{SNV},\mu ={f}_{hap1, SNV}\left({p}_{hap1, g}\right),\rho ={\rho}_{SNV}\right), $$

where

$$ E\left({X}_{hap1, SNV}\right)= n\mu, $$

and

$$ \mathrm{var}\left({X}_{hap1, SNV}\right)=\mu \left(1-\mu \right) n\left(\rho \left( n-1\right)+1\right). $$

We use a beta-binomial model as an alternative to a standard binomial model in order to incorporate extra-binomial dispersion observed in RNA-Seq data [13]. We parametrize the beta-binomial model in terms of the mean parameter *μ* and overdispersion parameter *ρ*. In the case of no overdispersion (*ρ* = 0), the read counts are binomial random variables:

$$ {X}_{hap1, SNV}\sim \mathrm{Binomial}\left( n={n}_{SNV}, p={f}_{hap1, SNV}\left({p}_{hap1, g}\right)\right) $$

If the frequency of reads supporting each gene haplotype reflects the true transcriptome-level haplotype proportions, then:

$$ {f}_{hap1, SNV}\left({p}_{hap1, g}\right)={p}_{hap1, g}. $$

If, however, the read count-generating probabilities are affected by the sequencing and/or alignment protocols, then in general:

$$ {f}_{hap1, SNV}\left({p}_{hap1, g}\right)\ne {p}_{hap1, g}. $$

For example, if the alignment protocol is more likely to align reference-supporting read,

then:

$$ {f}_{hap1, SNV}\left({p}_{hap1, g}\right)>{p}_{hap1, g}, $$

if the haplotype 1 allele is reference, and

$$ {f}_{hap1, SNV}\left({p}_{hap1, g}\right)<{p}_{hap1, g}, $$

if the haplotype 1 allele is alternative. We refer to such situations as instances of pre-existing allelic bias. We find that in our data set the reference allele is consistently somewhat over-represented relative to the expected fraction of 0.5 (Figure S12 in Additional file 1), indicating mild levels of pre-existing allelic bias favoring the reference allele. MBASED is capable of estimating both the extent of pre-existing allelic bias (*f*_*hap*1,*SNV*_(0.5)) and overdispersion (*ρ*_*SNV*_) in the data, and we describe the estimation strategy we employed for samples in this study in Supplementary methods in Additional file 1. For clarity of presentation, we describe in this section the behavior of MBASED under the settings of no pre-existing allelic bias and no overdispersion:

$$ {X}_{hap1, SNV}\sim \mathrm{Binomial}\left( n={n}_{SNV}, p={p}_{hap1, g}\right), $$

and provide the details of the full model in Supplementary methods in Additional file 1.

We employ the techniques of meta-analysis to combine information across SNVs within a single gene and derive a gene-level measurement of ASE. This approach is more robust than simply summing up the reads across individual haplotypes, since it takes into account the varying depth of coverage across SNVs and allows for adjustment due to potential local biases. The meta-analysis framework in its unmodified state presupposes the correct haplotype reconstruction for the validity of statistical inference. When the true haplotypes are unknown (as is the case in our data), we employ a ‘voting’ phasing algorithm. This pseudo-phasing produces the major and minor haplotypes by assigning SNV-level alleles with higher RNA read counts to the same ‘major’ haplotype and alleles with lower RNA read counts to the ‘minor’ haplotype.

Under the assumption that, for a given haplotype (haplotype 1) of a gene, the haplotype-specific SNV-level allele counts *X*_*hap*1,*SNV*_ are independent Binomial(*n*_*SNV*_, *p*_*hap*1,*g*_) random variables, one can use meta-analysis to assess the ASE of that gene as follows. SNV-level haplotype 1 read counts are transformed using a variance-stabilizing Freeman-Tukey (FT) transformation [47] into FT values:

$$ {z}_{hap1, SNV}= FT\left({x}_{hap1, SNV},{n}_{SNV}\right)= si{n}^{-1}\left(\sqrt{\frac{x_{hap1, SNV}}{n_{SNV}+1}}\right)+ si{n}^{-1}\left(\sqrt{\frac{x_{hap1, SNV}+1}{n_{SNV}+1}}\right), $$

which under the assumptions stated above are approximately normally distributed:

$$ {Z}_{hap1, SNV}\sim N\left(2 si{n}^{-1}\left(\sqrt{p_{hap1, g}}\right),\frac{1}{n_{SNV}+0.5}\right) $$

A gene-level ‘average’ FT value, *z*_*hap*1,*g*_, is obtained as the inverse-variance-weighted average of SNV-level FT values *z*_*hap*1,*SNV*_. Under the null hypothesis of no ASE, we have *p*_*hap*1,*g*_ = 0.5, and a nominal meta-analysis-derived *P*-value is assigned to observed *z*_*hap*1,*g*_, based on the known mean and variance of corresponding random variable *Z*_*hap*1,*g*_. This nominal *P*-value is not used, however, for reasons described below. Backtransformation [48] is used to obtain a gene-level estimate of the haplotype frequency (MAF), which we call *T*_*FT*_, from *z*_*hap*1,*g*_ (Figure 1A). Further, we calculate inter-SNV ASE variability statistic *Q*, which measures the extent of heterogeneity between ASE measures *z*_*hap*1,*SNV*_ at individual SNVs within gene *g*, and the corresponding *P*-value is reported by MBASED. Small heterogeneity *P*-values point to potential instances of isoform-specific ASE (note that our default model assumes no ASE differences among individual transcript isoforms, hence a single gene-level parameter *p*_*hap*1,*g*_).

This straightforward application of meta-analysis needs to be modified in order to be used by MBASED, when the true underlying haplotypes are unknown. The ‘voting’ pseudo-phasing approach adopted by MBASED results in major haplotype allele counts *x*_*maj*,*SNV*_ that do not follow *X* ~ Binomial (*n*_*SNV*_, 0.5) distribution under the null model of no ASE. Instead, the correct distribution is that of

$$ X\hbox{'}= max\left\{ X,{n}_{SNV}- X\right\}, $$

which leads to anti-conservative nominal meta-analysis *P*-values. To account for this assumption violation, we simulate reference allele reads for each SNV in each tested gene from *X* ~ Binomial (*n*_*SNV*_, *P*_*ref*_ = 0.5) distribution, re-perform the pseudo-phasing of the SNV-level alleles into major and minor gene haplotypes using our voting approach, and obtain a meta-analysis-derived gene-level MAF estimate. Repeating this procedure *N*_*sim*_ times (we let *N*_*sim*_ = 10^6^) for each gene, we obtain the estimate of the distribution of meta-analysis estimates of MAF under the null hypothesis. Letting *T*_*FT*_ denote the MAF estimate for gene *g* based on observed ‘major’ haplotype counts, and letting ${T}_{si{ m}_j, FT}$ for *j* = 1, …, *N*_*sim*_ denote the *N*_*sim*_ MAF estimates from simulated data sets, we define the final significance score (*P*-value) for gene *g* as:

$$ {p}_{g, ASE}=\frac{\#\left({T}_{si{ m}_j, FT}\ge {T}_{FT}\right)}{N_{si m}} $$

Thus, we treat the meta-analysis estimate of MAF, *T*_*FT*_, as a statistic of interest and estimate its null distribution to obtain the corrected ASE *P*-value *p*_*g*,*ASE*_ (Figure 1B). We similarly obtain the *P*-value for heterogeneity of ASE across individual SNVs as

$$ {p}_{g, heterogeneity}=\frac{\#\left({Q}_{si{ m}_j}\ge Q\right)}{N_{si m}}. $$

In the case where there is only one heterozygous SNV identified for a gene, we use an approximation to the two-sided binomial exact test. Briefly, we transform the major allele SNV count using Freeman-Tukey transformation into an FT value and then backtransform to obtain an estimate of MAF. This leads to a natural MAF estimate *x*_*maj*,*SNV*_/*n*_*SNV*_. Similar to the multi-SNV scenario, we treat this MAF estimate as a statistic and estimate its null distribution through simulations. With a large number of simulations, the resulting ASE *P*-value *p*_*g*,*ASE*_ can become arbitrarily close to the *P*-value from the two-sided binomial exact test. Our motivation for adopting this alternative test was to ensure the consistency within our method with respect to single- and multi-SNV genes.

Using the same motivation, we also adopt a simulation-based approach to calculate *P*-values even if the phasing is known. In such instances, no additional phasing is done and major allele frequency is defined simply as the larger of the two observed allele frequencies.

#### Two-sample analysis

The meta-analysis framework extends naturally to the two-sample comparison aiming to detect sample-specific ASE. We describe the procedure here in terms of comparing a ‘tumor’ sample to a ‘normal’ sample, but the comparison can be done for any two samples.

As with one-sample analysis, we assume that there are exactly two distinct haplotypes for each tested gene, and that these haplotypes are the same for the two samples. We assume that at each heterozygous SNV of gene *g*, the sample-specific detected haplotype 1 allele counts *X*_*hap1*,*SNV*,*tumor*_ and *X*_*hap1*,*SNV*,*normal*_ follow Binomial(*n*_*SNV*,*tumor*_, *p*_*hap1*,*g*,*tumor*_) and Binomial(*n*_*SNV*,*normal*_, *p*_*hap1*,*g*,*normal*_) distributions, respectively. The extensions to the model, which take pre-existing allelic bias and overdispersion into account, are discussed in Supplementary methods in Additional file 1. We assume that SNV-level counts within a gene are independent, and we further assume that SNV-level counts are independent between the two samples. We choose as our measure of ASE the haplotype frequency difference:

$$ D={p}_{hap1, g, tumor}-{p}_{hap1, g, normal} $$

Under the null hypothesis of no tumor-specific ASE, we have:

$$ {p}_{hap1, g, tumor}={p}_{hap1, g, normal}={p}_{hap1, g}, $$

and therefore *D* = 0. Note that it is not necessarily the case that *p*_*hap*1,*g*_ = 0.5 under this null hypothesis. We then define SNV-level PD values (for proportion difference) as:

$$ {Z}_{hap1, SNV}=\frac{X_{hap1, SNV, tumor}}{n_{SNV, tumor}}-\frac{X_{hap1, SNV, normal}}{n_{SNV, normal}}. $$

Under the specified assumptions, *Z*_*hap*1,*SNV*_’s are approximately normally distributed:

$$ {Z}_{hap1, SNV}\sim N\left(0,\frac{p_{hap1, g, tumor}\left(1-{p}_{hap1, g, tumor}\right)}{n_{SNV, tumor}}+\frac{p_{hap1, g, normal}\left(1-{p}_{hap1, g, normal}\right)}{n_{SNV, normal}}\right) $$

We now follow the procedure previously described for a one-sample analysis, and obtain gene-level PD value *z*_*hap*1,*g*_, which we use as an estimate of *D* and as a test statistic *T*_*PD*_ (analogous to *T*_*FT*_ in the one-sample case). We also calculate inter-SNV ASE variability statistic *Q* and use the resulting *P*-value to assess evidence for isoform-specific between-sample allelic imbalance.

MBASED modifies this procedure in order to deal with unphased data as follows. Only heterozygous SNVs detected in both samples are used to assess sample-specific ASE. For each gene, the SNVs are phased into ‘major’ and ‘minor’ haplotypes by the voting algorithm based exclusively on the data from the tumor sample. The phasing is the same for both samples to ensure that we are comparing the same haplotypes and is based on the tumor because it should be more informative in the cases of true tumor-specific ASE. We then estimate ASE using the meta-analysis procedure and treating ‘major’ haplotype as the tested ‘haplotype 1’. As before, simulations are employed to account for the bias introduced by the violations of binomial distribution assumptions due to phasing. Using these simulations, null distributions of *T*_*PD*_ and *Q* are estimated, and the final ASE *P*-value *p*_*g*,*ASE*_ and heterogeneity *P*-value *p*_*g*,*heterogeneity*_ are obtained for each gene *g*. As part of this process, the underlying *p*_*hap*1,*g*_ under the null hypothesis of no sample-specific ASE is estimated (Supplementary methods in Additional file 1) and used as (beta-)binomial probability of success in simulations.

Note that using MBASED to detect tumor-specific ASE may also uncover some instances of normal-specific ASE. This may happen if the haplotype phasing based on the tumor happens to recover the true underlying haplotypes and the tumor-derived ‘minor’ haplotype is strongly overexpressed in the normal sample. We stress, however, that the proper way to identify such genes is by treating the normal sample as the sample of interest.

The details of both one-sample and two-sample analyses as well as a detailed description of the modifications to this approach in presence of known (or estimated) overdispersion and pre-existing allelic bias are provided in Supplementary methods in Additional file 1.

#### MBASED

We implemented the one- and two-sample analyses described above in the R package MBASED. In one-sample analysis, MBASED takes the reference and alternative allele RNA-Seq counts supplied by the user, as well as corresponding aseIDs (gene or transcript names) and haplotype assignments (if available) and reports for each aseID the estimated major haplotype frequency (MAF), the ASE *P*-value, and the inter-loci ASE variability *P*-value for multi-SNV genes. In two-sample analysis, MBASED takes the reference and alternative allele counts for both samples, as well as the corresponding aseIDs and reports the estimated MAF difference, the ASE *P*-value, and the inter-loci ASE variability *P*-value for multi-SNV genes. MBASED does not use any information about DNA (either WGS or WES) read counts, as ASE calls are based entirely on RNA data. The DNA-level data should be used during pre-processing steps to identify heterozygous exonic SNVs, according to the user’s criteria. If desired, a two-sample comparison can be performed by treating the RNA-Seq data as ‘tumor’ and DNA data as ‘normal’ to identify instances of allelic imbalance in the transcriptome not accounted for by the genomic allelic imbalance. However, this latter approach requires that both DNA and RNA-Seq data sets be produced under very similar conditions to avoid any systematic biases. Optionally, MBASED will estimate pre-existing allelic bias at each SNV from the data supplied by the user (or use directly supplied values) to properly adjust the reported effect size estimates and *P*-values. Similarly, user-supplied data can be used to estimate overdispersion at each SNV (or the values of the overdisperson parameter of beta-binomial distribution can be directly supplied). In our analysis we assumed that pre-existing allelic bias was the same at each SNV within a sample, and used a single estimate of the overdispersion parameter for all SNVs across all samples. Note that MBASED does not do any *P*-value adjustment for multiple hypothesis testing, and this task is left to the user. In the work presented here, we employed BH adjustment, but any other standard approach may be used. Further details are available in Supplementary methods in Additional file 1 and the package vignette.

#### Assignment of ASE status to genes

Within each sample (or a pair of samples in the case of tumor-normal comparisons) only the genes containing at least one heterozygous exonic SNV passing our filtering criteria were tested for ASE. To declare a gene to be exhibiting ASE, we set the cutoffs on both statistical significance and the estimated extent of ASE (effect size). In one-sample analyses, we employed the BH procedure to adjust ASE *P*-values provided by MBASED within each sample for multiple hypothesis testing and require candidate ASE-exhibiting genes to have adjusted *P*-values ≤0.05 (FDR control of 5%). In practice, we encountered many instances of genes that showed statistically significant ASE but exhibited an allelic ratio insufficiently distinct from 1:1 to warrant biological significance. This was often the case for genes with high RNA-Seq coverage, where we were able to detect even small departures from equal allele expression. Therefore, we required that a candidate gene show estimated MAF ≥0.7 in order to be declared as exhibiting ASE.

In two-sample analyses, we required candidate ASE genes to have BH-adjusted ASE *P*-values ≤0.05 and to exhibit estimated MAF difference (Tumor - Normal) ≥ 0.2 (this is similar to requiring MAF to be ≥0.7 (=0.5 + 0.2) in the one-sample scenario). Additionally, we required genes exhibiting ASE in the two-sample analysis to also show evidence of ASE in the one-sample analysis.

In both one-sample and two-sample analyses, any genes showing significant evidence of inter-loci variability (BH-adjusted inter-loci ASE variability *P*-value ≤0.05) were flagged as possibly exhibiting isoform-specific ASE.

It should be stressed that ASE is not a binary characteristic but inherently presents as a spectrum within the transcriptome. Our assignment provided one way of determining a set of genes in which to further characterize ASE in downstream analyses, and we do not attach any strong biological meaning to our cutoffs.

### Simulations

We chose tumor/normal samples from HCC individual 2 as the basis for our simulations, since they showed comparable WGS and RNA-Seq coverages. For each sample we retained all SNVs that were tested for ASE in our main analysis.

We assessed the performance of the one-sample MBASED algorithm on the tumor sample. All tested genes were divided into 25 strata based on 2 covariates: the number of SNVs in a gene (1, 2, 3, 4, or 5+ SNVs) and the average coverage of SNVs in a gene (10 to 20, 20 to 30, 30 to 40, 40 to 50, or 50+ RNA-Seq reads) to ensure that we tested MBASED across a variety of settings. Within each stratum, we randomly assigned a specified fraction *f* of the genes (for example, 25%) to be ASE true positives, and the rest of the genes were assigned to be ASE true negatives. For each SNV in the ASE true negative genes, we simulated reference allele counts from the distribution:

$$ \mathrm{Beta}\hbox{-} \mathrm{Binomial}\left( n={n}_{SNV},{\mu}_{ref}=0.5,\rho =0.004\right) $$

We chose the value 0.004 for overdispersion parameter *ρ* since it is the overdispersion estimate we used in our main analysis (Supplementary methods in Additional file 1). In contrast, for each SNV in the ASE true positive genes, we randomly assigned one allele (allele A) to the major haplotype and generated allele A counts from the distribution:

$$ \mathrm{Beta}\hbox{-} \mathrm{Binomial}\left( n={n}_{SNV},{\mu}_A=\mathrm{MAF},\rho =0.004\right) $$

We varied the value of MAF (major haplotype frequency), which measures the strength of ASE, from 0.7 to 0.9 across simulations, but kept it constant across SNVs within an individual simulation. We then ran MBASED on each simulated data set and declared any gene with an adjusted *P*-value ≤0.05 to exhibit ASE. We performed 100 simulations for each combination of *f* and MAF, and calculated average TPR and FDR within each stratum, as well as across all strata.

To assess the performance of the two-sample MBASED algorithm we generated read counts in simulated tumor samples as before and generated reference read counts at each SNV in simulated normal samples from the distribution:

$$ \mathrm{Beta}\hbox{-} \mathrm{Binomial}\left( n={n}_{SNV, normal},{\mu}_{ref}=0.5,\rho =0.004\right) $$

We also tested the performance of MBASED in the settings of global reference allele bias by letting probability of observing reference allele under conditions of no ASE *f*_*ref*,*SNV*_(0.5) = 0.6, or, equivalently, *f*_*alt*,*SNV*_(0.5) = 0.4. We then generated reference allele counts in the no-ASE settings using *μ*_*ref*_ = *f*_*ref*,*SNV*_(0.5), and major allele (allele A) counts using:

$$ {\mu}_A=\frac{\mathrm{MAF}\times {f}_{A, SNV}(0.5)}{\mathrm{MAF}\times {f}_{A, SNV}(0.5)+\left(1-\mathrm{MAF}\right)\times \left(1-{f}_{A, SNV}(0.5)\right)} $$

See Supplementary methods in Additional file 1 for discussion of the functional form of *μ*_*A*_ in the presence of pre-existing allelic bias. We find the results to be very close to those observed in the no-bias simulations (Figures S5 to S6 in Additional file 1).

Finally, we tested the performance of MBASED at varying levels of overdispersion. We find that performance declines for high values of overdispersion (Figures S1 to S4 in Additional file 1), so care needs to be taken when applying MBASED to datasets with high noise levels.

### Comparison with the method of Skelly *et al.*

The method of Skelly *et al.* [13] was run according to their tutorial. We followed the lead of the authors in using estimates a = 3,600 and d = 550 for the prior parameters. We used the posterior median of p as estimate of allele frequency and max(p, 1-p) as estimate of major allele frequency. We calculated the posterior P(ASE) as described in the tutorial. We declared any gene with posterior P(ASE) >0.95 and estimated MAF ≥0.7 as showing ASE. Detailed discussion of the comparison is provided in the Supplementary discussion in Additional file 1.

### Data availability

All 18 lung cancer cell lines and 3 paired tumor-normal NSCLC tissue samples have been previously published [37], and the sequencing data (WGS and RNA-Seq) are available from the NCBI database of Genotypes and Phenotypes (dbGaP) [49] under accession number phs000299. The SNP array data for lung cancer cell lines is available from NCBI GEO [50] under accession number GSE40908. The four paired tumor/normal HCC samples have been previously published [31] and the sequencing data (WGS and RNA-Seq) are available from dbGaP under accession number phs000384.

## Additional files

Additional file 1:
**Supplementary methods: describes the details of SNV filtering pipeline and MBASED algorithm.** Supplementary discussion: detailed comparisons between ‘phased’ and ‘non-phased’ modes of MBASED and between MBASED and the method of Skelly *et al.* [13]. The file also contains Figures S1 to S22. Additional file 2: Data set S1. The results of running one-sample MBASED algorithm in ‘phased’ and ‘non-phased’ modes, as well as the method of Skelly *et al.* [13] on RNA-Seq data for the NA12878 individual. Additional file 3: Table S1. Description of the samples used in this study. **Table S2.** detailed information about ASE calling of genes containing functional somatic mutations. Additional file 4: Data set S2. The results of running one-sample MBASED algorithm on all 32 samples in our study. Additional file 5: Data set S3. The results of running two-sample MBASED algorithm on all seven tumor/normal and all seven normal/tumor comparisons for the paired samples in our study.

## Abbreviations

AI: allelic imbalance
ASE: allele-specific expression
BH: Benjamini-Hochberg
bp: base pair
CN: copy number
FDR: false discovery rate
FT: Freeman-Tukey
GEO: Gene Expression Omnibus
HCC: hepatocellular carcinoma
MAE: monoallelic expression
MAF: major allele (haplotype) frequency
NSCLC: non-small cell lung cancer
RNA-Seq: RNA sequencing
SNP: single nucleotide polymorphism
SNV: single nucleotide variant
TN: true negative
TP: true positive
TPR: true positive rate
UTR: untranslated region
WES: whole exome sequencing
WGS: whole genome sequencing
