# Supplementary material for: MBASED: allele-specific expression detection in cancer tissues and cell lines
Source: Genome Biol. 2014 Aug 7;15(8):405. doi: 10.1186/s13059-014-0405-3 (PMC4165366; doi:10.1186/s13059-014-0405-3)
Supplement: Additional file 1: — Supplementary methods: describes the details of SNV filtering pipeline and MBASED algorithm. Supplementary discussion: detailed comparisons between ‘phased’ and ‘non-phased’ modes of MBASED and between MBASED and the method of Skelly et al. [13]. The file also contains Figures S1 to S22. [file 13059_2014_405_MOESM1_ESM.pdf]

MBASED: allele-specific expression detection in cancer tissues and cell lines.
Supplementary Materials

Oleg Mayba, Houston N. Gilbert, Jinfeng Liu, Peter M. Haverty, Suchit Jhunjhunwala, Zhaoshi Jiang, Colin Watanabe, Zemin Zhang

Contents

Supplementary Methods 3

1 SNV Filtering 3

2 MBASED Details 4

2.1 Model assumptions and MBASED outline . . . 4
2.1.1 Meta-analysis . . . 5
2.1.2 Generative statistical models for read counts . . . 6
2.1.3 Pre-existing allelic bias . . . 7
2.1.4 Use of DNA read counts in ASE analyses . . . 9
2.2 1-Sample Analysis . . . 9
2.2.1 Freeman-Tukey transformation . . . 10
2.2.2 Multi-SNV genes . . . 10
2.2.3 Single-SNV genes . . . 13
2.2.4 Adjustment for pre-existing allelic bias . . . 14
2.2.5 Estimating $f_{hap1,SNV}(0.5)$ . . . 16
2.2.6 Adjustment for technical overdispersion . . . 16
2.2.7 Estimating ρ_{SNV} . . . 18
2.3 2-Sample Analysis . . . 19
2.3.1 Multi-SNV genes . . . 20
2.3.2 Estimation of $p_{maj,g}$ . . . 23
2.3.3 Single-SNV genes . . . 24
2.3.4 Adjustment for pre-existing allelic bias . . . 25
2.3.5 Adjustment for technical overdispersion . . . 27

Supplementary Discussion 30

3 MBASED Performance in Absence of Phasing Information 30

4 Comparison of MBASED to the Method of Skelly et al. 33

Supplementary Figures 37

Supplementary Tables 63

Supplementary Data Sets 63

Supplementary Methods

1 SNV Filtering

Numerous studies of ASE that utilized RNA-Seq data have reported spurious signals due to various technical biases[1, 2, 3]. For example, early read-mapping algorithms tended to favor the alignment of reads containing reference allele, since such alignments incurred no mismatch penalties. While this particular bias can be overcome by SNP-tolerant alignment tools[3, 4], other sources of bias are more difficult to account for. With regards to alignment procedures, some authors simulated all possible SNP-covering reads and aligned them to the reference in order to identify SNPs that can give rise to artificial ASE even under ideal conditions[1, 5]. However, it is far from clear how this procedure can be extended to the paired-end protocols. Besides, as will be seen, this approach cannot filter out all sources of artificial ASE.

Spurious ASE has also been reported for SNVs in close proximity to indels[2, 6] and in low-complexity or repetitive regions[2, 7]. In some instances investigators reported detected ASE at a location found to be represented by 3 distinct haplotypes[2], an obvious artifact.

We adopted a filtering pipeline designed to discard SNVs that are likely to contribute to spurious ASE. First, we eliminated potential homozygous SNVs by requiring that at least 5 reads and at least 10% of all reads support each allele in WGS data. Second, we discarded all SNVs within 10 bp of another called variant to eliminate possible false variant calls due to alignment. Third, we discarded all SNVs in highly repetitive genomic regions, which we defined as regions with sequence identity of >95% to another genomic region based on selfChain Link track from UCSC Genome Browser[8, 9, 10, 11].

The above steps were designed to filter out SNVs that might give rise to artificial ASE due to inadequacies of alignment or variant-calling procedures. However, we discovered that some of the most prominent cases of spurious ASE could be attributed to incomplete reference genome rather than the problems with adopted data processing tools. Figure S13A shows 20 genes most frequently exhibiting ASE prior to filtering. Closer scrutiny revealed most of them to be artifactual. For example, one of the top most recurrently allele-specifically expressed gene *MAP2K3* exhibited high density of heterozygous SNVs in WGS data in all samples, but only one of those SNVs had the alternative allele detected in RNA data (Figure S14). A closer look revealed that there seemed to be 2 haplotypes of *MAP2K3* supported by WGS: one corresponding to reference genome and another containing virtually all alternative alleles at the detected SNVs. We further observed that 36 out of 37 *MAP2K3* SNVs in our data were found in dbSNP[12] (v.132). One explanation for this observation is all of our samples were simultaneously heterozygous for these two haplotypes. A more likely explanation is that all of our samples contained a nonexpressed region in their genome with homology to *MAP2K3* that was absent from the reference genome, indicating a possible assembly error in the reference genome. It is likely then that the observed sequence differences between *MAP2K3* and the putative homologous region were previously erroneously identified as SNPs within *MAP2K3* sequence, explaining their presence in dbSNP. Another example of similar nature was provided by gene *SEC22B* (Figure S15). Here we observed evidence for 3 distinct haplotypes at DNA level in all of our samples, only one of which is detected at RNA level.

Such artifacts will not be filtered out by the alignment of simulated reads, since the absence of the existing homologous regions from the reference genome will force false SNP identification. In fact, reference[1] reported *SEC22B* as one of their ASE genes after filtering out artifactual

SNPs. It should be noted that these artifacts will also not be filtered out by using DNA counts for normalization, since haplotypes presented at RNA and DNA levels are distinct. We therefore attempted to remove such regions from the analysis based on two proxy measures. First, we quantified the extent of detected variants in a gene by calculating the number of variants per kb of exonic length (VPKE) for each gene in each sample. Within each sample, we identified genes with highest 2% VPKE values and removed them entirely from further analysis. Our reasoning was that genes exhibiting very high number of variants (normalized for exonic length) might correspond to unannotated homologous regions described above. Secondly, we removed any SNVs that fall into genomic segments that were found to contain structural variations in 5 or more different samples in Database of Genomic Variation[13, 14, 11]. The effect of our filtering schema on the 20 genes most frequently exhibiting ASE is shown in Figure S13B. Of the top 20 genes, only the known monoallelically expressed gene *ERAP2* does not lose any of its SNVs to our filtering procedure. We believe that similar filtering pipelines will remain necessary in ASE studies to avoid spurious calls until all of the biases have been successfully addressed.

2 MBASED Details

Here we describe the statistical framework used by the MBASED (**M**eta-analysis-**B**ased **A**llele-**S**pecific **E**xpression **D**etection) algorithm for identifying instances of allele-specific gene expression in RNA count data. In 1-sample analyses, MBASED identifies within-sample deviations from the null hypothesis of equal allele expression, while for 2-sample analyses, differences in allelic ratios between samples with the same haplotypes are detected. Two-sample comparisons are therefore meaningful for samples from the same individual (paired analysis), but not necessarily for samples from different individuals. MBASED uses principles of meta-analysis to combine information across loci within a single unit of expression (which we call an `aseID`, e.g., a gene, a transcript, or an individual SNV). The informative loci are usually heterozygous SNVs, but could also be aggregations of SNVs that have been merged into a single locus. In the latter case, the true underlying haplotypes of the locus with respect to constituent SNVs must be known or calculated (e.g., by a formal phasing algorithm or examining reads overlapping multiple SNV locations). In the following discussion, we will use 'gene' in place of aseID and 'SNV' in place of 'locus', as these are the units we typically use in practice.

2.1 Model assumptions and MBASED outline

We assume that for each tested gene in a sample (i.e., a gene with at least one heterozygous SNV), there are exactly two distinct haplotypes. For each gene g, one can model the detected haplotype 1 allele read counts $X_{hap1,SNV}$ at each SNV as independent $F(n_{SNV}, p_{hap1,g}, \theta_{SNV,g})$ random variables, where

- F is some choice of discrete probability distribution, e.g., binomial
- n_{SNV} is the total number of reads covering a particular SNV
- $p_{hap1,g}$ is the true underlying frequency of haplotype 1 transcripts for that gene (with $p_{hap2,g} = 1 - p_{hap1,g}$)
- $\theta_{SNV,g}$ denotes any additional parameters for specific model choices

Under the null hypothesis of no ASE, the two haplotypes are expressed at the same rate, and therefore $p_{hap1,g} = p_{hap2,g} = 0.5$. Note that we explicitly assume that there is no difference in ASE between individual transcript isoforms of gene g (hence a single haplotype frequency parameter $p_{hap1,g}$). We address the issue of isoform-specific ASE detection in section 2.1.1.

In practice, the assumption of independence can be grossly violated if two heterozygous SNVs are close enough to each other to be largely covered by the same set of reads. In such cases the two SNVs should ideally be merged into a single locus (we will continue to refer to such merged loci as 'SNV' for clarity of presentation).

2.1.1 Meta-analysis

In most studies to date, ASE testing was done at the level of individual SNVs, even if more than 1 heterozygous SNV per gene was present[15, 1, 16, 17, 18, 19, 7]. Most of the time, only the agreement of final SNV-level calls is observed, and no attempt is made to share information across SNVs or to assign weights to SNV-specific calls based on the calls' confidence (e.g., amount of read coverage). In some instances, when haplotypes are phased, the SNV-level counts are added up across haplotypes[2, 5]. This, however, masks potential ASE heterogeneity due to alternative splicing.

We describe how meta-analysis techniques, following[20] (DerSimonian-Laird), can be employed to test a multi-SNV gene for ASE based on observed SNV-specific allele read counts. These techniques were initially developed to aggregate information across multiple studies of the same phenomenon. Study-specific measurements are aggregated by taking their inverse variance-weighted average to provide an overall estimate and a corresponding measure of confidence (e.g., a confidence interval or a p-value for deviation from some null value of overall effect).

MBASED adopts this approach to the problem of combining ASE information across individual SNVs into a gene-level measure of ASE. In the 1-sample analysis, we choose a haplotype (haplotype 1) and test whether it is over- ($p_{hap1,g} > 0.5$) or under- ($p_{hap1,g} < 0.5$) represented relative to the other haplotype (haplotype 2) based on allelic counts at individual SNVs. In the 2-sample analysis, we test whether the haplotype frequencies are different between the two samples ($p_{hap1,g,sample1} \neq p_{hap1,g,sample2}$). We report the estimate of ASE exhibited by the gene and the corresponding p-value.

Furthermore, in the DerSimonian-Laird meta-analytic framework, a p-value based on a constancy test statistic Q measures the extent of heterogeneity of observed ASE at SNVs within a gene. These heterogeneity p-values are also reported by MBASED, with small p-values indicating different levels of underlying ASE at individual SNVs, which may be attributed to isoform-specific ASE (if the unit of ASE is a gene) or some other source of excessive variability in ASE measurements.

The meta-analysis framework in its unmodified state presupposes the correct haplotype reconstruction for the validity of statistical inference. However, in practice, we do not have phasing information for any of our samples, a situation typical of currently available RNA-Seq studies. We therefore designed MBASED to handle both datasets with and without phasing information.

When true haplotypes are unknown, one can, in principle, attempt to phase SNVs into haplotypes based on population databases and prior linkage disequilibrium information[21]. However, this approach is restricted to known SNPs and does not allow for easy phasing of mutations, particularly somatic mutations arising in tumor samples.

Instead, in abasence of haplotype information, we phase individual SNVs into gene haplotypes based on observed read counts. In such cases, we perform Monte Carlo simulations based on the

null hypothesis of no ASE ($p_{hap1,g} = 0.5$ in 1-sample analysis, $p_{hap1,g,sample1} = p_{hap1,g,sample2}$ in 2-sample analysis) to assign statistical significance to the resulting measures of gene-level ASE. The final reported p-values are then based on the results of these simulations.

2.1.2 Generative statistical models for read counts

The binomial model is widely used for ASE detection in count data[1, 17, 2, 19, 22, 7]. In the simplest version of the model, the detected haplotype 1 allele read counts are modeled as

$$X_{hap1,SNV} \sim Bin(n = n_{SNV}, p = p_{hap1,g}) \tag{1}$$

random variables. Sometimes, pre-existing allelic bias is present, which results in over-representation of reads supporting a particular allele even under conditions of no ASE. In such cases, it is common to modify model (1) by incorporating the underlying bias into the generative binomial probability:

$$X_{hap1,SNV} \sim Bin(n = n_{SNV}, p = f_{hap1,SNV}(p_{hap1,g})) \tag{2}$$

We discuss the issue of pre-existing allelic bias and its incorporation into the model in detail in section 2.1.3.

A number of authors have advocated the use of beta-binomial model to accomodate extra-binomial variability (overdispersion) observed in real RNA-Seq data. This additional variability can arise due to various technical biases[6], aggregation of data across individuals[5], or heterogeneity of ASE across SNVs in the same gene due to alternative splicing[23]. In the first two cases, the additional variability is a nuisance parameter to be accounted for, while in the last case it might provide evidence of isoform-specific ASE, a potentially interesting biological finding. MBASED follows the lead of these authors in adopting a beta-binomial model, and attempts to separate the two sources of overdispersion by estimating the inter-SNV ASE variability ('isoform effect') after accounting for overdispersion due to technical effects. This approach is different from either ignoring the distinction and treating the biological and technical overdispersion jointly[5, 6], or assuming different dispersion parameters for genes with and without ASE[23]. In the latter case, the dispersion estimate in a gene with variable ASE is an aggregated measure of both technical and isoform effects, and does not distinguish between the two.

We say that $X \sim BetaBin(n, \mu, \rho)$, if $X \sim Bin(n, Y)$ and $Y \sim Beta(\mu, \rho)$, where $\mu = E(Y) \in (0, 1)$ and $\rho = \frac{var(Y)}{\mu \times (1-\mu)} \in (0, 1)$. In this parametrization, ρ is the overdispersion parameter, with the convention that $\rho = 0 \Rightarrow P(Y = \mu) = 1$ (leading to $X \sim Bin(n, \mu)$, standard binomial model). We then have

- $E(X) = n\mu$
- $var(X) = \mu(1-\mu)n\rho(n-1+\frac{1}{\rho})$, which reduces to $n\mu(1-\mu)$ if $\rho = 0$, the variance of binomial distribution.

In the ASE setting, one can model the detected haplotype 1 allele counts $X_{hap1,SNV}$ at each SNV within gene g as independent random variables with

$$X_{hap1,SNV} \sim BetaBin(n = n_{SNV}, \mu = p_{hap1,g}, \rho = \rho_{SNV}) \tag{3}$$

If $\rho_{SNV} = 0$, the model reduces to the binomial model (1). When pre-existing allelic bias is present,

model (3) can be modified as:

$$X_{hap1,SNV} \sim BetaBin(n = n_{SNV}, \mu = f_{hap1,SNV}(p_{hap1,g}), \rho = \rho_{SNV}) \tag{4}$$

This is the full statistical model supported by MBASED. By default, MBASED assumes that there is no pre-existing allelic bias and no overdispersion in the data, reducing model (4) to model (1). The user can supply the information on pre-existing allelic bias and overdispersion or allow MBASED to estimate it from the data, in order to take advantage of the full model (4).

Some authors estimate the parameter ρ_{SNV} within an individual gene ($\rho_{SNV} = \rho_g$), using Bayesian prior distribution assumptions, and allowing ρ_g to capture overdispersion due to both technical effects and isoform-spedific ASE[23]. Other authors let genes share the overdispersion parameter ($\rho_{SNV} = \rho$), modeling ρ as capturing overdispersion due to technical effects, the extent of which is assumed to be constant across all loci within a sample[5, 6].

In the model adopted by MBASED, ρ_{SNV} represents nuisance overdispersion, due to technical effects only. In other words, the default MBASED model (4) assumes that there is no additional overdispersion due to biological events of interest, e.g., isoform-specific ASE. The evidence for the existence of this additional overdispersion is evaluated separately, as described below, allowing MBASED to separate the two sources of extra-binomial variability. In practice, MBASED requires the values ρ_{SNV}, representing technical overdispersion, to be specified for each locus in the sample, and therefore cases of SNV-specific (no relationship between ρ_{SNV}), gene-specific ($\rho_{SNV} = \rho_g$), and sample-specific ($\rho_{SNV} = \rho$) modeling restrictions on ρ_{SNV} are handled within the same framework. While MBASED provides functionality to estimate ρ (or ρ_g, or ρ_{SNV}), it requires the user to supply the data to be used in such estimation. In other words, MBASED relies entirely on the user for the estimates of ρ_{SNV}, and it is important that these estimates capture the entire extent of technical overdispersion. We assume in what follows that the appropriate values of ρ_{SNV} have been supplied to MBASED, and we will describe in section 2.2.7 the estimation approach that we adopted in this study.

2.1.3 Pre-existing allelic bias

We discuss the pre-existing allelic bias in the context of binomial generative model, and the interpretation in the case of the beta-binomial model is entirely analogous. We introduce the notation $f_{hap1,SNV}(p_{hap1,g})$ in specifying model (2), with $0 \leq f_{hap1,SNV}(p_{hap1,g}) \leq 1$, to highlight that the probability of observing a read supporting haplotype 1 at a given locus may not be the same as the underlying haplotype frequency $p_{hap1,g}$. This may occur when the RNA enrichment protocol or the choice of alignment procedure results in allelic bias. For example, an alignment procedure may favor reads containing the reference allele[1]. In such instances the SNVs where the haplotype 1 allele is reference will exhibit $f_{hap1,SNV}(p_{hap1,g}) > p_{hap1,g}$, and, conversely, the SNVs where the haplotype 1 allele is alternative will exhibit $f_{hap1,SNV}(p_{hap1,g}) < p_{hap1,g}$. Note that the probability of observing a read supporting haplotype 2 is

$$f_{hap2,SNV}(p_{hap2,g}) = 1 - f_{hap1,SNV}(p_{hap1,g}) = 1 - f_{hap1,SNV}(1 - p_{hap2,g}) \tag{5}$$

In the binomial model shown in (2), the probabilities of observing the two haplotype alleles under the conditions of no ASE are given by $f_{hap1,SNV}(0.5)$ and $f_{hap2,SNV}(0.5)$. Whenever $f_{hap1,SNV}(p_{hap1,g}) \neq p_{hap1,g}$, we say that pre-existing allelic bias is present in the data. When assessing deviations from null hypothesis of no ASE ($p_{hap1,g} = 0.5$), most authors assume no pre-

existing allelic bias, while others estimate $f_{hap1,SNV}(0.5)$, either through simulations[1], or from genomic DNA (where $p_{hap1,g} = p_{hap2,g} = 0.5$, under heterozygous diploid conditions)[22, 23].

MBASED and the analysis presented in this study assume a particular functional form of $f_{hap1,SNV}$. Namely, we assume that

$$f_{hap1,SNV}(p_{hap1,g}) = \frac{f_{hap1,SNV}(0.5) \times p_{hap1,g}}{f_{hap1,SNV}(0.5) \times p_{hap1,g} + f_{hap2,SNV}(0.5) \times p_{hap2,g}} \tag{6}$$

where

- $p_{hap1,g}$ and $p_{hap2,g}$ are the true underlying frequencies of haplotype 1 and haplotype 2 transcripts, with $p_{hap1,g} + p_{hap2,g} = 1$
- $f_{hap1,SNV}(0.5)$ and $f_{hap2,SNV}(0.5)$ are underlying frequencies of observed haplotype 1 and halpotype 2 allele-supporting reads under the conditions of no ASE, with $f_{hap1,SNV}(0.5) + f_{hap2,SNV}(0.5) = 1$ from equation (5)
- $f_{hap1,SNV}(p_{hap1,g})$ and $f_{hap2,SNV}(p_{hap2,g})$ are the true underlying frequencies of observed haplotype 1 and haplotype 2 allele-supporting reads

We note that

- $f_{hap1,SNV}(p_{hap1,g})$ is proportional to both $p_{hap1,g}$ and $f_{hap1,SNV}(0.5)$ and is uniquely determined by these two frequencies, an approch similar to the one employed by[22]
- $f_{hap1,SNV}(p_{hap1,g} = 0.5) = \frac{f_{hap1,SNV}(0.5)\times 0.5}{f_{hap1,SNV}(0.5)\times 0.5 + f_{hap2,SNV}(0.5)\times 0.5} = f_{hap1,SNV}(0.5)$, as required
- $f_{hap1,SNV}(p_{hap1,g}) + f_{hap2,SNV}(p_{hap2,g}) = 1$, as required
- If $f_{hap1,SNV}(0.5) = 0.5$ (no allelic bias under conditions of no ASE), then $f_{hap1,SNV}(p_{hap1,g}) = p_{hap1,g}$ (no allelic bias under any ASE setting)

To illustrate the logic behind our approach, consider a situation where an alignment procedure favors the reference allele over the alternative allele, with the result that, in the absence of ASE, we observe reference allele 60% of the time, on average. Suppose now that we have a situation of genuine ASE (say, $p_{hap1,g} = 0.8$), and that at the SNV of interest, the allele supporting haplotype 1 is reference. Then letting $p_{ref} = 0.6$ and $p_{alt} = 1 - p_{ref} = 0.4$ denote the probabilities of observing the reference and alternative alleles, respectively, under conditions of no ASE, we have

$$f_{hap1,SNV}(p_{hap1,g}) = \frac{p_{hap1,g} \times p_{ref}}{p_{hap1,g} \times p_{ref} + p_{hap2,g} \times p_{alt}} = \frac{0.8 \times 0.6}{0.8 \times 0.6 + 0.2 \times 0.4} = 0.86$$

and

$$f_{hap2,SNV}(p_{hap2,g}) = \frac{p_{hap2,g} \times p_{alt}}{p_{hap2,g} \times p_{alt} + p_{hap1,g} \times p_{ref}} = \frac{0.2 \times 0.4}{0.2 \times 0.4 + 0.8 \times 0.6} = 0.14$$

Similarly, if the allele supporting haplotype 1 at our SNV is alternative, we have

$$f_{hap1,SNV}(p_{hap1,g}) = \frac{p_{hap1,g} \times p_{alt}}{p_{hap1,g} \times p_{alt} + p_{hap2,g} \times p_{ref}} = \frac{0.8 \times 0.4}{0.8 \times 0.4 + 0.2 \times 0.6} = 0.73$$

and

$$f_{hap2,SNV}(p_{hap2,g}) = \frac{p_{hap2,g} \times p_{ref}}{p_{hap2,g} \times p_{ref} + p_{hap1,g} \times p_{alt}} = \frac{0.2 \times 0.6}{0.2 \times 0.6 + 0.8 \times 0.4} = 0.27$$

Notice that we are more likely to observe the major allele if it is reference ($p = 0.86$) than if it is alternative ($p = 0.73$), even though the underlying true major haplotype frequency in the transcriptome is the same (0.8).

In practice, MBASED requires the values $f_{hap1,SNV}(0.5)$, representing pre-existing allelic bias, to be specified for each locus in the sample. While MBASED provides functionality to estimate $f_{hap1,SNV}(0.5)$, it requires the user to supply the data to be used in such estimation. We assume in what follows that the appropriate values of $f_{hap1,SNV}(0.5)$ have been supplied to MBASED, and describe in section 2.2.5 the estimation approach that we adopted in this study.

2.1.4 Use of DNA read counts in ASE analyses

A number of studies have used allele-specific genomic DNA (gDNA) counts to adjust RNA-based measures of ASE for any technical biases that may give rise to artifactual allelic imbalance. This practice is commonly employed in microarray studies, where both gDNA and RNA-derived cDNA molecules are hybridized to the same array type[18], but it can also be done in the sequencing setting[17, 16, 19, 22, 23]. In both cases, one has to assume that any biases introduced into the measures of ASE by the experimental procedure are shared by the DNA and RNA samples. In our case, DNA and RNA read counts were derived from different sequencing platforms (CGI and Illumina, respectively) and we do not believe that a direct comparison of RNA and DNA allelic ratios would be appropriate. However, one can easily compare RNA and gDNA samples directly using the 2-sample version of MBASED to specifically detect genes showing ASE in excess of what can be explained by genomic allelic imbalance alone. In this study we are interested in all instances of ASE in cancer samples, including the ones arising from copy number alterations, and therefore do not perform this comparison. In addition, some authors have used gDNA counts to estimate parameters of the assumed distribution of RNA counts. One example of this is using gDNA counts to estimate overdispersion parameter of the beta-binomial distribution[23]. This is most appropriate when RNA and DNA samples are sequenced and aligned using as similar protocols as possible. For this reason, while we do adopt a beta-binomial model, we do not employ gDNA to estimate over-dispersion in the current study. However, MBASED allows the user to supply gDNA counts for this purpose, as described in the package vignette.

2.2 1-Sample Analysis

We assume that for gene g in the sample of interest, the number of reads supporting haplotype 1 at an exonic heterozygous SNV $X_{hap1,SNV}$ follows a beta-binomial model (4), and we denote the observed value of this random variable as $x_{hap1,SNV}$. In 1-sample analysis, our measure of ASE is the major haplotype frequency (MAF) defined as $p_{maj,g} = \max(p_{hap1,g}, p_{hap2,g})$, and we want to test the null hypothesis of no ASE

$$H_0 : p_{maj,g} = 0.5 \tag{7}$$

For clarity of presentation, we first describe the MBASED algorithm under the assumption of binomial read counts ($\rho_{SNV} = 0$) and of no pre-existing allelic bias ($f_{SNV}(p_{hap1,g}) = p_{hap1,g}$),

reducing the model to

$$X_{hap1,SNV} \sim Bin(n = n_{SNV}, p = p_{hap1,g}) \tag{8}$$

We then demonstrate the extensions to take pre-existing allelic bias into account ($f_{SNV}(p_{hap1,g}) \neq p_{hap1,g}$, section 2.2.4) and to include overdispersion ($\rho_{SNV} \neq 0$, section 2.2.6).

2.2.1 Freeman-Tukey transformation

In our application of DerSimonian-Laird meta-analytic framework to the 1-sample ASE analysis, we largely follow the approach implemented in the function `metaprop()` in R package `meta`[24]. This implementation applies meta-analysis to binomial (but not beta-binomial) counts through the use of the variance-stabilizing Freeman-Tukey (FT) transformation[25], briefly described here. Let $X \sim Bin(n, p)$, and let x denote an observed value of X. Then

$$Z = FT(X, n) = \sin^{-1}\left(\sqrt{\frac{X}{n+1}}\right) + \sin^{-1}\left(\sqrt{\frac{X+1}{n+1}}\right) \tag{9}$$

is an approximately $N\left(2\sin^{-1}(\sqrt{p}), \frac{1}{n+0.5}\right)$ random variable, with an observed value $z = FT(x, n) \in (0, \pi)$. Note that the (approximate) variance of Z does not depend on p, but the (approximate) mean does, illustrating the variance-stabilizing properties of the transformation. The companion backtransformation $P = FT^{-1}(z, n)$[26] takes z and the original total count n and produces the proportion $0 \leq P \leq 1$. The backtransformation is exact in the sense that it gives back the observed proportion: $FT^{-1}(FT(x, n), n) = x/n$.

2.2.2 Multi-SNV genes

Consider a gene with several heterozygous exonic SNVs, and suppose that phasing information is available, i.e., for each SNV we know which allele belongs to haplotype 1 and which to haplotype 2. We choose a haplotype (say, haplotype 1) and convert the observed haplotype 1 read counts using the Freeman-Tukey transformation into corresponding FT-values,

$$z_{hap1,SNV} = FT(x_{hap1,SNV}, n_{SNV}) \tag{10}$$

Under the assumptions of binomial model (8), the tranformed values $z_{hap1,SNV}$ are realizations of independent (approximately) normal random variables $Z_{hap1,SNV}$, with

$$E(Z_{hap1,SNV}) \approx 2\sin^{-1}(\sqrt{p_{hap1,g}}) \tag{11}$$

In particular, under conditions of no ASE ($p_{hap1,g} = 0.5$), we have $E(Z_{hap1,SNV}) = \pi/2$. Further,

$$var(Z_{hap1,SNV}) \approx \frac{1}{n_{SNV} + 0.5} \tag{12}$$

While the expressions for mean and variance of $Z_{hap1,SNV}$ given above are approximate, the approximation is known to be quite good as long as $E(X_{hap1,SNV})$ is not too close to 0 or n_{SNV} (i.e., we expect at least few counts to support each allele).

We then compute a gene-level FT-value, $z_{hap1,g}$, as

$$z_{hap1,g} = \frac{\sum_{SNV} w_{SNV} \times z_{hap1,SNV}}{\sum_{SNV} w_{SNV}} \tag{13}$$

where

$$w_{SNV} = var^{-1}(Z_{hap1,SNV}) = \frac{1}{n_{SNV} + 0.5} \tag{14}$$

Thus, $z_{hap1,g}$ is the inverse variance-weighted mean of the $z_{hap1,SNV}$'s, where the weights $\left(\frac{w_{SNV}}{\sum_{SNV} w_{SNV}}\right)$ are normalized to add up to 1. Note that $z_{hap1,g}$ is the observed value of the random variable

$$Z_{hap1,g} = \frac{\sum_{SNV} w_{SNV} \times Z_{hap1,SNV}}{\sum_{SNV} w_{SNV}} \tag{15}$$

Since for each SNV $E(Z_{hap1,SNV}) = 2\sin^{-1}(\sqrt{p_{hap1,g}})$ and $var(Z_{hap1,SNV}) = \frac{1}{w_{SNV}}$, we have

$$E(Z_{hap1,g}) = 2\sin^{-1}(\sqrt{p_{hap1,g}}) \tag{16}$$

and

$$var(Z_{hap1,g}) = \frac{1}{\sum_{SNV} w_{SNV}} \tag{17}$$

Note that the contribution of each individual observation (SNV) is weighted according to coverage (total number of reads), and this weight is independent of the ASE estimate at that SNV (proportion of reads supporting haplotype 1 allele).

We also calculate DerSimonian-Laird constancy statistic

$$Q = \sum_{SNV} w_{SNV}(z_{hap1,SNV} - z_{hap1,g})^2 \tag{18}$$

where w_{SNV} is given in (14). Q (also known as Cochran's heterogeneity Q) measures heterogeneity among SNV-level ASE estimates, and under the hypothesis of homogeneity ($p_{hap1,SNV} = p_{hap1,g}$ is constant across SNVs within gene g), the distribution of Q is approximately χ^2 with (#SNVs-1) degrees of freedom. The resulting heterogeneity p-value is recorded, with small p-values indicating evidence for isoform-specific ASE or some other source of excessive variability. We note that tests for heterogeneity based on Q have lower power in the common setting of few SNVs and low coverage, and the reader is warned that MBASED might be under-powered to detect instances of true heterogeneity in those situations.

To obtain the gene-level estimate of $p_{hap1,g}$, we use the Freeman-Tukey backtransformation

$$\widehat{p}_{hap1,g} = FT^{-1}(z_{hap1,g}, n_g) \tag{19}$$

where n_g stands for some gene-level average of n_{SNV}'s. In some rare cases (and depending on the choice of n_g) it may happen that $z_{hap1,g} < FT(0, n_g)$ or $z_{hap1,g} > FT(n_g, n_g)$. In other words, the transformed value is outside of the range attainable for a given n_g. One way this may happen is for one SNV to show very low coverage n_{SNV_1} and very low frequency of haplotype 1-supporting reads (z_{hap1,SNV_1} close to 0), while a second SNV shows very high coverage $n_{SNV_2} >> n_{SNV,1}$ and very high frequency of haplotype 1-supporting reads (z_{hap1,SNV_2} close to π). In this scenario, it may be possible for the gene-level value $z_{hap1,g}$ to be so close to π as to be outside of the attainable range for n_g, which may not be close enough to n_{SNV_2} to ensure this does not happen. To ensure that the back-transformation is meaningful, we handle these two cases by assigning to $z_{hap1,g}$ the values of $FT(0, n_g)$ and $FT(n_g, n_g)$, respectively. While the implementation of meta analysis in[24] follows the recommendation of[26] in using the harmonic mean of n_{SNV}'s as the 'average' total count n_g for backtransformation, we find that this gives too much weight to the loci with few read counts. Instead, we let n_g be a weighted arithmetic mean of n_{SNV}'s, with weights proportional to n_{SNV}'s and adding up to 1. In practice we find that the differences are negligible except for a few extreme cases of SNVs within the same gene that have drastically different coverage. The gene-level estimate of MAF is obtained as

$$T_{FT} = \hat{p}_{maj,g} = \max(\widehat{p}_{hap1,g}, 1 - \widehat{p}_{hap1,g}) \tag{20}$$

Since under the null hypothesis of no ASE, $Z_{hap1,g}$ follows a normal distribution with known mean and variance, a z-test can be employed to assess the significance of deviation from the null. A p-value can then be assigned to the observed extent of ASE. This is the p-value reported by MBASED when phasing information is available for the analyzed sample.

In practice, we do not have haplotype phasing information for samples in our study and MBASED was designed to be able to deal with such data sets. It circumvents the problem by phasing alleles at individual SNVs within a gene into haplotypes by assigning all major allele counts to one haplotype (which we will call haplotype 1) and all minor allele counts to another haplotype (haplotype 2), a so-called 'voting' approach to phasing. In other words, at each SNV we assign reference allele to haplotype 1 if

$$\frac{x_{ref,SNV}}{n_{SNV}} \geq 0.5 \tag{21}$$

and to haplotype 2 otherwise. Note that by design $\frac{x_{hap1,SNV}}{n_{SNV}} \geq 0.5$. The estimated haplotype 1 may not correspond to either of the two true underlying gene haplotypes. However, we expect that in cases of actual ASE ($p_{maj,g} >> 0.5$), our approach will in fact recover the true haplotypes, with haplotype 1 corresponding to the major of the two. We found this to be the case in our analysis of a sample with known haplotypes (secton 3). We also observed that in the simulations described in the main text, we successfully recovered true haplotypes for $> 99.5\%$ of ASE gene with ≥ 2 SNVs, when the MAF was set to 0.8, and $> 96\%$ of such genes when the MAF was set to 0.7 (data not shown). Even for a highly unfavorable case of small effect size ($MAF = 0.7$), large number of SNVs (4), and low coverage ($10 - 20$ reads/SNV), we recover more than 90% of true haplotypes.

We then proceed to apply the meta-analysis technique described above to haplotype 1 read

counts, and derive the gene-level estimate T_{FT} and the constancy statistic Q. However, the distributional assumptions on $Z_{hap1,g}$ under the null hypothesis of no ASE no longer hold, since, after phasing by MBASED, the observed counts $x_{hap1,SNV}$ are no longer the realizations of random variables

$$X_{hap1,SNV} \sim Bin(n = n_{SNV}, p = p_{hap1,g}) \tag{22}$$

Instead, they are realizations of random variables

$$X'_{hap1,SNV} = \max\{X_{hap1,SNV}, n_{SNV} - X_{hap1,SNV}\} \tag{23}$$

It is easy to see that using nominal p-values produced by applying meta-analysis to our pseudo-phased haplotypes will result in anti-conservative behavior of the testing procedure, as each individual SNV shows overexpression of the same haplotype. We estimate the true sampling distribution of T_{FT} by Monte Carlo simulations as follows. During each round of simulation, a 'reference' allele count $x_{sim,ref,SNV}$ is generated for each SNV in a gene from a $Bin(n = n_{SNV}, p = 0.5)$ distribution, and the 'alternative' allele count $x_{sim,alt,SNV}$ is set to $n_{SNV} - x_{sim,ref,SNV}$. The resulting allele counts are then phased into haplotype 1 ('major') and haplotype 2 ('minor') using the voting approach, and the meta-analysis MAF estimate $T_{sim,FT}$ is produced. Repeating this process N_{sim} times, an approximation to the null distribution of T_{FT} under the hypothesis of no ASE is generated. Finally, the ASE p-value is calculated as

$$p_{ASE} = \frac{\#(T_{sim,FT} \geq T_{FT})}{N_{sim}} \tag{24}$$

We note that simulations can also, in principle, be performed in the case of known true gene haplotypes. With large number of simulations, the resulting p-values will approximate the nominal meta-analysis p-values to an arbitrary degree of precision. This is the approach we adopted when comparing the performance of MBASED with and without information about true haplotypes (section 3), in order to obtain p-values with the same level of granularity. We observed that the correlation between the simulated (10^6 simulation rounds) and nominal p-values for multi-SNV genes was > 0.999 (data not shown).

Similarly, we approximate the null distribution of heterogeneity statistic Q by recording the observed values of Q during each simulation, and calculate the heterogenity p-value to be

$$p_{heterogeneity} = \frac{\#(Q_{sim} \geq Q)}{N_{sim}} \tag{25}$$

2.2.3 Single-SNV genes

For a single SNV under the conditions of no ASE, we have

$$X_{ref,SNV} \sim Bin(n = n_{SNV}, p = 0.5) \tag{26}$$

where $X_{ref,SNV}$ is the reference allele count. We use an approximation to a two-tailed binomial test to assess the extent of ASE in such genes. Formally, and analogously to the multi-SNV case, we take the observed major allele count x_{SNV} and transform it to $z_{SNV} = FT(x_{SNV}, n_{SNV})$, with the back-transformation $T_{FT} = FT^{-1}(z_{SNV}, n_{SNV}) = x_{SNV}/n_{SNV}$ yielding the estimate of MAF. In practice, for single-SNV genes, the transformation and back-transformation can be skipped.

Simulations are employed as for the multi-SNV case, the null distribution of T_{FT} is estimated, and the final p-value p_{ASE} is calculated as in (24). With large number of simulations, p_{ASE} will approximate the p-value from a two-tailed binomial exact test to an arbitrary degree of precision. The reason that we do not use the binomial test explicitly is our preference for a computationally uniform approach in both single- and multi-SNV scenarios.

2.2.4 Adjustment for pre-existing allelic bias

We assume once again that true phasing is known and extend model (8) to include situations of pre-existing allelic bias, by letting

$$X_{hap1,SNV} \sim Bin(n = n_{SNV}, p = f_{hap1,SNV}(p_{hap1,g})) \tag{27}$$

where we allow

$$f_{hap1,SNV}(p_{hap1,g}) \neq p_{hap1,g} \tag{28}$$

We further impose constraints (6) on $f_{hap1,SNV}(p_{hap1,g})$. We assume that $f_{hap1,SNV}(0.5)$ (probability of observing haplotype 1-supporting allele under conditions of no ASE) is known (or previously estimated by MBASED, see package vignette for details).

As an example of complications introduced by pre-existing allelic bias, consider the situation where reference bias exists at some SNV, such that probability of observing the reference allele under the conditions of no ASE is $p_{ref} = f_{ref,SNV}(p_{ref,g} = 0.5) = 0.7$. Then, for a total coverage of 20 reads, observing 14 reference-supporting reads should be considered the perfect case of no ASE ($14/20 = 0.7$, the expected fraction of reference reads under the no-ASE model), and having 12 reference-supporting reads would render the alternative allele 'major' and the reference allele 'minor' (since we observe fewer reference allele counts than we expect: $12/20 < 0.7$). Below we describe how the MBASED algorithm can incorporate information about pre-existing allelic bias into its computations.

At each SNV we employ the Freeman-Tukey transformation (10). Under the assumptions of model (27), the transformed values $z_{hap1,SNV}$ are realizations of independent (approximately) normal random varaibles $Z_{hap1,SNV}$, with

$$E(Z_{hap1,SNV}) = 2\sin^{-1}\left(\sqrt{f_{hap1,SNV}(p_{hap1,g})}\right) = 2\sin^{-1}\left(\sqrt{\frac{p_{hap1,g}}{p_{hap1,g} + C_{hap1,SNV} \times p_{hap2,g}}}\right) \tag{29}$$

where $C_{hap1,SNV} = \frac{f_{hap2,SNV}(0.5)}{f_{hap1,SNV}(0.5)}$, and the last equality is obtained from equation (6). In particular, under conditions of no ASE ($p_{hap1,g} = 0.5$), we have

$$E(Z_{hap1,SNV}) = 2\sin^{-1}\left(\sqrt{\frac{1}{1 + C_{hap1,SNV}}}\right) = 2\sin^{-1}\left(\sqrt{f_{hap1,SNV}(0.5)}\right) \tag{30}$$

Pre-existing allelic bias has no effect on the variance of $Z_{hap1,SNV}$ due to the variance-stabilizing property of Freeman-Tukey transformation, and we have

$$var(Z_{hap1,SNV}) = \frac{1}{n_{SNV} + 0.5}, \tag{31}$$

the same as in equation (12).

We need to modify our procedure for combining estimates of ASE at individual SNVs into a gene-level estimate to account for the fact that $E(Z_{hap1,SNV})$ may no longer be constant across SNVs within the same gene and is no longer equal to $2\sin^{-1}(\sqrt{p_{hap1,g}})$. Consider a shifting transformation

$$Z_{hap1,SNV,adj} = Z_{hap1,SNV} - 2\sin^{-1}\left(\sqrt{f_{hap1,SNV}(0.5)}\right) + 2\sin^{-1}\left(\sqrt{0.5}\right) \tag{32}$$

It follows that

$$\begin{aligned} E(Z_{hap1,SNV,adj}) &= E(Z_{hap1,SNV}) - 2\sin^{-1}\left(\sqrt{f_{hap1,SNV}(0.5)}\right) + 2\sin^{-1}\left(\sqrt{0.5}\right) \\ &= 2\sin^{-1}\left(\sqrt{f_{hap1,SNV}(p_{hap1,g})}\right) - 2\sin^{-1}\left(\sqrt{f_{hap1,SNV}(0.5)}\right) + 2\sin^{-1}\left(\sqrt{0.5}\right) \end{aligned} \tag{33}$$

If $p_{hap1,g} \approx 0.5$, then

$$E(Z_{hap1,SNV,adj}) \approx 2\sin^{-1}(\sqrt{0.5}) \approx 2\sin^{-1}(\sqrt{p_{hap1,g}}) \tag{34}$$

and we are once again within the framework described in section 2.2.2. Note that the shifting transformation (32) has no effect on variance: $var(Z_{hap1,SNV,adj}) = var(Z_{hap1,SNV})$.

To see how successful this shifting transformation is in bringing the means of $Z_{hap1,SNV}$'s close to $2\sin^{-1}(\sqrt{p_{hap1,g}})$ when $p_{hap1,g} \neq 0.5$, we assessed the performance of the shift across a range of values of $p_{hap1,g}$ and $f_{hap1,SNV}(0.5)$ (Figures S16 and S17). We find that the performance is remarkably good, especially for mild values of allelic bias $0.4 \leq f_{hap1,SNV}(0.5) \leq 0.6$. We conclude that

$$E(Z_{hap1,SNV,adj}) \approx 2\sin^{-1}(\sqrt{p_{hap1,g}}) \tag{35}$$

The pre-existing allelic bias adjustment is incorporated into a 1-sample MBASED analysis in the following ways:

1. Haplotype phasing (if true haplotypes are unknown) : At each SNV we assign reference allele to haplotype 1 ('major') if

$$FT^{-1}(z_{ref,SNV,adj}, n_{SNV}) \geq 0.5 \tag{36}$$

   and to haplotype 2 ('minor') otherwise. If $f_{ref,SNV}(0.5) = 0.5$ (no pre-existing allelic bias), this is the same condition as before, since the expression (36) reduces to $\frac{x_{ref,SNV}}{n_{SNV}} \geq 0.5$. In all cases, the condition is essentially checking whether the observed reference allele count is larger or smaller than expected under conditions of no ASE.

2. Meta-analysis: Instead of using SNV-level values $z_{hap1,SNV}$ (10) for transformed haplotype 1 allele counts, we use the adjusted versions $z_{hap1,SNV,adj}$ (32).

3. Simulations (if true haplotypes are unknown): Instead of simulating $X_{sim,ref,SNV}$ at each locus from a $Bin(n_{SNV}, p = 0.5)$ distribution, we use a $Bin(n_{SNV}, p = f_{ref,SNV}(0.5))$ distribution.

2.2.5 Estimating $f_{hap1,SNV}(0.5)$

By default, MBASED assumes that at each locus $f_{ref,SNV}(0.5) = f_{alt,SNV}(0.5) = 0.5$, i.e., that there is no pre-existing allelic bias. The user can supply the value of $f_{ref,SNV}(0.5)$ for each locus, or supply a list of read counts from which this value should be estimated (see package vignette for more details). In our analysis, we assumed that $f_{ref,SNV}(0.5)$ is constant across all SNVs within a sample (global reference bias), and we estimated this value as

$$N_{ref}/N_{total} \tag{37}$$

which is the ratio of all reference reads to total reads in the sample, after excluding the loci with the top 5% of read counts (trimmed for robustness). Note that our estimate of $f_{ref,SNV}(0.5)$ is sample-specfic. We find that across 32 samples, the no-ASE reference probability $f_{ref,SNV}(0.5)$ ranges from 0.50 to 0.55 with a median of 0.52. In other words, we observe mild levels of pre-existing allelic bias that favors reference allele.

In cases where we merged multiple SNVs into a single locus because they were covered largely by the same set of reads, we used a slightly more complicated procedure to obtain estimate of pre-existing allelic bias. A quick example will suffice to illustrate our approach. Consider a locus with 2 SNVs. Since the SNVs are overlapped by the same reads, we can resolve them unambigously into 2 haplotypes. Suppose, that the two resolved haplotypes at this locus are $R_1 = \{ref, ref\}$ and $R_2 = \{alt, alt\}$, and suppose that the allele R_1 belongs to haplotype 1 for gene g. Let p_{ref} denote the constant $f_{ref,SNV}(0.5)$ we estimated for the sample in hand. Then we model the probability of observing a read supporting haplotype 1 at this locus under the null hypothesis of no ASE as

$$f_{hap1,locus}(0.5) = \frac{p_{ref} \times p_{ref}}{p_{ref} \times p_{ref} + p_{alt} \times p_{alt}} \tag{38}$$

Similarly, if the two locus haplotypes are $R_1 = \{ref, alt\}$ and $R_2 = \{alt, ref\}$, we have

$$f_{hap1,locus}(0.5) = \frac{p_{ref} \times p_{alt}}{p_{ref} \times p_{alt} + p_{alt} \times p_{ref}} = 0.5 \tag{39}$$

We then let $p_{hap_2} = 1 - p_{hap_1}$. The reasoning used here is entirely analogous to that used to define proportionality conditions (6), with overall (locus) allele probability under conditions of no ASE proportional to allele probabilities at each individual SNV. We then treat the locus as a single SNV, with allelic probabilities under conditions of no ASE given by $f_{R_1,SNV}(0.5)$ and $f_{R_2,SNV}(0.5)$, and use read counts supporting R_1 and R_2 in our calculations, analogously to using read counts supporting reference and alternative alleles in the case of a locus with a single SNV. We stress that this calculation is not part of the MBASED algorithm, but is a feature of our preprocessing of the data prior to running MBASED.

2.2.6 Adjustment for technical overdispersion

We now extend the binomial model (27) from the preceding section to the beta-binomial model (4) used by MBASED, where we allow

$$\rho_{SNV} \neq 0 \tag{40}$$

We assume that true phasing is known and that the value of ρ_{SNV} for each SNV is known (or previously estimated by MBASED, see package vignette for details).

Consider SNV-level FT-values $Z_{hap1,SNV,adj}$, defined in (32). While switching from binomial to beta-binomial distributional assumptions on $X_{hap1,SNV}$ may prevent $Z_{hap1,SNV,adj}$ from following (approximately) the normal distribution, we note that the assumption of normality is never explicitly employed by MBASED. Instead, the empirical distribution of the test statistic T_{FT} is estimated in order to assign statistical significance to the observed ASE. Therefore, our concern is only with the effects of overdispersion on the mean and variance of $Z_{hap1,SNV,adj}$.

Let

$$Y \sim Beta(\mu = f_{hap1,SNV}(p_{hap1,g}), \rho = \rho_{SNV}) \tag{41}$$

Then

$$X_{hap1,SNV} \sim Bin(n = n_{SNV}, p = Y) \tag{42}$$

is another parametrization of (4). Note that $E(Y) = \mu$ and $var(Y) = \rho \times \mu \times (1 - \mu)$.

Now, consider

$$E(Z_{hap1,SNV,adj}) = E(Z_{hap1,SNV} - 2\sin^{-1}\left(\sqrt{f_{hap1,SNV}(0.5)}\right) + 2\sin^{-1}(\sqrt{0.5})) \tag{43}$$

Both $2\sin^{-1}\left(\sqrt{f_{hap1,SNV}(0.5)}\right)$ and $2\sin^{-1}(\sqrt{0.5})$ are constants. Furthermore, by law of total expectation:

$$E(Z_{hap1,SNV}) = E(E(Z_{hap1,SNV}|Y)) = E(2\sin^{-1}(\sqrt{Y})) \tag{44}$$

Using Taylor series expansion for $f(Y) = 2\sin^{-1}(\sqrt{Y})$ around $E(Y) = \mu$, we have

$$\begin{aligned} E(2\sin^{-1}(\sqrt{Y})) &\approx E\left(2\sin^{-1}(\sqrt{\mu}) + (Y-\mu)(\mu-\mu^2)^{-\frac{1}{2}} + \frac{1}{2}(Y-\mu)^2\frac{2\mu-1}{2(\mu-\mu^2)^{\frac{3}{2}}}\right) \\ &= 2\sin^{-1}(\sqrt{\mu}) + \frac{1}{4}\rho\frac{2\mu-1}{\sqrt{\mu-\mu^2}} \end{aligned} \tag{45}$$

Taking the ratio of the second term in the expansion to the first term, we observe that

$$\frac{\|\frac{1}{4}\rho\frac{2\mu-1}{\sqrt{\mu-\mu^2}}\|}{2\sin^{-1}(\sqrt{\mu})} \leq 0.05 \tag{46}$$

when $0 \leq \rho \leq 0.02$, $0.2 \leq f_{hap1,SNV}(0.5) \leq 0.8$, and $0.1 \leq p_{hap1,g} \leq 0.9$. We believe these values to encompass the range of overdispersion and pre-existing allelic bias commonly observed in real data. For example, we estimate $\rho \leq 0.01$ in all of our normal data sets (see discussion below), while other authors report overdispersion several orders of magnitude lower[23]. Therefore

$$E(2\sin^{-1}(\sqrt{Y})) \approx 2\sin^{-1}(\sqrt{\mu}) \tag{47}$$

and from (43):

$$E(Z_{hap1,SNV,adj}) \approx 2\sin^{-1}(\sqrt{\mu}) - 2\sin^{-1}(\sqrt{f_{hap1,SNV}(0.5)}) + 2\sin^{-1}(\sqrt{0.5}) \tag{48}$$

Plugging in the value of $\mu = f_{hap1,SNV}(p_{hap1,g})$, we observe that this is the same expression as (33), and therefore $E(Z_{hap1,SNV,adj})$ is unaffected by the presence of overdispersion. Just as in

section 2.2.4, we performed simulations across a range of values of ρ, $f_{SNV,hap1}(0.5)$ and $p_{hap1,g}$ to assess how good the shifting transformation (32) is at bringing the means of $Z_{hap1,SNV}$'s close to $2\sin^{-1}(\sqrt{p_{hap1,g}})$. We find that even for very large values of ρ ($\rho = 0.1$), the approximation performs similarly to the case of no overdispersion (data not shown).

Now, consider

$$var(Z_{hap1,SNV,adj}) = E(var(Z_{hap1,SNV,adj}|Y)) + var(E(Z_{hap1,SNV,adj}|Y)) \tag{49}$$

We have

$$E(var(Z_{hap1,SNV,adj}|Y)) = E\left(\frac{1}{n_{SNV} + 0.5}\right) = \frac{1}{n_{SNV} + 0.5} \tag{50}$$

since, due to the variance-stabilizing property of Freeman-Tukey transformation, $var(Z_{hap1,SNV,adj})$ does not depend on Y. Further, using the first two terms from the Taylor series expansion employed in (45), we get

$$var(E(Z_{hap1,SNV,adj}|Y)) \approx \frac{var(Y)}{\mu - \mu^2} = \rho \tag{51}$$

Therefore,

$$var(Z_{hap1,SNV,adj}) \approx \frac{1}{n_{SNV} + 0.5} + \rho \tag{52}$$

Note that it is identical to the previous formula for $var(Z_{hap1,SNV,adj})$ when $\rho = 0$. Simulations across a range of values of ρ, $f_{SNV,hap1}(0.5)$ and $p_{hap1,g}$ showed approximation (52) to be quite excellent at moderate values of overdispersion ($\rho < 0.02$, data not shown).

MBASED adjusts for technical overdispersion in the following ways:

1. Meta-analysis: we use the new variance estimates (52) when calculating weights (14) used to derive $z_{hap1,g}$

2. Simulations (when true haplotypes are uknown): We simulate $X_{sim,ref,SNV}$ at each locus from a

$$BetaBin(n = n_{SNV}, \mu = f_{ref,SNV}(0.5), \rho = \rho_{SNV})) \tag{53}$$

distribution instead of from a

$$Bin(n = n_{SNV}, p = f_{ref,SNV}(0.5)) \tag{54}$$

distribution.

2.2.7 Estimating ρ_{SNV}

By default, MBASED assumes that at each locus $\rho_{SNV} = 0$, i.e., that there is no overdispersion due to technical effects. The user can supply the value of ρ_{SNV} for each locus, or supply a list of read counts from which this value should be estimated (see package vignette for more details). In our analysis, we estimated ρ_{SNV} within 7 normal samples only, since large amount of copy number alterations in tumor samples will lead to very high estimates of overdispersion in the data. We assumed that ρ_{SNV} is constant across all SNVs within a sample (global overdispersion effect), and we estimated this value by maximum likelihood within each sample. At each SNV, we found the

likelihood for the observed number of reference allele-supporting reads under the

$$BetaBin(n = n_{SNV}, \mu = f_{ref,SNV}(0.5), \rho) \tag{55}$$

distribution, where we used a previously obtained value $f_{ref,SNV}(0.5) = p_{ref}$ of global reference bias (see section 2.2.5). The value of ρ maximizing the joint likelihood over all SNVs was then used as an estimate of ρ_{SNV} at all SNVs in that sample. The estimation procedure only included SNVs that were not parts of larger merged loci. To eliminate potential artifacts that may affect the model fit, we further required that the SNV be detected in at least 2 samples, with at least 1 sample presenting the reference allele as major and at least 1 sample presenting the alternative allele as major. Finally, after the initial model fit, we calculated the model-based p-value at each SNV, removed all SNVs with Benjamini-Hochberg adjusted p-values ≤ 0.05 (outliers), and re-fit the model to obtain the final estimate. We found that a small number of SNVs get removed by the outlier filtering ($\leq 1\%$ in each sample), and that no SNVs showed small adjusted p-values after the second fit. We found this step of outlier removal quite useful, as discaring one or two SNVs often led to two-fold decrease in overdispersion estimate. Moreover, since the decision to remove an SNV was based on adjusted p-value, we strongly feel that the final estimates represent the appropriate model parameters for all but a hanful of SNVs in the data. We found that for 5 out of 7 normal samples, overdispersion estimates $\widehat{\rho}$ are between 0.001 and 0.005, with the other 2 exhibiting extreme values of 0.0000005 and 0.01. The median $\widehat{\rho}$ across all 7 normal samples was 0.004 and this is the value we used for all samples (both tumor and normal) in the current study.

2.3 2-Sample Analysis

In some situations it may be desirable to compare allelic expression between two samples. One scenario for this situation is comparing tumor and normal samples from the same individual to detect tumor-specific ASE or loss of imprinting (normal-specific ASE). Another scenario is comparing RNA counts to those form genomic DNA to identify instances of ASE which are not associated with underlying copy number imbalance. In what follows, we will let sample 1 stand for 'tumor' and sample 2 stand for 'normal'. Note that the presented approach is *not* symmetrical with respect to the two samples, and care should be taken in deciding which sample to designate as either sample 1 or sample 2. We assume that there are exactly 2 distinct haplotypes for each tested gene, that the haplotypes are the same between the two samples, and that for gene g and sample $s \in \{sample1, sample2\}$, the observed number of reads supporting haplotype 1 at an exonic heterozygous SNV $X_{hap1,SNV,s}$ follows the beta-binomial model (4), which we reproduce here:

$$X_{hap1,SNV,s} \sim BetaBin(n = n_{SNV,s}, \mu = f_{hap1,SNV,s}(p_{hap1,g,s}), \rho = \rho_{SNV,s}) \tag{56}$$

Note that model parameters are sample-specific. We denote the observed counts in sample 1 and sample 2 by $x_{hap1,SNV,sample1}$ and $x_{hap1,SNV,sample2}$, respectively. As with the 1-sample MBASED algorithm, we assume that SNV-level counts within a gene are independent, and we further assume independence of SNV-level counts between the two samples. We choose the haplotype frequency difference for some choice of haplotype 1, defined as

$$D = p_{hap1,g,sample1} - p_{hap1,g,sample2} \tag{57}$$

as our measure of sample-specific ASE (or between-sample allelic imbalance). This is known as 'excess risk' in the epidemiology literature. We are interested in testing the null hypothesis of no sample-specific ASE:

$$H_0 : D = 0 \tag{58}$$

or, equivalently,

$$H_0 : p_{hap1,g,sample1} = p_{hap1,g,sample2} = p_{hap1,g} \tag{59}$$

Note that we do not require that $p_{hap1,g} = 0.5$ in the null hypothesis (59), since in the 2-sample analysis we are only interested in the genes with sample-specific ASE, rather than all genes exhibiting ASE, which may include instances of ASE common to both samples.

As with 1-sample analysis, we will first describe the algorithm under the assumption of binomial read counts ($\rho_{SNV,s} = 0$ for both samples) with no pre-existing allelic bias ($f_{SNV}(p_{hap1,g,s}) = p_{hap1,g,s}$ for both samples), reducing the model to

$$X_{hap1,SNV,s} \sim Bin(n = n_{SNV,s}, p = p_{hap1,g,s}) \tag{60}$$

We then demonstrate the extensions to take pre-existing allelic bias into account ($f_{SNV}(p_{hap1,g,s}) \neq p_{hap1,g,s}$, section 2.3.4) and to include overdispersion ($\rho_{SNV,s} \neq 0$, section 2.3.5).

2.3.1 Multi-SNV genes

Consider a gene with several heterozygous exonic SNVs, and suppose that phasing information is available, i.e., for each SNV we know which allele belongs to haplotype 1 and which to haplotype 2. Meta-analysis techinques can be employed to test a multi-SNV gene for allelic imbalance between the two samples in a manner analogous to that in the 1-sample case. The only difference between the two cases is the origin and interpretation of (approximately) normal variables $Z_{hap1,SNV}$ that are combined.

Consider the following SNV-level estimate of D:

$$Z_{hap1,SNV} = P_{hap1,SNV,sample1} - P_{hap1,SNV,sample2} \tag{61}$$

where

$$P_{hap1,SNV,s} = \frac{X_{hap1,SNV,s}}{n_{SNV,s}} \tag{62}$$

for $s \in \{sample1, sample2\}$. This estimate is simply a difference in proportions of haplotype 1 allele-supporting reads between the two samples at that SNV, and we will call $Z_{hap1,SNV}$ the PD-value (for 'Proporition Difference') and denote its observed value by $z_{hap1,SNV}$. We further denote the observed proportions of haplotype 1 allele-supporting reads by $p_{hap1,SNV,sample1}$ and $p_{hap1,SNV,sample2}$. Note the difference between the observed proportion of haplotype 1 allele-supporting reads $p_{hap1,SNV,s}$ and the true (unkown) underlying frequency of haplotype 1 transcripts $p_{hap1,g,s}$.

Under the assumptions of binomial model (60), we have

$$E(P_{hap1,SNV,s}) = p_{hap1,g,s} \tag{63}$$

and

$$var(P_{hap1,SNV,s}) = \frac{p_{hap1,g,s} \times (1 - p_{hap1,g,s})}{n_{SNV,s}} \tag{64}$$

Furthermore, $Z_{hap1,SNV}$ is an approximately normal random variable with

$$E(Z_{hap1,SNV}) = p_{hap1,g,sample1} - p_{hap1,g,sample2} = D \tag{65}$$

and

$$var(Z_{hap1,SNV}) = \frac{p_{hap1,g,sample1}(1 - p_{hap1,g,sample1})}{n_{SNV,sample1}} + \frac{p_{hap1,g,sample2}(1 - p_{hap1,g,sample2})}{n_{SNV,sample2}} \tag{66}$$

Since the true values $p_{hap1,g,sample1}$ and $p_{hap1,g,sample2}$ are unknown, we estimate the variance of $Z_{hap1,SNV}$ as

$$\begin{aligned}\widehat{var}(Z_{hap1,SNV}) = &\frac{p'_{hap1,SNV,sample1}(1 - p'_{hap1,SNV,sample1})}{n_{SNV,sample1} + 1} \\ &+ \frac{p'_{hap1,SNV,sample2}(1 - p'_{hap1,SNV,sample2})}{n_{SNV,sample2} + 1}\end{aligned} \tag{67}$$

where for $s \in \{sample1, sample2\}$

$$p'_{hap1,SNV,s} = \frac{x_{hap1,SNV,s} + 0.5}{n_{SNV,s} + 1} \tag{68}$$

is the attenuated observed proportion of haplotype 1 allele-supporting reads. Note that the variance estimate (67) results from adding a pseudocount of 0.5 to each allele in both samples. This is done to avoid the situations where the estimated variance is 0 (e.g., if observed haplotype 1 proportions in both samples are 1). We do not attenuate the observed proportions when calculating $z_{hap1,SNV}$ in equation (61).

Following the meta-analysis procedure described in section 2.2.2, a gene level PD-value $z_{hap1,g}$ is computed as the weighted mean of the SNV-level PD-values $z_{hap1,SNV}$, with the weights given by the inverse of the estimated variances of $Z_{hap1,SNV}$'s and normalized to add up to 1. We let $Z_{hap1,g}$ denote the random variable corresponding to the gene-level PD-value. The observed gene-level PD-value $z_{hap1,g}$ is the estimate of the haplotype 1 allele frequency difference between the two samples,

$$T_{PD} = \widehat{D} = z_{hap1,g} \tag{69}$$

Analogously to the 1-sample case, DerSimonian-Laird constancy statistic Q is calculated and the corresponding heterogeneity (inter-loci variability) p-value is recorded, with small p-values indicating evidence for isoform-specific between-sample allelic imbalance or other source of excessive variability. Same considerations as in 1-sample case apply to interpretation of heterogeneity p-values and to the power of statistical tests based on Q.

We note that unlike the 1-sample approach presented earlier, the SNV-specific measures of ASE used in the 2-sample algorithm are not variance-stabilized. This leads to a certain bias in the calculation of $z_{hap1,g}$, as some extreme values of $z_{hap1,SNV}$, e.g., those close to 1, corresponding to

$$p'_{hap1,SNV,sample1} \approx 1$$

and

$$p'_{hap1,SNV,sample2} \approx 0$$

will be assigned greater weight due to the small values of their estimated variances (given in (66)). While variance-stabilizing transformations have been proposed for differences of binomial proportions [27], their variance-stabilizing properties only hold when the true value of D is close to that specified by a null hypothesis of interest (e.g., $D = 0$). We could have adopted this approach for the purpose of obtaining a measure of statistical significance of observed deviations from the null, but there is no straightforward way to simultaneously obtain the estimate of the observed ASE that is not computationally determined by the null hypothesis. We plan to address this issue in a future release of MBASED.

When phasing information is unavailable, MBASED modifies the meta-analysis approach described above in the 2-sample setting as follows. As a first step, SNV-level counts within each gene are phased into haplotypes in the manner identical to that of the 1-sample analysis, with one caveat: phasing of both sample 1 and sample 2 is based solely on allelic counts from sample 1. In other words, all alleles presenting as major in sample 1 are assigned to the same haplotype (which we denote as 'haplotype 1' by analogy with 1-sample case, see section 2.2.2). This introduces a certain asymmetry into the analysis, but ensures that both samples have the same pair of haplotypes. The choice of sample 1 instead of sample 2 for this phasing step is motivated by the fact that it is the ASE in sample 1 which is of interest, and that for genes showing true sample 1-specific ASE, the allele counts in sample 1 are more informative for haplotype reconstruction. Additionally, 'voting'-based pseudo-phasing of SNVs into haplotypes based on both samples could be highly misleading in cases where the direction of within-sample ASE changes between the two samples. For example, if the ratio of the two haplotypes is 0.7/0.3 in the normal sample and 0.3/0.7 in the tumor sample, then the 'voting'-based pseudo-phasing of SNVs into 'major' and 'minor' haplotypes based on read counts in both samples is likely to result in chimeric haplotypes, when phasing based on any one single sample would likely recover the true underlying haplotypes.

After 'voting'-based pseudo-phasing is performed, we obtain a gene-level estimate of the between-sample allelic imbalance T_{PD} and the heterogeneity statistic Q (with corresponding p-value), as described above. We then estimate the null distribution of T_{PD} for each gene using simulations, analogous to 1-sample case, and assign a final ASE-pvalue p_{ASE} as follows.

Recall that under the null hypothesis of no sample-specific ASE (58), we have

$$p_{hap1,g,sample1} = p_{hap1,g,sample2} = p_{hap1,g} \tag{70}$$

where $hap1$ and $hap2$ refer to true underlying haplotypes (and not to haplotypes 1 and 2 obtained based on the 'voting' pseudo-phasing of sample 1 allele read counts). Also, under the null hypothesis, let us denote

$$p_{maj,g} = \max\{p_{hap1,g}, p_{hap2,g}\} \tag{71}$$

In each round of simulations, one allele at each SNV is randomly assigned to major haplotype, and the simulated major haplotype counts $x_{sim,maj,SNV,sample1}$ and $x_{sim,maj,SNV,sample2}$ are drawn independently from

$$Bin(n = n_{SNV,sample1}, p = \widehat{p}_{maj,g}) \tag{72}$$

and

$$Bin(n = n_{SNV,sample2}, p = \widehat{p}_{maj,g}) \tag{73}$$

distributions, respectively (we address estimation of $\widehat{p}_{maj,g}$ in section 2.3.2). The SNVs are then phased into haplotypes 1 and 2 based on the 'voting' algorithm applied to the simulated counts in

sample 1, and the proportion difference $T_{sim,PD}$ is calculated. The final ASE p-value is derived as

$$p_{ASE} = \frac{\#(T_{sim,PD} \geq T_{PD})}{N_{sim}} \tag{74}$$

where N_{sim} is the number of simulations. Note that the empirical significance test (74) is one-sided. The reason for this is as follows. We expect that in cases of true strong sample 1-specific ASE, our 'voting' pseudo-phasing procedure will recover true underlying haplotypes, with haplotype 1 being the major of the two. This in turn results in $p_{hap1,g,sample1} = p_{maj,g,sample1}$ in equation (57), producing a large positive value of $\widehat{D} = T_{PD}$, unless the ASE is not specific to sample 1 and $p_{maj,g,sample2}$ is also large.

In principle, the simulations can also be employed when dealing with paired samples where the true haplotypes are known, just as in 1-sample analysis. Finally, we note that the heterogeneity p-value is calculated based on simulations, in a manner identical to the 1-sample analysis (equation 25

2.3.2 Estimation of $p_{maj,g}$

There is an additional layer of complexity involved in the 2-sample analysis. The null hypothesis of no sample-specific ASE (59) does not specify the exact value of $p_{hap1,g}$. If true phasing is known, we can obtain maximum likelihood estimate $\widehat{p}_{hap1,g}$ under the null as follows. For a given value of $p_{hap1,g}$ the likelihood of the observed data at an individual SNV under the null hypothesis (59) and data-generating binomial model (60) is

$$L_{SNV}(p_{hap1,g}) = \prod_{s \in \{sample1, sample2\}} \binom{n_{SNV,s}}{x_{hap1,SNV,s}} p_{hap1,g}^{x_{hap1,SNV,s}} p_{hap2,g}^{x_{hap2,SNV,s}} \tag{75}$$

where

$$p_{hap2,g} = 1 - p_{hap1,g} \tag{76}$$

and

$$x_{hap2,SNV,s} = n_{SNV,s} - x_{hap1,SNV,s} \tag{77}$$

The joint likelihood across all SNVs in gene g is given by

$$L(p_{hap1,g}) = \prod_{SNV_i} L_{SNV_i}(p_{hap1,g}) \tag{78}$$

with $\widehat{p}_{hap1,g}$ being the value of $p_{hap1,g}$ which maximizes the joint likelihood (78). We can then obtain estimate

$$\widehat{p}_{maj,g} = \max\{\widehat{p}_{hap1,g}, 1 - \widehat{p}_{hap1,g}\} \tag{79}$$

When phasing is unknown, this procedure needs to be modified. One solution would be to use the haplotypes produced by MBASED using 'voting'-based pseudo-phasing approach on sample 1 read counts, since these are the haplotypes used to obtain $\widehat{D} = T_{PD}$ (see above). However, this approach tends to consistently overestimate $p_{maj,g}$ under the null hypothesis for the following reason. Our pseudo-phasing algorithm results in haplotype 1 such that $\frac{x_{haplotype1,SNV,sample1}}{n_{SNV,sample1}} \geq 0.5$ at each SNV, producing strong evidence for $p_{haplotype1,g} > 0.5$. Even when information from the

second sample is taken into account in the likelihood expression (75), the end result of maximizing the likelihood, in general, will be identification of haplotype 1 as 'major'. While this poses no problem when haplotype 1 does in fact correspond to the major haplotype of the gene (as is likely to be the case when sample 1 exhibits strong ASE, whether sample-specific or not), it will artificially inflate $\widehat{p}_{maj,g} = \widehat{p}_{haplotype1,g}$ otherwise. Therefore, we adopt a different strategy and estimate $p_{maj,g}$ under the null hypothesis by maximizing over all possible assignments of SNV-level alleles to gene haplotypes. In other words, if a gene contains 3 heterozygous SNVs, we maximize likelihood over all $2^3 = 8$ possible haplotype reconstructions. We describe this procedure in detail below. For clarity of notation, we denote two arbitrary haplotypes of a gene as $hapA$ and $hapB$, to distinguish them from previously used $hap1$ and $hap2$.

We re-write the likelihood (75) of the observed data at an invididual SNV under the null hypothesis for a given haplotype phasing into $hapA$ and $hapB$ as

$$\begin{aligned} L_{SNV}(p_{hapA,g}|hapA) = I_{SNV,ref=hapA} & \prod_{s\in\{sample1,sample2\}} \binom{n_{SNV,s}}{x_{ref,SNV,s}} p_{hapA,g}^{x_{ref,SNV,s}} p_{hapB,g}^{x_{alt,SNV,s}} \\ + (1 - I_{SNV,ref=hapA}) & \prod_{s\in\{sample1,sample2\}} \binom{n_{SNV,s}}{x_{ref,SNV,s}} p_{hapB,g}^{x_{ref,SNV,s}} p_{hapA,g}^{x_{alt,SNV,s}} \end{aligned} \tag{80}$$

where the indicator variable $I_{SNV,ref=hapA} \in \{0,1\}$ is used to denote whether the reference allele belongs to $hapA$ (1) or $hapB$ (0), and $p_{hapB,g} = 1 - p_{hapA,g}$. Thus, for each of the 2 possible haplotype assignments of the reference allele, only 1 of the 2 summands in expression (80) is non-zero.

The joint likelihood across all SNVs within a particular gene can be written as

$$L(p_{hapA,g}|hapA) = \prod_{SNV_i} L_{SNV_i}(p_{hapA,g}|hapA) \tag{81}$$

For each choice of haplotype assignments $hapA$ and $hapB$, we let $\widehat{p}_{hapA,g}$ be the value of $p_{hapA,g}$ that maximizes the joint likelihood (81), with the corresponding likelihood value $L(\widehat{p}_{hapA,g}|hapA)$. Finally, among all haplotype assignment-specific likelihood values $L(\widehat{p}_{hapA,g}|hapA)$, we choose the maximum one, and denote the corresponding $\widehat{p}_{hapA,g}$ by $\widehat{p}_{hapA^*,g}$. We then let

$$\widehat{p}_{maj,g} = \max\{\widehat{p}_{hapA^*,g}, 1 - \widehat{p}_{hapA^*,g}\} \tag{82}$$

We note that the obtained estimate is biased in cases where true $p_{maj,g} \approx 0.5$, and this may lead to some anti-conservative p-value calls on the part of MBASED. In particular, we may observe enrichment for low p-values if the estimated $widehatp_{maj,g}$ is sufficiently greater than $p_{maj,g} = 0.5$. We found, however, that we do not observe such enrichment under the levels of dispersion seen in our data, although we do see occasional instances of this if we assume binomial (no dispersion) model (data not shown). This limitation should be addressed in future release of MBASED.

2.3.3 Single-SNV genes

The 2-sample analysis for single-SNV genes is identical to that for multi-SNV genes (including simulations), except that the gene-level PD-value $T_{PD} = z_{hap1,g}$ is the SNV-level PD-value $z_{hap1,SNV}$.

2.3.4 Adjustment for pre-existing allelic bias

We extend model (60) to include situations of pre-existing allelic bias by letting

$$X_{hap1,SNV,s} \sim Bin(n = n_{SNV,s}, p = f_{hap1,SNV,s}(p_{hap1,g,s})) \tag{83}$$

for $s \in \{sample1, sample2\}$, where we allow

$$f_{hap1,SNV,s}(p_{hap1,g,s}) \neq p_{hap1,g,s} \tag{84}$$

for at least one sample. We still assume functional form (6) for $f_{hap1,SNV,s}(p_{hap1,g,s})$. Note that we do not assume that pre-existing allelic bias is the same for both samples, i.e., we allow

$$f_{hap1,SNV,sample1}(0.5) \neq f_{hap1,SNV,sample2}(0.5) \tag{85}$$

Under the assumptions of model (83), we obtain from (62) that

$$E(P_{hap1,SNV,s}) = f_{hap1,SNV,s}(p_{hap1,g,s}) \tag{86}$$

and

$$var(P_{hap1,SNV,s}) = \frac{f_{hap1,SNV,s}(p_{hap1,g,s}) \times (1 - f_{hap1,SNV,s}(p_{hap1,g,s}))}{n_{SNV,s}} \tag{87}$$

It follows that

$$E(Z_{hap1,SNV}) = f_{hap1,SNV,sample1}(p_{hap1,g,sample1}) - f_{hap1,SNV,sample2}(p_{hap1,g,sample2}) \tag{88}$$

Comparing to equation (57), we note that in general

$$E(Z_{hap1,SNV}) \neq D \tag{89}$$

unless there is no pre-existing allelic bias ($f_{hap1,SNV,s}(p_{hap1,g,s}) = p_{hap1,g,s}$) for both samples. Of course, we might still have $E(Z_{hap1,SNV}) = D$ for some specially chosen values of $p_{hap1,g,s}$ and $f_{hap1,SNV,s}(0.5)$.

We need to modify our procedure for combining estimates of ASE at individual SNVs into a gene-level estimate to account for the fact that $E(Z_{hap1,SNV})$ may no longer be constant across SNVs within the same gene and is no longer equal to D. Consider a transformation based on our approach to handling pre-existing allelic bias in the 1-sample analysis (section 2.2.4):

$$Y_{hap1,SNV,s} = FT\left(X_{hap1,SNV,s}, n_{SNV,s}\right) - 2\sin^{-1}\left(\sqrt{f_{hap1,SNV,s}(0.5)}\right) + 2\sin^{-1}\left(\sqrt{0.5}\right)$$

$$P_{hap1,SNV,s,adj} = FT^{-1}\left(Y_{hap1,SNV,s}, n_{SNV,s}\right)$$

$$Z_{hap1,SNV,adj} = P_{hap1,SNV,sample1,adj} - P_{hap1,SNV,sample2,adj} \tag{90}$$

where $s \in \{sample1, sample2\}$. In words, we use Freeman-Tukey transformation on observed haplotype 1 allele-supporting counts in each sample and adjust the resulting FT-value in the same manner as we did in 1-sample case to obtain $Y_{hap1,SNV,s}$. We then backtransform the shifted FT-value into a shifted proportion ($P_{hap1,SNV,s,adj}$) and let $Z_{hap1,SNV,adj}$ be the difference be-

tween the shifted proportions in samples 1 and 2. We denote the observed values of random variables $P_{hap1,SNV,sample1,adj}$, $P_{hap1,SNV,sample2,adj}$, and $Z_{hap1,SNV,adj}$ by $p_{hap1,SNV,sample1,adj}$, $p_{hap1,SNV,sample2,adj}$, and $z_{hap1,SNV,adj}$, respectively. Note, that transformation (90) yields previously described model (61) in the case of no pre-existing allelic bias ($f_{hap1,SNV}(0.5) = 0.5$), since in such case we have

$$Y_{hap1,SNV,s} = FT\left(X_{hap1,SNV,s}, n_{SNV,s}\right)$$

$$P_{hap1,SNV,s,adj} = FT^{-1}\left(Y_{hap1,SNV,s}, n_{SNV,s}\right) = \frac{X_{hap1,SNV,s}}{n_{SNV,s}} = P_{hap1,SNV,s}$$

$$Z_{hap1,SNV,adj} = P_{hap1,SNV,sample1,adj} - P_{hap1,SNV,sample2,adj} = Z_{hap1,SNV} \tag{91}$$

The goal of transformation (90) is to produce shifted proportion $P_{hap1,SNV,s,adj}$, such that

$$E(P_{hap1,SNV,s,adj}) \approx p_{hap1,g,s} \tag{92}$$

We find that approximation (92) holds for a wide range of values of $p_{hap1,g,s}$ and $f_{hap1,SNV,s}(0.5)$, with slightly worse performance for extreme values of pre-existing allelic bias (Figure S17).

To address the issue of variance of $P_{hap1,SNV,s,adj}$, we suggest the following approximation:

$$var(P_{hap1,SNV,s,adj}) \approx \frac{E(P_{hap1,SNV,s,adj})(1 - E(P_{hap1,SNV,s,adj}))}{n_{SNV,s}} \tag{93}$$

We found this approximation to perform well in general, and especially so for mild values of pre-existing allelic bias ($0.4 \leq f_{hap1,SNV,s}(0.5) \leq 0.6$) and for moderate-to-high coverages ($n_{SNV,s} \geq 20$) (Figure S18). We plan to address any remaining inadequacies in a future release of MBASED.

Based on approximations (92) and (93), we conclude that

$$E(Z_{hap1,SNV,adj}) \approx p_{hap1,g,sample1} - p_{hap2,g,sample2} = D \tag{94}$$

and

$$\begin{aligned} var(Z_{hap1,SNV,adj}) \approx & \frac{E(P_{hap1,SNV,sample1,adj})(1 - E(P_{hap1,SNV,sample1,adj}))}{n_{SNV,sample1}} \\ & + \frac{E(P_{hap1,SNV,sample2,adj})(1 - E(P_{hap1,SNV,sample2,adj}))}{n_{SNV,sample2}} \end{aligned} \tag{95}$$

We estimate variance of $Z_{hap1,SNV,adj}$ as

$$\begin{aligned} \widehat{var}(Z_{hap1,SNV,adj}) = & \frac{p'_{hap1,SNV,sample1,adj}(1 - p'_{hap1,SNV,sample1,adj})}{n_{SNV,sample1} + 1} \\ & + \frac{p'_{hap1,SNV,sample2,adj}(1 - p'_{hap1,SNV,sample2,adj})}{n_{SNV,sample2} + 1} \end{aligned} \tag{96}$$

where for $s \in \{sample1, sample2\}$

$$p'_{hap1,SNV,sample1,adj} = \frac{NI(p_{hap1,SNV,s,adj} \times n_{SNV,s}) + 0.5}{n_{SNV,s} + 1} \tag{97}$$

with NI denoting the nearest integer function. This is analogous to the variance estimation approach taken in the case of no pre-existing allelic bias (67).

The pre-existing allelic bias adjustment is incorporated into a 2-sample MBASED analysis in the following ways, which parallel the adjustments made in 1-sample case (section 2.2.4):

1. Haplotype phasing (when true haplotypes are uknown): At each SNV we assign reference allele to haplotype 1 ('major') if
$$p_{ref,SNV,sample1,adj} \geq 0.5 \tag{98}$$
and to haplotype 2 ('minor') otherwise (recall that phasing is done based on sample 1 exclusively).

2. Meta-analysis: Instead of using SNV-level PD-values $z_{hap1,SNV}$ defined in (61), we use the adjusted versions $z_{hap1,SNV,adj}$

3. Variance estimate: We estimate $var(Z_{hap1,SNV,adj})$ as shown in equation (96)

4. Estimating $p_{maj,g}$: Based on the binomial distribution model (83), we re-write the binomial likelihood expression (75) at a single SNV as:
$$L_{SNV}(p_{hap1,g}) = \prod_{s \in \{sample1,sample2\}} \binom{n_{SNV,s}}{x_{hap1,SNV,s}} \times f_{hap1,SNV,s}(p_{hap1,g})^{x_{hap1,SNV,s}} \times f_{hap2,SNV,s}(p_{hap2,g})^{x_{hap2,SNV,s}} \tag{99}$$
where $f_{hap1,SNV,s}(p_{hap1,g})$ is as defined in (6) and is uniquely determined by unknown $p_{hap1,g}$ and $f_{hap1,SNV,s}(0.5)$ (assumed to be known or previously estimated by MBASED). This modification is then used in the procedure described in section 2.3.2.

5. Simulations (when true haplotypes are unknown): For each sample $s \in \{sample1, sample2\}$, we simulate $X_{sim,maj,SNV,s}$ at each locus from a
$$Bin(n = n_{SNV,s}, p = f_{maj,SNV}(\widehat{p}_{maj,g})) \tag{100}$$
distribution, instead of
$$Bin(n = n_{SNV,s}, p = \widehat{p}_{maj,g}) \tag{101}$$
distribution.

Just as with the one-sample algorithm, MBASED requires that values of $f_{hap1,SNV,s}(0.5)$ be supplied for each sample and each SNV. In our study we used sample-specific estimates obtained as described in section 2.2.5.

2.3.5 Adjustment for technical overdispersion

We now extend binomial model (83) to full model (56) used by MBASED, where we allow that
$$\rho_{SNV,s} \neq 0 \tag{102}$$
for at least one sample $s \in \{sample1, sample2\}$.

Following the same reasoning as in section 2.2.6, it is only necessary to consider the effect of overdispersion on the mean and variance of $Z_{hap1,SNV,adj}$. From the properties of beta-binomial distribution in section 2.1.2, for $s \in \{sample1, sample2\}$ we have

$$E(P_{hap1,SNV,s}) = f_{hap1,SNV,s}(p_{hap1,g,s}) \tag{103}$$

and

$$\begin{aligned} var(P_{hap1,SNV,s}) &= f_{hap1,SNV,s}(p_{hap1,g,s}) \times (1 - f_{hap1,SNV,s}(p_{hap1,g,s})) \\ &\times \left(\rho_{SNV,s} + \frac{1-\rho_{SNV,s}}{n_{SNV,s}}\right) \end{aligned} \tag{104}$$

Note that under conditions of no overdispersion ($\rho_{SNV,s} = 0$), expression (104) reduces to

$$var(P_{hap1,SNV,s}) = \frac{f_{hap1,SNV,s}(p_{hap1,g,s}) \times (1 - f_{hap1,SNV,s}(p_{hap1,g,s}))}{n_{SNV,s}} \tag{105}$$

which is identical to previously derived expression (87) for the model with no overdispersion.

Based on the generative beta-binomial model (56) and shifting transformation (90), MBASED relies on the following approximations to take technical overdispersion into account:

$$E(P_{hap1,SNV,s,adj}) \approx p_{hap1,g,s} \tag{106}$$

and

$$\begin{aligned} var(P_{hap1,SNV,s,adj}) &\approx E(P_{hap1,SNV,s,adj}) \times (1 - E(P_{hap1,SNV,s,adj})) \\ &\times \left(\rho_{SNV,s} + \frac{1-\rho_{SNV,s}}{n_{SNV,s}}\right) \end{aligned} \tag{107}$$

Approximation (106) is identical to approximation (92) made in section 2.3.4, and we find that its accuracy is virtually unaffected by the choice of overdispersion parameter $\rho_{SNV,s}$ even for very large values of overdispersion ($\rho_{SNV,s} = 0.1$).

Approximation (107) is analogous to approximation (93) made in section 2.3.4, and we find that it performs very well for moderate values of overdispersion $\rho_{SNV,s} \leq 0.02$ and for moderate-to-high coverage levels ($nSNV, s \geq 20$, data not shown). The performance is especially good for mild levels of pre-existing allelic bias ($0.4 \leq f_{hap1,SNV,s}(p_{hap1,g,s}) \leq 0.6$).

Based on approximations (106) and (107), we conclude that under full model (56), we have

$$E(Z_{hap1,SNV,adj}) \approx p_{hap1,g,sample1} - p_{hap2,g,sample2} = D \tag{108}$$

and

$$
\begin{aligned}
var(Z_{hap1,SNV,adj}) \approx & \Bigg[E(P_{hap1,SNV,sample1,adj}) \times (1 - E(P_{hap1,SNV,sample1,adj})) \\
& \times \left(\rho_{SNV,sample1} + \frac{1-\rho_{SNV,sample1}}{n_{SNV,sample1}}\right)\Bigg] \\
& + \Bigg[E(P_{hap1,SNV,sample2,adj}) \times (1 - E(P_{hap1,SNV,sample2,adj})) \\
& \times \left(\rho_{SNV,sample2} + \frac{1-\rho_{SNV,sample2}}{n_{SNV,sample2}}\right)\Bigg]
\end{aligned} \tag{109}
$$

Analogously to the approach we took in section 2.3.4, we estimate variance of $Z_{hap1,SNV,adj}$ as

$$
\begin{aligned}
\widehat{var}(Z_{hap1,SNV,adj}) = & \Bigg[p'_{hap1,SNV,sample1,adj} \times (1 - p'_{hap1,SNV,sample1,adj}) \\
& \times \left(\rho_{SNV,sample1} + \frac{1-\rho_{SNV,sample1}}{n_{SNV,sample1}+1}\right)\Bigg] \\
& + \Bigg[p'_{hap1,SNV,sample2,adj} \times (1 - p'_{hap1,SNV,sample2,adj}) \\
& \times \rho_{SNV,sample2} + \frac{1-\rho_{SNV,sample2}}{n_{SNV,sample2}+1}\Bigg]
\end{aligned} \tag{110}
$$

where for $s \in \{sample1, sample2\}$

$$
p'_{hap1,SNV,sample1,adj} = \frac{NI(p_{hap1,SNV,s,adj} \times n_{SNV,s}) + 0.5}{n_{SNV,s}+1} \tag{111}
$$

with NI denoting the nearest integer function. Note that we assume that values of $\rho_{SNV,s}$ for both samples are supplied to MBASED (see below).

The adjustment for technical overdispersion is incorporated into a 2-sample MBASED analysis in the following ways, which parallel the adjustments made in 1-sample case (section 2.2.6):

1. Meta-analysis: We use new variance estimates (110) when calculating weights used to derive $z_{hap1,g}$
2. Estimating $p_{maj,g}$: Based on distribution model (56) and properties of beta-binomial distribution, we re-write the binomial likelihood expression (99) at a single SNV as beta-binomial likelihood:

$$
L_{SNV}(p_{hap1,g}) = \prod_{s \in \{sample1, sample2\}} \binom{n_{SNV,s}}{x_{hap1,SNV,s}} \times \frac{B(x_{hap1,SNV,s}+\alpha_s, x_{hap2,SNV,s}+\beta_s)}{B(\alpha_s,\beta_s)} \tag{112}
$$

where

$$\alpha_s = f_{hap1,SNV,s}(p_{hap1,g}) \times \left(\frac{1}{\rho_{SNV,s}} - 1 \right) \tag{113}$$

and

$$\beta_s = (1 - f_{hap1,SNV,s}(p_{hap1,g})) \times \left(\frac{1}{\rho_{SNV,s}} - 1 \right) \tag{114}$$

Since likelihood (112) is undefined when $\rho_{SNV,s} = 0$, we use binomial likelihood expression (99) in such cases. Note that $f_{hap1,SNV,s}(p_{hap1,g})$ is as defined in (6) and is uniquely determined by unknown $p_{hap1,g}$ and $f_{hap1,SNV,s}(0.5)$ (assumed to be known or previously estimated by MBASED). Furthermore, $\rho_{SNV,s}$ is also assumed to be know, leaving $p_{hap1,g}$ as the only variable in the likelihood expression (112). This modified likelihood is then used in the procedure described in section 2.3.2 to obtain $\widehat{p}_{maj,g}$.

3. Simulations (when true haplotypes are unknown): For each sample $s \in \{sample1, sample2\}$, we simulate $X_{sim,maj,SNV,s}$ under the null hypothesis at each locus from a

$$BetaBin(n = n_{SNV,s}, \mu = f_{hap1,SNV,s}(\widehat{p}_{maj,g}), \rho = \rho_{SNV,s}) \tag{115}$$

distribution, instead of from a

$$Bin(n = n_{SNV,s}, p = f_{maj,SNV}(\widehat{p}_{maj,g})) \tag{116}$$

distribution.

Just as with the one-sample algorithm, MBASED requires that values of $\rho_{SNV,s}$ be supplied for each sample and each SNV. In our study we used sample-specific estimates obtained as described in section 2.2.7.

Supplementary Discussion

3 MBASED Performance in Absence of Phasing Information

A natural question to ask is how well MBASED performs when the true haplotypes are unknown, compared to the situation when they are known. We attempted to answer this question by comparing the outcomes of running MBASED with and without provided haplotypes on a data set where the true haplotypes are known. The issue of whether the performance of the 'phased' version of MBASED is sufficient to be used as a 'golden standard' in the comparison we perform here will be addressed in the next section.

We obtained previously published LCL RNA-Seq data and a list of phased genomic variants for (female, non-cancer) individual NA12878, genotyped together with both parents as part of 1000 Genomes Project [7]. The RNA-Seq data was downloaded from GEO using accession number GSE30401. The list of variants, together with the phasing information was downloaded from `http://archive.gersteinlab.org/proj/AlleleSeq/`. Only the phased heterozygous variants were used for the analysis. We pre-processed the data analogously to other samples in our panel,

with exception of filtering based on observed variant allele read frequency at DNA level. This last filter was not preformed as it was not clear which WGS data set available for this individual should be used. Instead, we relied on the variant filtering pipeline employed by the 1000 Genomes Project, and assumed that any remaining heterozygous variants were genuine. We only aligned RNA-Seq data sets corresponding to paired end sequencing results, and pooled all of these runs together, as they appeared to be technical replicates sequenced on the same Illumina flow cell.

We ran MBASED on this data set both with ('phased') and without ('non-phased') specifying the true haplotypes. Overall, we tested 2,560 genes for ASE, including 1,104 (40%) with > 1 heterozygous loci. Using our usual cutoffs (MAF ≥ 0.7, adjusted p-value ≤ 0.05), MBASED found 110 genes to show ASE in the 'phased' setting and 115 genes in the 'non-phased' setting, of which 108 were in common, indicating high degree of consistency. Overall, we observed ASE in $\sim 4.5\%$ of the tested genes, with 45% of ASE genes found on chromosome X, due to chromosome X inactivation in this female individual.

Conceptually, knowing the true haplotypes provides two advantages in ASE detection. First, it increases power to detect ASE. Consider a situation shown below:

Table 1: Haplotype-specific read counts for L3MBTL2 in NA12878 individual

Read Counts	SNV_1	SNV_2
Haplotype 1	26	22
Haplotype 2	15	4

If we do not know the true haplotypes, then we have to assume that the pairs of counts above can be assigned to haplotypes as $\{26, 22\}/\{15, 4\}$ or $\{26, 4\}/\{15, 22\}$. The former case would lead to higher and more significant estimate of gene-level ASE than the latter. Since neither possibility can be excluded, our confidence in ASE of this gene will be lower when we do not know true haplotypes than it would be if we did. This is reflected in MBASED algorithm, where the $\{26, 22\}/\{15, 4\}$ and $\{26, 4\}/\{15, 22\}$ pairs of haplotype counts produce the same MAF estimates, unless we supply true haplotype phasing information. Such situations may lead to ASE being detected by the 'phased' but not the 'non-phased' approach.

Another advantage provided by knowing the true haplotypes is the increased power to detect isoform-specific effects. Consider a situation shown below:

Table 2: Hypothetical haplotype-specific read counts

Read Counts	SNV_1	SNV_2
Haplotype 1	56	4
Haplotype 2	5	41

If we do not know the true haplotypes, the MBASED MAF estimate for this gene will be based on $\{56, 41\}/\{5, 4\}$ pair of haplotype counts, due to pseudo-phasing, likely resulting in a call of highly significant ASE. Importantly, the pseudo-phased allelic counts show high concordance between the two loci and the estimate of inter-loci variability will be low. Thus we will miss entirely the fact that

there is considerable disagreement between the two SNVs within this gene with respect to which haplotype is overexpressed and by how much. On the other hand, if we know the true haplotypes, the MBASED estimate of MAF will be close to 0.5 and the estimate of inter-loci variability will be large, allowing us to detect the disagreement between the individual SNVs. Such behavior may arise, for example, if the two SNVs fall into two exons, used exclusively by two distinct transcript isoforms of the gene, with each transcript isoform subject to allele-specific expression. Such situations may lead to ASE being detected by the 'non-phased' but not the 'phased' approach (although note that the 'phased' approach will produce highly significant measure of inter-loci variability, which can be used to flag such situations for further investigation).

In our acutal data, the two instances where ASE is detected by the 'phased' approach only illustrate the increased power of ASE detection when true underlying haplotypes are known (Table 1). The two gene are UBE3B and L3MBTL2 (the data for L3MBTL2 is shown in Table 1), and the adjusted p-values given by the 'phased' approach are 0.048 and 0.036, respectively, compared to p-values of 0.081 and 0.062, produced by the 'non-phased' approach (the MAF estimates are the same). Note that while the 'non-phased' approach did not declare these genes as showing ASE, the actual adjusted p-values are still close to our chosen cutoff of 0.05. We therefore conclude that at the coverage levels observed in this RNA-Seq data set (inter-quartile range of 17-59 reads/SNV), the additional power provided by knowing the true haplotypes results in very minor improvement in ASE calling.

Out of 7 cases of ASE detected by the 'non-phased' approach only, 3 are due to small differences in adjusted p-values at the margins of our cutoff (e.g, 0.049 vs. 0.0051, Figure S7). The remaining 4 cases represent situations where allelic counts point towards over-expression of different haplotypes at individual loci within a gene (example in Table 2). Of these 4 instances, we find that 3 are likely the artifacts of alignment procedure:

1. RAB5A: There are 2 heterozygous SNVs, with haplotype-specific read counts of $\{48, 0\}/\{19, 11\}$. However, the second (homozygous) SNV is inside the 3' UTR and is within 150bp of an exon of another gene (Figure S19).

2. PPIA: There are 2 heterozygous SNVs, with haplotype-specific read counts of $\{7, 31\}/\{12, 5\}$. Both of the variants are in 3' UTR and visual examination reveals the presence of ~ 10 total 3' UTR variants, although only 5 are reported by 1000 Genomes Project for this individual (Figure S20). Moreover, the gene shows high expression of the intronic regions, indicating the existence of features not captured by the gene models used to guide alignment.

3. SPN: There are 3 heterozygous SNVs, with haplotype-specific read counts of $\{9, 3, 74\}/\{15, 26, 32\}$. All three variants are within 3' UTR and visual examination reveals the presence of ~ 20 total 3' UTR variants, although only 6 are reported by 1000 Genomes Project for this individual (Figure S21).

The remaining case is that of gene MTFP1. There are 2 heterozygous SNVs, with haplotype-specific read counts of $\{11, 51\}/\{12, 18\}$. Here, there seems to be genuine difference between the first SNV (no ASE) and the second SNV (overexpression of first haplotype). The second SNV is located within 3' UTR of the gene, and there are 2 distinct isoforms of MTFP1, suggesting that true isoform-specific ASE may be taking place (Figure S22). However, it should be noted that the estimates of MAF and statistical significance are very similar for both the 'phased' and 'non-phased' approach for this gene (Figure S7). The 'phased' approach estimates MAF as 0.68 (and does not

pass our cutoff), while the 'non-phased' approach resolves the two haplotypes to consist of read count pairs $\{12, 51\}/\{11, 18\}$, resulting in MAF estimate of 0.70 (passing the cutoff).

In addition to the cases of genes declared ASE by the 'phased' or 'non-phased' approaches exclusively, we have also investigated the instances of genes which fail to be correctly resolved into the true underlying haplotypes by the 'non-phased' approach. Such genes may or may not be declared ASE by both approaches. We find that we successfully reconstruct haplotypes of 40/47 (85%) ASE ('non-phased' approach) genes with > 1 heterozygous loci (Additional File 3, Data Set S1), including 18/18 genes on chromosome X, where the signal is the strongest (due to chromosome X inactivation in this female individual). Of the 7 genes where haplotype reconstruction fails, 6 are likely artifacts of alignment procedure, including RAB5A, PPIA, and SPN, discussed above. Another 3 genes (PPM1K, PM20D2, and APOL2) exhibit high levels of expression in intronic regions, combined with the presence of all heterozygous variants in UTR regions (data not shown), similar to the case of PPIA gene. The remaining one instance is the gene MTFP1, discussed above.

We conclude that for the testing data set, the improvements in performance of MBASED obtained by having access to true gene haplotypes are very minor. We further conclude that MBASED exhibits extremely high recovery rate of true underlying haplotypes of ASE genes.

4 Comparison of MBASED to the Method of Skelly et al.

To assess how well MBASED performs when the true haplotypes are known, we compared its performance to the method of Skelly et al. [23] ('Skelly Method'). Skelly method is the only currently published ASE detection approach that allows for variable ASE within a gene. It adopts a Bayesian approach and estimates the gene-level posterior distribution of major allele frequency, as well as the posterior probability of ASE. We adopted the suggestions from the Skelly method tutorial on setting the parameters for MCMC runs. Since we lacked DNA data to estimate prior parameters for Skelly method, we adopted approach identical to Skelly et al. [23], who, when faced with the same predicament, employed the prior parameters from their yeast data analysis. We thus set $\hat{a} = 3600$ and $\hat{d} = 550$. Several runs were performed, yielding very similar results. Only one set of results is discussed here, for clarity of interpretation (Additional File 3, Data Set S1).

To define ASE genes from the output of Skelly method, we required that the posterior probability of ASE be at least 0.95 and that the median of posterior distribution of haplotype 1 frequency was ≥ 0.7 (haplotype 1 is major) or ≤ 0.3 (haplotype 2 is major). This yielded 103 ASE genes, compared to 110 derived by the MBASED 'phased' approach. Of these, 94 genes were common to both methods.

Table **??** shows 9 genes, found to exhibit ASE by Skelly method only. We observed that in all such cases, the SNV coverage was low, and while MBASED produced high estimates of MAF, the adjusted p-values fell slightly short of the chosen cutoff of 0.05 (note that the p-values and MAF estimates reflect adjustment for global reference bias, possibly aggregated across multiple SNVs within the same locus, explaining, e.g, difference between reported values for ACSL4 (2 SNVs merged into a single locus, alternative allele is major for both), APOLD1 (1 SNV, alternative allele is major), and HSPA13 (1 SNV, reference allele is major)).

We further investigated 16 genes which were declared as showing ASE by MBASED, but not by Skelly Method. We find that 15 of these have posterior probability of ASE equal to 1, but fail to pass our imposed major allele frequency cutoff (Skelly Method MAF 0.58-0.69). This is general feature of Skelly Method, and ineed of the Bayesian framework they adopt. Since the prior

Table 3: Genes declared to show ASE by Skelly Method only

Gene	MBASED MAF	MBASED p-value (adj)	Major/minor allele counts
ACSL4	0.94	0.07	10/1
APOLD1	0.93	0.08	10/1
CEBPG	0.90	0.12	11/1
CXorf38	0.91	0.0502	13/1
FAM107B	0.84	0.0504	18/3
HSPA13	0.89	0.17	10/1
MCTS1	0.91	0.08	12/1
STAC3	0.93	0.051	11/1
ZNF14	0.79	0.17	8/2, 9/2

distribution heavily favors the setting of no ASE, this is reflected in the posterior distribution of major allele frequencies shifted towards 0.5 and away from the observed frequencies.

The remaining instance is that of gene UBE3B, which has Skelly Method posterior probability of ASE equal to 0.001 and Skelly Method MAF of 0.51, compared to MBASED adjusted ASE p-value of 0.048 and estimated MAF of 0.78. The observed difference is due to the preprocessing step we took when incorporating pre-existing allelic bias into probabilities of observing reads corresponding to individual haplotypes under the conditions of no ASE. For example, since the estimated pre-existing probability of observing reference allele is 0.52 for this data set, we assume that under conditions of no ASE, we would observe reference allele 52% of the time. The 3 heterozygous SNVs of gene UBE3B were resolved into a single locus by overlapping reads, leading to two haplotypes: one consisting of all alternative alleles and another consisting of all reference alleles. We therefore aggregated the pre-existing allelic biases across the SNVs to yield the no-ASE probability of observing the all-reference haplotype of 0.56, very different from 0.5 assumed by the Skelly Method. Since, in fact, it is the all-alternative haplotype of the UBE3B gene that shows overexpression, MBASED ends up assigning very high significance to the observed ASE, and the adjustment for pre-existing allelic bias shifts the estimated MAF away from 0.5. We note that this observed difference between MBASED and the Skelly Method is not due to an algorithmic difference but to the additional pre-processing employed by us.

Overall, we conclude that the performance of MBASED is qualitatively similar to that of Skelly Method, with main difference being that the Bayesian framework of Skelly Method produces posterior distribution of haplotype frequencies shifted towards 0.5 relative to the observed frequencies, used by MBASED.

Supplementary References

[1] Degner, J.F., Marioni, J.C., Pai, A.A., Pickrell, J.K., Nkadori, E., Gilad, Y., Pritchard, J.K.: Effect of read-mapping biases on detecting allele-specific expression from rna-sequencing data. Bioinformatics **25**(24), 3207–3212 (2009)

[2] Heap, G.A., Yang, J.H., Downes, K., Healy, B.C., Hunt, K.A., Bockett, N., Franke, L., Dubois, P.C., Mein, C.A., Dobson, R.J., *et al.*: Genome-wide analysis of allelic expression imbalance in human primary cells by high-throughput transcriptome resequencing. Human molecular genetics **19**(1), 122–134 (2010)

[3] Satya, R.V., Zavaljevski, N., Reifman, J.: A new strategy to reduce allelic bias in rna-seq readmapping. Nucleic Acids Research **40**(16), 127–127 (2012)

[4] Wu, T.D., Nacu, S.: Fast and snp-tolerant detection of complex variants and splicing in short reads. Bioinformatics **26**(7), 873–881 (2010)

[5] Pickrell, J.K., Marioni, J.C., Pai, A.A., Degner, J.F., Engelhardt, B.E., Nkadori, E., Veyrieras, J.-B., Stephens, M., Gilad, Y., Pritchard, J.K.: Understanding mechanisms underlying human gene expression variation with rna sequencing. Nature **464**(7289), 768–772 (2010)

[6] Esteve-Codina, A., Kofler, R., Palmieri, N., Bussotti, G., Notredame, C., Pérez-Enciso, M.: Exploring the gonad transcriptome of two extreme male pigs with rna-seq. BMC genomics **12**(1), 552 (2011)

[7] Rozowsky, J., Abyzov, A., Wang, J., Alves, P., Raha, D., Harmanci, A., Leng, J., Bjornson, R., Kong, Y., Kitabayashi, N., et al.: Alleleseq: analysis of allele-specific expression and binding in a network framework. Molecular systems biology **7**(1) (2011)

[8] Chiaromonte, F., Yap, V., Miller, W.: Scoring pairwise genomic sequence alignments. In: Pacific Symposium on Biocomputing, vol. 7, p. 115 (2002)

[9] Kent, W.J., Baertsch, R., Hinrichs, A., Miller, W., Haussler, D.: Evolution's cauldron: duplication, deletion, and rearrangement in the mouse and human genomes. Proceedings of the National Academy of Sciences **100**(20), 11484–11489 (2003)

[10] Schwartz, S., Kent, W.J., Smit, A., Zhang, Z., Baertsch, R., Hardison, R.C., Haussler, D., Miller, W.: Human–mouse alignments with blastz. Genome research **13**(1), 103–107 (2003)

[11] Meyer, L.R., Zweig, A.S., Hinrichs, A.S., Karolchik, D., Kuhn, R.M., Wong, M., Sloan, C.A., Rosenbloom, K.R., Roe, G., Rhead, B., *et al.*: The ucsc genome browser database: extensions and updates 2013. Nucleic acids research **41**(D1), 64–69 (2013)

[12] Sherry, S., Ward, M.-H., Kholodov, M., Baker, J., Phan, L., Smigielski, E., Sirotkin, K.: dbsnp: the ncbi database of genetic variation. Nucleic acids research **29**(1), 308–311 (2001)

[13] Iafrate, A.J., Feuk, L., Rivera, M.N., Listewnik, M.L., Donahoe, P.K., Qi, Y., Scherer, S.W., Lee, C.: Detection of large-scale variation in the human genome. Nature genetics **36**(9), 949–951 (2004)

[14] Zhang, J., Feuk, L., Duggan, G., Khaja, R., Scherer, S.: Development of bioinformatics resources for display and analysis of copy number and other structural variants in the human genome. Cytogenetic and genome research **115**(3-4), 205–214 (2006)

[15] Gimelbrant, A., Hutchinson, J.N., Thompson, B.R., Chess, A.: Widespread monoallelic expression on human autosomes. Science **318**(5853), 1136–1140 (2007)

[16] Zhang, K., Li, J.B., Gao, Y., Egli, D., Xie, B., Deng, J., Li, Z., Lee, J.-H., Aach, J., Leproust, E.M., *et al.*: Digital rna allelotyping reveals tissue-specific and allele-specific gene expression in human. Nature methods **6**(8), 613–618 (2009)

[17] Main, B.J., Bickel, R.D., McIntyre, L.M., Graze, R.M., Calabrese, P.P., Nuzhdin, S.V.: Allele-specific expression assays using solexa. BMC genomics **10**(1), 422 (2009)

[18] Ge, B., Pokholok, D.K., Kwan, T., Grundberg, E., Morcos, L., Verlaan, D.J., Le, J., Koka, V., Lam, K.C., Gagné, V., *et al.*: Global patterns of cis variation in human cells revealed by high-density allelic expression analysis. Nature genetics **41**(11), 1216–1222 (2009)

[19] Montgomery, S.B., Sammeth, M., Gutierrez-Arcelus, M., Lach, R.P., Ingle, C., Nisbett, J., Guigo, R., Dermitzakis, E.T.: Transcriptome genetics using second generation sequencing in a caucasian population. Nature **464**(7289), 773–777 (2010)

[20] DerSimonian, R., Laird, N.: Meta-analysis in clinical trials. Controlled clinical trials **7**(3), 177–188 (1986)

[21] Turro, E., Su, S.-Y., Gonçalves, Â., Coin, L., Richardson, S., Lewin, A., *et al.*: Haplotype and isoform specific expression estimation using multi-mapping rna-seq reads. Genome Biol **12**(2), 13 (2011)

[22] Emerson, J., Hsieh, L.-C., Sung, H.-M., Wang, T.-Y., Huang, C.-J., Lu, H.H.-S., Lu, M.-Y.J., Wu, S.-H., Li, W.-H.: Natural selection on cis and trans regulation in yeasts. Genome research **20**(6), 826–836 (2010)

[23] Skelly, D.A., Johansson, M., Madeoy, J., Wakefield, J., Akey, J.M.: A powerful and flexible statistical framework for testing hypotheses of allele-specific gene expression from rna-seq data. Genome research **21**(10), 1728–1737 (2011)

[24] Schwarzer, G.: Meta: Meta-Analysis with R. (2012). R package version 2.1-4. `http://CRAN.R-project.org/package=meta`

[25] Freeman, M.F., Tukey, J.W.: Transformations related to the angular and the square root. The Annals of Mathematical Statistics **21**(4), 607–611 (1950)

[26] Miller, J.J.: The inverse of the freeman–tukey double arcsine transformation. The American Statistician **32**(4), 138–138 (1978)

[27] Kulinskaya, E., Morgenthaler, S., Staudte, R.G.: Variance stabilizing the difference of two binomial proportions. The American Statistician **64**(4), 350–356 (2010)

Supplementary Figures

Figure S1. True positive rate (TPR) and false discovery rate (FDR) for 1-sample simulation results in absence of overdispersion ($\rho = 0$). Note that MBASED performance improves (higher TPR at the same nominally controlled FDR of 5%), especially at low signal (major allele frequency (MAF) = 0.7) as overdispersion decreases (cf. Figure 2, where we used value $\rho = 0.004$, estimated from real data (Supplementary Methods)). SE: estimated standard error.

Figure S2. True positive rate (TPR) and false discovery rate (FDR) for 1-sample simulation results at high levels of overdispersion ($\rho = 0.02$). Note that MBASED performances worsens (lower TPR at the same nominally controlled FDR of 5%), especially at low signal (major allele frequency (MAF) = 0.7) as overdispersion increases (cf. Figure 2, where where we used value $\rho = 0.004$, estimated from real data (Supplementary Methods)).

Figure S3. True positive rate (TPR) and false discovery rate (FDR) for 2-sample simulation results in absence of overdispersion ($\rho = 0$). Note that MBASED performance improves (higher TPR at the same nominally controlled FDR of 5%) as overdispersion decreases (cf. Figure 3, where where we used value $\rho = 0.004$, estimated from real data (Supplementary Methods)). MAF: major allele frequency. SE: estimated standard error.

Figure S4. True positive rate (TPR) and false discovery rate (FDR) for 2-sample simulation results at high levels of overdispersion ($\rho = 0.02$). Note that MBASED performance worsens (lower TPR at the same nominally controlled FDR of 5%), especially at low signal (major allele frequency (MAF) = 0.7) as overdispersion increases (cf. Figure 3, where where we used value $\rho = 0.004$, estimated from real data (Supplementary Methods)). SE: estimated standard error.
Genes called ASE by 'phased' approach only are shown in red. Genes called ASE by 'non-phased' approach only are shown in blue.
Figure S5. True positive rate (TPR) and false discovery rate (FDR) for 1-sample simulation results in presence of pre-existing allelic bias ($P_{ref} = 0.6$). Note that MBASED performs virtually the same as in the setting of no bias (cf. Figure 2, where $P_{ref} = 0.5$). The value of allelic bias chosen here is much more extreme than what we observe in our data ($P_{ref} \approx 0.52$, cf. Supplementary Figure 12). MAF: major allele frequency. SE: estimated standard error.

Figure S6. True positive rate (TPR) and false discovery rate (FDR) for 2-sample simulation results in presence of pre-existing allelic bias ($P_{ref,normal} = 0.6, P_{ref,tumor} = 0.5$). Note that MBASED performs virtually the same as in the setting of no bias (cf. Figure 3, where $P_{ref,normal} = P_{ref,tumor} = 0.5$). We also observe the same behavior if we let ($P_{ref,normal} = 0.5, P_{ref,tumor} = 0.6$) or ($P_{ref,normal} = P_{ref,tumor} = 0.6$) (not shown). The value of allelic bias chose here is much more extreme than what we observe in our data ($P_{ref} \approx 0.52$, cf. Supplementary Figure 12). MAF: major allele frequency. SE: estimated standard error.

Figure S7. Comparison of MBASED performance on NA12878 RNA-Seq data with ('phased') and without ('non-phased') supplying true gene haplotypes. Genes called ASE by 'phased' approach only are shown in red. Genes called ASE by 'non-phased' approach only are shown in blue. **A:** Adjusted p-value. In 3 instances (FAM107B, STAC3, CXorf38), the difference in ASE status is due to adjusted p-values falling close to used cutoff (0.05), but on different sides of it. **B:** Major allele

frequency (MAF).

Figure S8. Chromosome 12-specific genes with recurrent ASE in cancer tissue samples (cf. Figure 6A). X-axis correspond to the entire chromosome. Y-axis represents copy number (CN) state as l2r (log2 tumor/normal ratio). Horizontal segments show CN state along chromosome (red: detected allelic imbalance (AI); black: no detected AI). Green lines on top and bottom show globally estimated cutoffs for CN gain and CN loss, respectively (in some instances the global cutoffs do not fit chromosome-specific data well). Genes with recurrently detected ASE are plotted next to the segments in which they are located (connected with vertical dotted lines): black: gene not tested for ASE in this sample; red: gene found to show ASE in this sample; blue: gene found to not show ASE in this sample. The rug along the x-axis shows the location of all tested genes found to show ASE (red) and not to show ASE (blue). Recurrent CN alterations involving KRAS leads to recurrent ASE of nearby genes.

Figure S9. Chromosome 17-specific genes with recurrent ASE in cancer tissue samples (cf. Figure 6A and Supplementary Figure 8). In this particular case, the recurrently deleted genomic fragment contains TP53, a known tumor suppressor. This recurrent deletion leads to recurrent ASE of nearby genes (likely due to heterozygous calls resulting from normal admixture).

Figure S10. Loss of monoallelic ASE in tumors. **A:** ASE status of genes, as determined by MBASED, that show monoallelic expression in at least 1 normal sample. MAF: major allele frequency. Rows are genes, columns are samples. Any gene that did not contain informative SNVs for a tumor-normal comparison is labeled in gray. Genes that show normal-specific ASE (loss-of-ASE in tumor), as identified by two-sample MBASED algorithm, are labeled with a black dot. Notice that there are no recurrent loss-of-ASE events. Known imprinted genes are identified by red gene names. Note that no loss-of-imprinting events are detected. **B:** Example of loss of ASE in tumor, leading to biallelic expression. **C:** Example of loss of ASE in tumor, leading to reversal of ASE pattern.

Figure S11. Percent of tested genes on chromosome X found to show ASE in female samples. In all cases (except H2009, which lost a copy of chromosome X), this percent is higher than the overall ASE rate, likely driven by chromosome X inactivation events. The percent is also much higher in cancer than in normal samples.

Figure S12. Observed reference bias. For each sample, we take all heterozygous exonic SNVs, tally the number of RNA-Seq reads across all SNVs that support reference allele and divide this number by the total number of reads covering such SNVs (top 5% of SNVs by read count are excluded from the calculation). The resulting fraction is multiplied by 100 and plotted. A small but consistent preference for reference allele is observed.

Figure S13. Strong ASE signal due to artifacts. **A:** Top 20 most commonly ASE-exhibiting (1-sample analysis) genes prior to filtering. Note that some genes (e.g. MAP2K3) show ASE in almost every single sample. **B:** Percent of SNVs in top 20 most commonly ASE-exhibiting genes that are filtered out by VPKE, DGV, and selfChain filters.

Figure S14. Spurious ASE in MAP2K3 gene. Snapshot from Integrated Genome Viewer. The

top 2 tracks show RNA-Seq and DNA-Seq coverage for the H1299 cell line. Tracks 3 and 4 show RNA-Seq and DNA-Seq coverage for the H1993 cell line. Tracks 5 and 6 show DNA-Seq alignments for the H1993 cell line from different platforms. There appear to be 2 distinct haplotypes: reference and alternative, with the latter haplotype containing all of the variants (except for the single variant appearing in RNA-Seq data). It is likely that a non-expressed homologous region absent from the reference genome is responsible for these observations. The tracks for other studied samples are similar.

Figure S15. Spurious ASE in SEC22B gene. Snapshot from Integrated Genome Viewer. The top 2 tracks show RNA-Seq and DNA-Seq coverage for the H1299 cell line. Tracks 3 and 4 show RNA-Seq and DNA-Seq coverage for the H1993 cell line. Track 5 shows DNA-Seq alignments for the H1993 cell line. There appear to be 3 distinct haplotypes, and similar observations hold for other studied samples.

Figure S16. Effects of shifting transformation to adjust for pre-existing allelic bias. FT-value corresponding to true reference allele frequency (x-axis) is plotted against average FT-value obtained form simulations before (red) and after (blue) the shifting adjustment. Counts were simulated with total coverage of 20 reads. Each panel corresponds to a specific value of pre-existing allelic bias (p_ref, probability of observing reference allele under conditions of no ASE). The adjustment performs well for mild values of pre-existing allelic bias. The performance is almost identical for both lower (10 reads) and higher (50 reads) values of total coverage (not shown).

Figure S17. Effects of shifting transformation to adjust for pre-existing allelic bias. True reference allele frequency (x-axis) is plotted against average backtransformed FT-value obtained from simulations after the shifting adjustment. Counts were simulated with total coverage of 20 reads. Each panel corresponds to a specific value of pre-existing allelic bias (p_ref, probability of observing reference allele under conditions of no ASE). The adjustment performs well for mild values of pre-existing allelic bias. The performance is almost identical for both lower (10 reads) and higher (50 reads) values of total coverage (not shown).

Figure S18. The observed (in red) and estimated (blue) variance of the backtransformed proportion after the shifting adjustment for pre-existing allelic bias. True reference allele frequency is plotted on x-axis. Counts were simulated with total coverage of 20 reads. Each panel corresponds to a specific value of pre-existing allelic bias (p_ref, probability of observing reference allele under conditions of no ASE). The adjustment performs well for mild values of pre-existing allelic bias. The performance is slightly worse for lower (10 reads) and slightly better for higher (50 reads) values of total coverage (not shown).

Figure S19. Possibly spurious ASE in RAB5A gene. Snapshot from Integrated Genome Viewer. The tracks show RNA-Seq coverage for NA12878 individual. The homozygous (green) variant in 3' UTR of RAB5A is within 125bp of PP2D1 gene (bottom isoform), suggesting possible alignment artifact.

Figure S20. Possibly spurious ASE in PPIA gene. Snapshot from Integrated Genome Viewer. The tracks show RNA-Seq coverage for NA12878 individual. There appear to be ~ 10 variants in 3' UTR of PPIA, but only 5 are reported for this individual by 1000 genomes project. This, along

with high inronic expression indicates possible alignment artifact.

Figure S21. Possibly spurious ASE in SPN gene. Snapshot from Integrated Genome Viewer. The tracks show RNA-Seq coverage for NA12878 individual. There appear to be ~ 20 variants in 3' UTR of PPIA, but only 6 are reported for this individual by 1000 genomes project, suggesting possible alignment artifact.

Figure S22. Possible isoform-specific ASE in MTFP1 gene. Snapshot from Integrated Genome Viewer. The tracks show RNA-Seq coverage for NA12878 individual. An upstream SNV (red/blue) does not show ASE, while the downstream SNV (green/brown) does. A possible explanation is that the bottom transcript isoform shows equal allelic expression, while the top transcript isoform preferentially expresses the green allele.

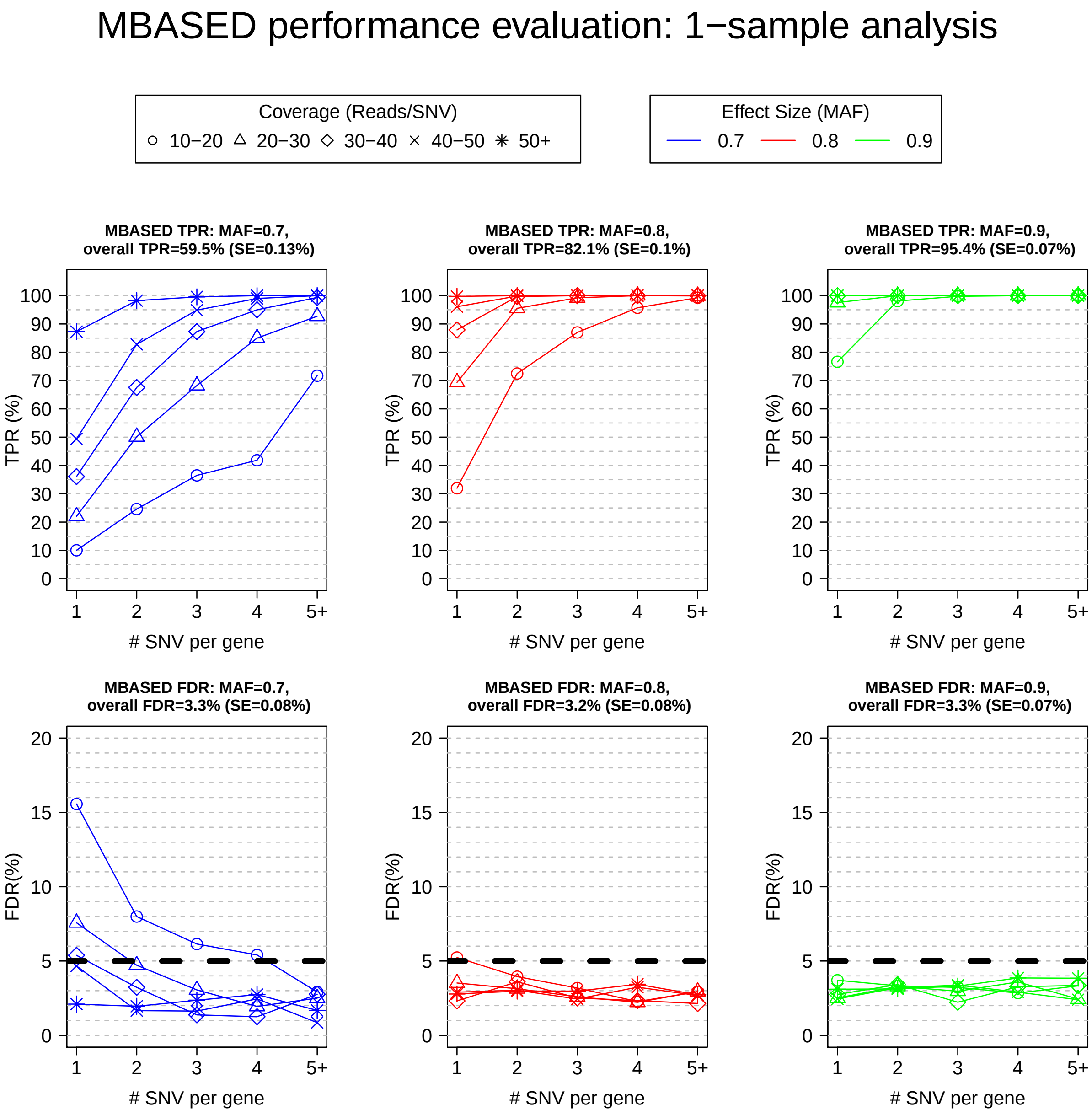
MBASED performance evaluation: 1−sample analysis
Coverage (Reads/SNV)
10−20 20−30 30−40 40−50 50+
Effect Size (MAF)
0.7 0.8 0.9
MBASED TPR: MAF=0.7, overall TPR=59.5% (SE=0.13%)
MBASED TPR: MAF=0.8, overall TPR=82.1% (SE=0.1%)
MBASED TPR: MAF=0.9, overall TPR=95.4% (SE=0.07%)
TPR (%)
SNV per gene
MBASED FDR: MAF=0.7, overall FDR=3.3% (SE=0.08%)
MBASED FDR: MAF=0.8, overall FDR=3.2% (SE=0.08%)
MBASED FDR: MAF=0.9, overall FDR=3.3% (SE=0.07%)
FDR(%)
1 2 3 4 5+

Figure S1

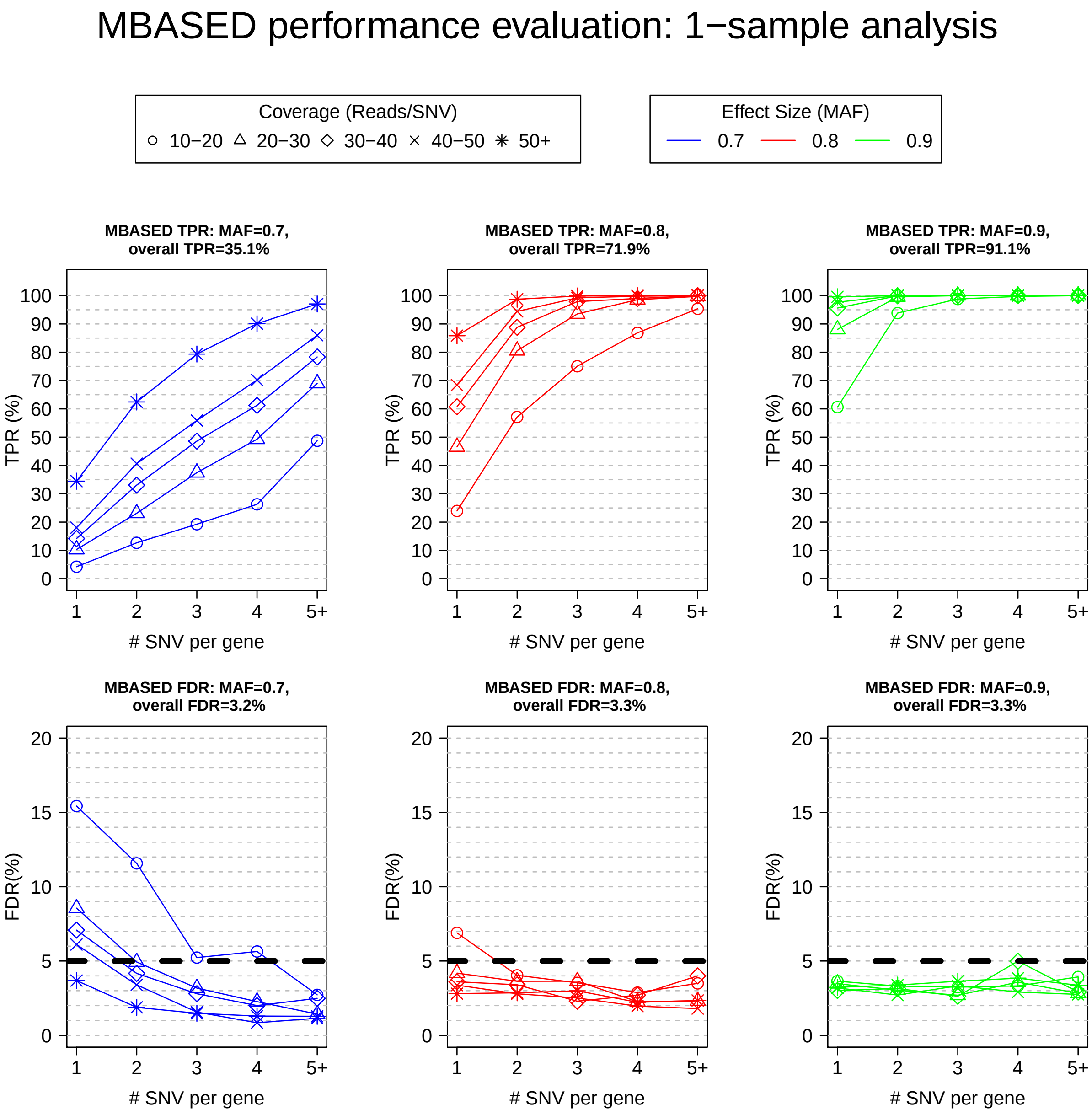
MBASED performance evaluation: 1−sample analysis
Coverage (Reads/SNV)
10−20
20−30
30−40
40−50
50+
Effect Size (MAF)
0.7
0.8
0.9
MBASED TPR: MAF=0.7, overall TPR=35.1%
MBASED TPR: MAF=0.8, overall TPR=71.9%
MBASED TPR: MAF=0.9, overall TPR=91.1%
TPR (%)
SNV per gene
MBASED FDR: MAF=0.7, overall FDR=3.2%
MBASED FDR: MAF=0.8, overall FDR=3.3%
MBASED FDR: MAF=0.9, overall FDR=3.3%
FDR(%)
# SNV per gene

Figure S2

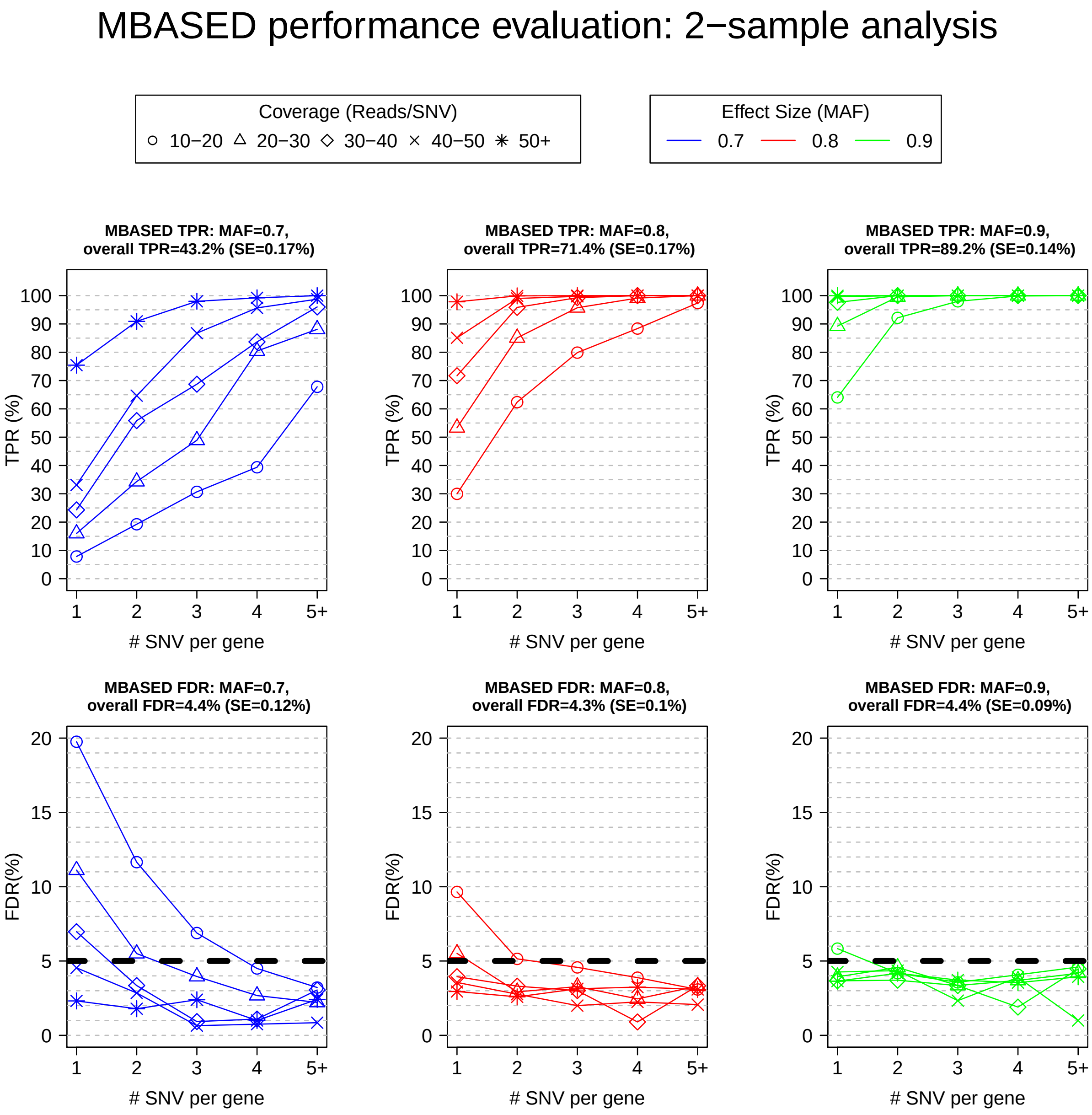
MBASED performance evaluation: 2−sample analysis
Coverage (Reads/SNV)
10−20 20−30 30−40 40−50 50+
Effect Size (MAF)
0.7 0.8 0.9
MBASED TPR: MAF=0.7, overall TPR=43.2% (SE=0.17%)
MBASED TPR: MAF=0.8, overall TPR=71.4% (SE=0.17%)
MBASED TPR: MAF=0.9, overall TPR=89.2% (SE=0.14%)
TPR (%)
SNV per gene
MBASED FDR: MAF=0.7, overall FDR=4.4% (SE=0.12%)
MBASED FDR: MAF=0.8, overall FDR=4.3% (SE=0.1%)
MBASED FDR: MAF=0.9, overall FDR=4.4% (SE=0.09%)
FDR(%)
# SNV per gene

Figure S3

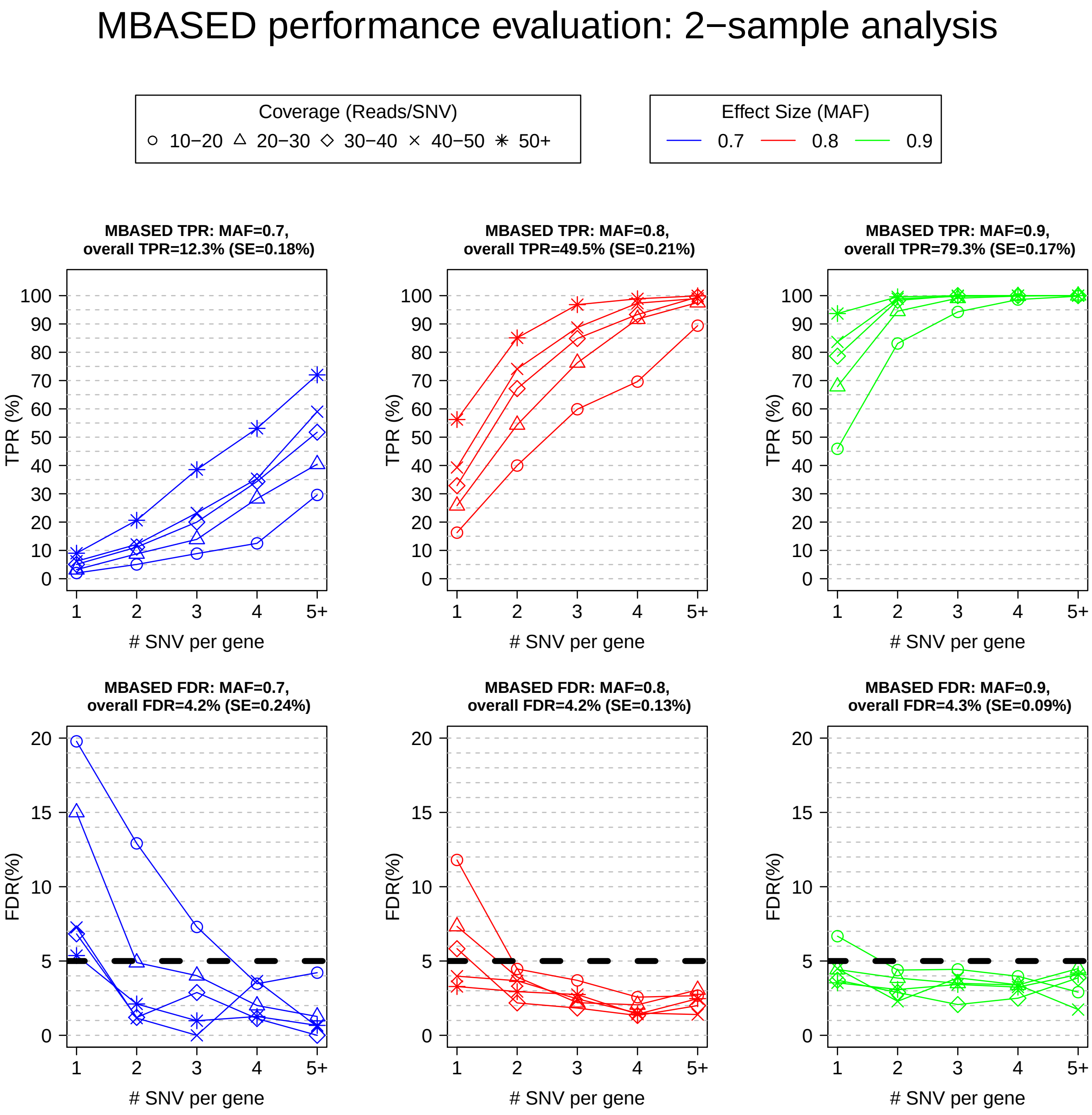
MBASED performance evaluation: 2−sample analysis
Coverage (Reads/SNV)
10−20 20−30 30−40 40−50 50+
Effect Size (MAF)
0.7 0.8 0.9
MBASED TPR: MAF=0.7, overall TPR=12.3% (SE=0.18%)
MBASED TPR: MAF=0.8, overall TPR=49.5% (SE=0.21%)
MBASED TPR: MAF=0.9, overall TPR=79.3% (SE=0.17%)
TPR (%)
MBASED FDR: MAF=0.7, overall FDR=4.2% (SE=0.24%)
MBASED FDR: MAF=0.8, overall FDR=4.2% (SE=0.13%)
MBASED FDR: MAF=0.9, overall FDR=4.3% (SE=0.09%)
FDR(%)
# SNV per gene

Figure S4

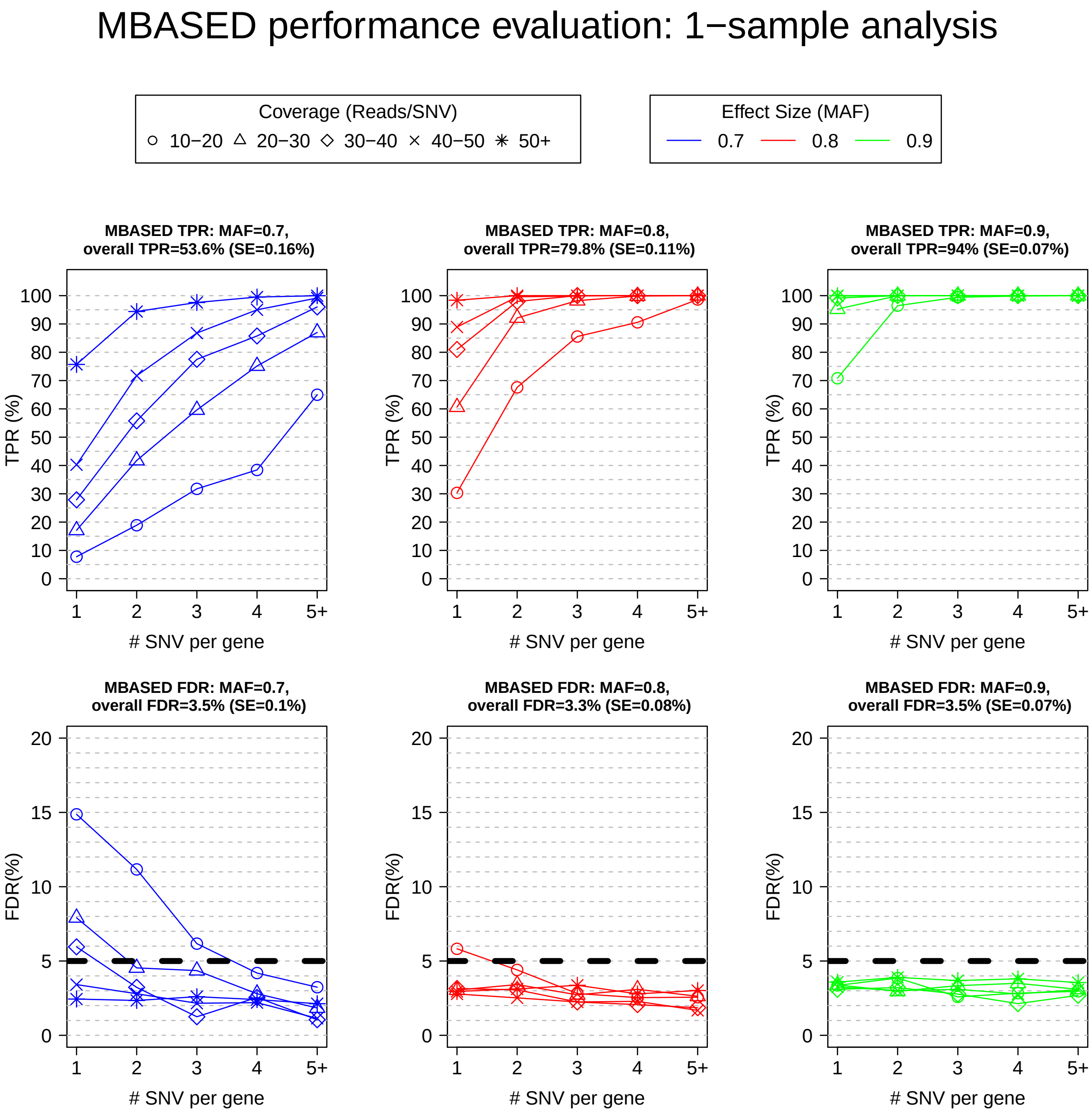
MBASED performance evaluation: 1−sample analysis
Coverage (Reads/SNV)
10−20 20−30 30−40 40−50 50+
Effect Size (MAF)
0.7 0.8 0.9
MBASED TPR: MAF=0.7, overall TPR=53.6% (SE=0.16%)
MBASED TPR: MAF=0.8, overall TPR=79.8% (SE=0.11%)
MBASED TPR: MAF=0.9, overall TPR=94% (SE=0.07%)
MBASED FDR: MAF=0.7, overall FDR=3.5% (SE=0.1%)
MBASED FDR: MAF=0.8, overall FDR=3.3% (SE=0.08%)
MBASED FDR: MAF=0.9, overall FDR=3.5% (SE=0.07%)
TPR (%)
FDR(%)
# SNV per gene
1 2 3 4 5+

Figure S5

MBASED performance evaluation: 2−sample analysis

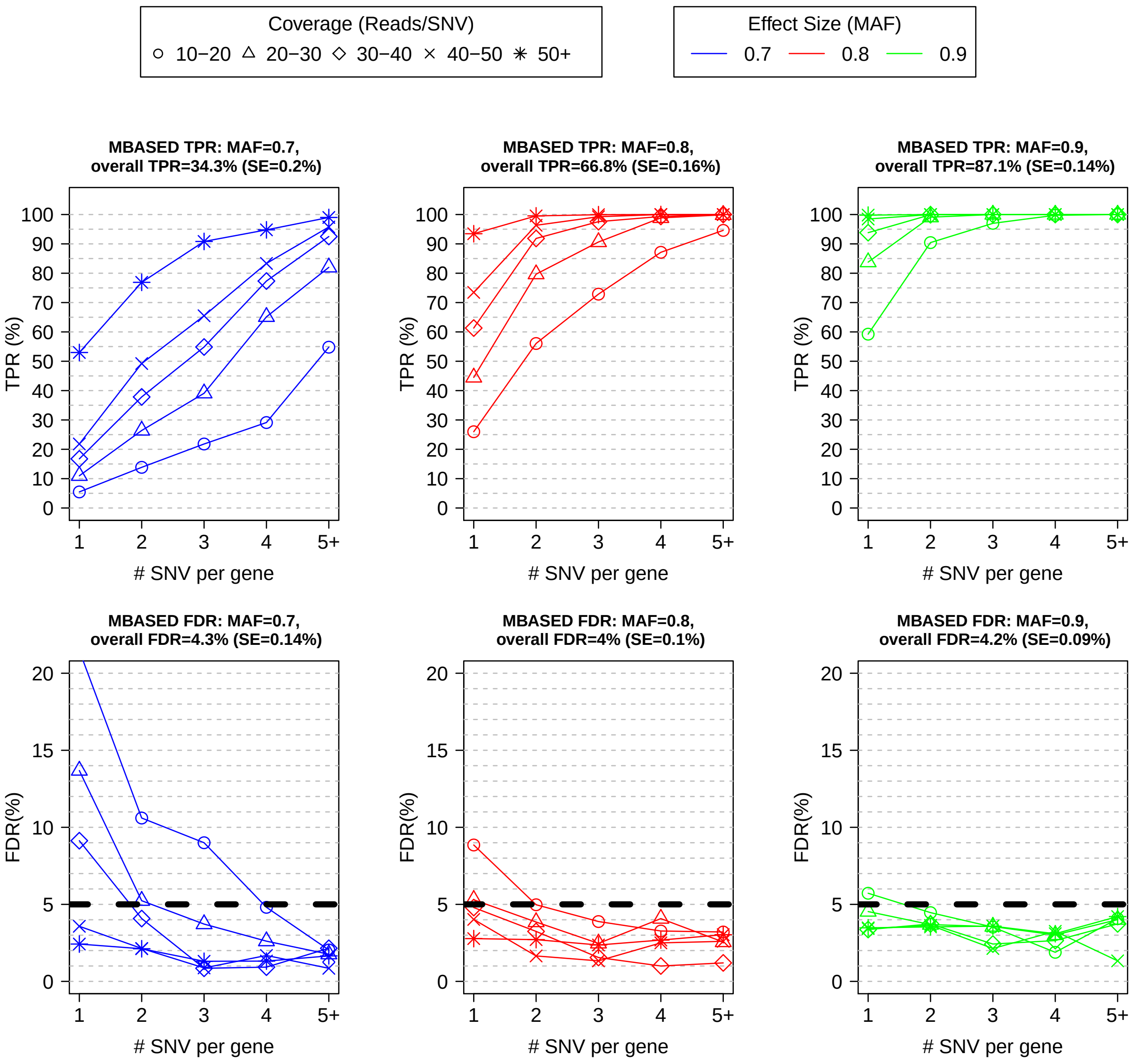

Figure S6

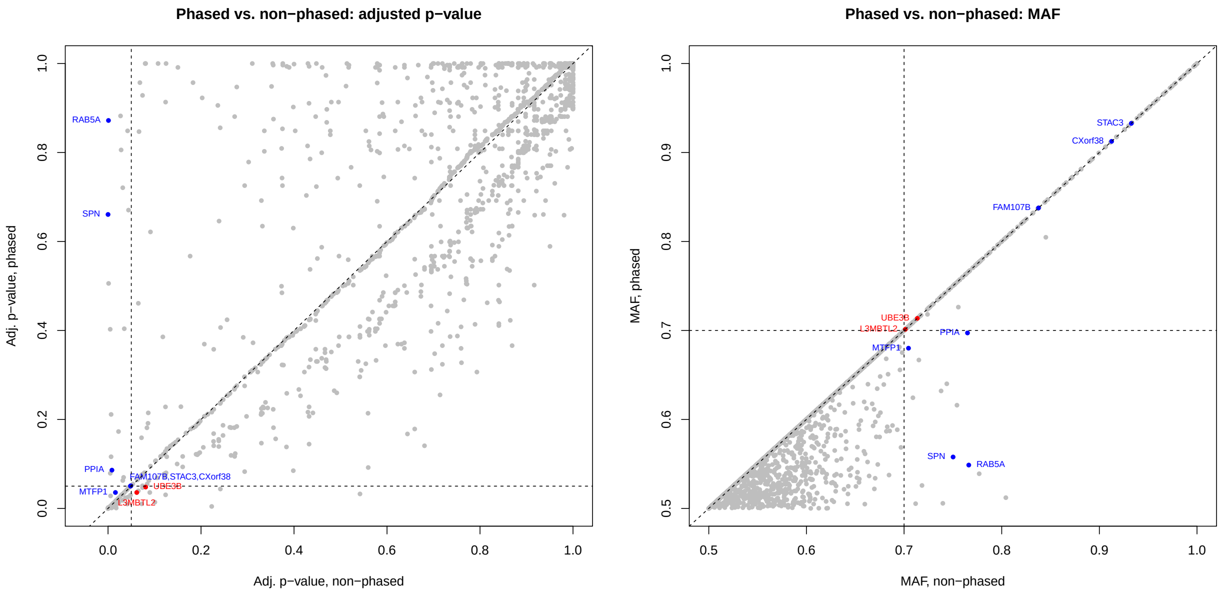

Phased vs. non−phased: adjusted p−value
Adj. p−value, phased
Adj. p−value, non−phased
0.0
0.2
0.4
0.6
0.8
1.0
RAB5A
SPN
PPIA
MTFP1
FAM107B,STAC3,CXorf38
UBE3B
L3MBTL2
Phased vs. non−phased: MAF
MAF, phased
MAF, non−phased
0.5
0.6
0.7
0.8
0.9
1.0
STAC3
CXorf38
FAM107B
UBE3B
L3MBTL2
PPIA
MTFP1
SPN
RAB5A

Figure S7

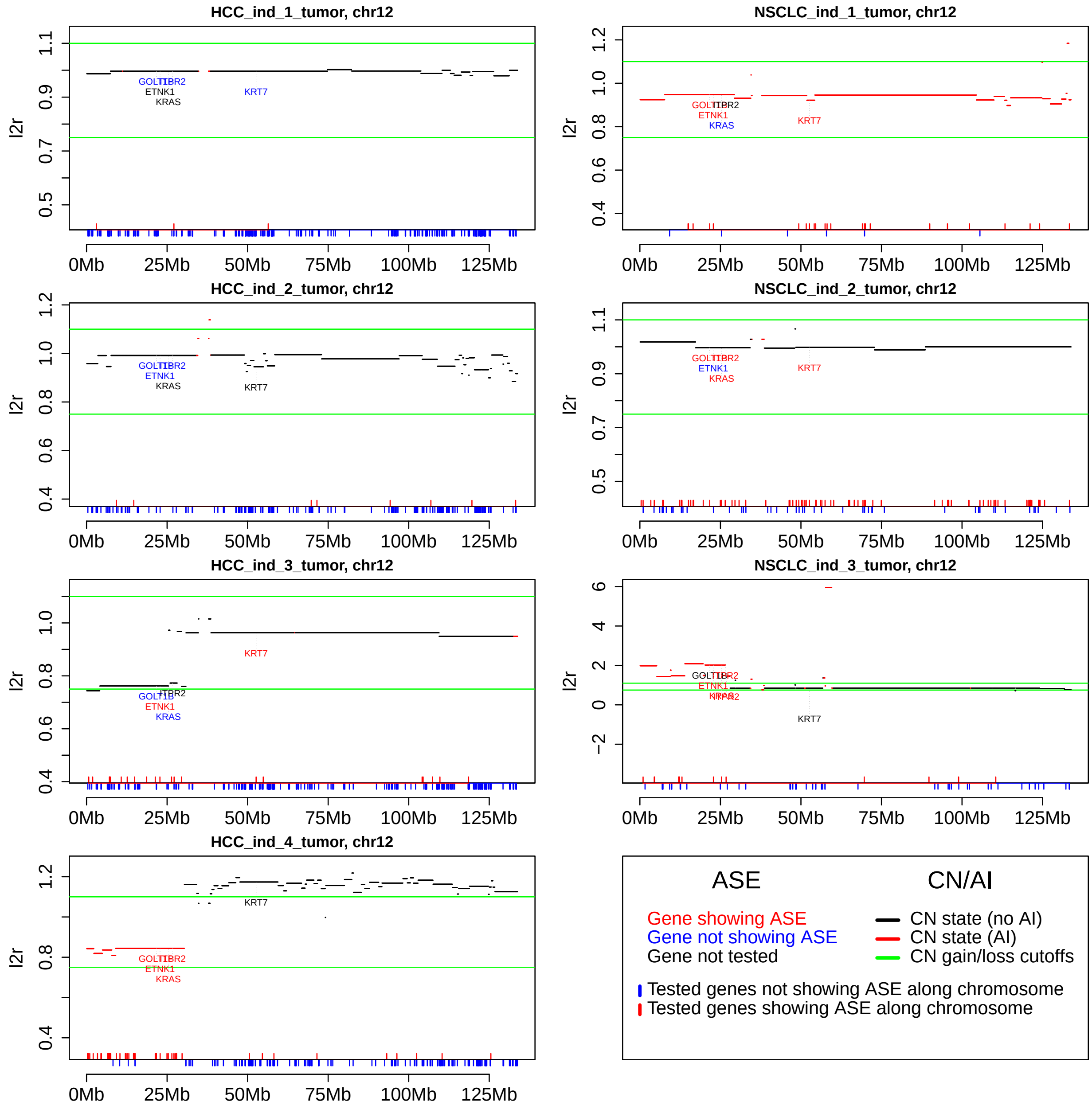
HCC_ind_1_tumor, chr12
NSCLC_ind_1_tumor, chr12
HCC_ind_2_tumor, chr12
NSCLC_ind_2_tumor, chr12
HCC_ind_3_tumor, chr12
NSCLC_ind_3_tumor, chr12
HCC_ind_4_tumor, chr12
l2r
0Mb
25Mb
50Mb
75Mb
100Mb
125Mb
GOLT1B
TBR2
ETNK1
KRAS
KRT7
ASE
CN/AI
Gene showing ASE
Gene not showing ASE
Gene not tested
CN state (no AI)
CN state (AI)
CN gain/loss cutoffs
Tested genes not showing ASE along chromosome
Tested genes showing ASE along chromosome

Figure S8

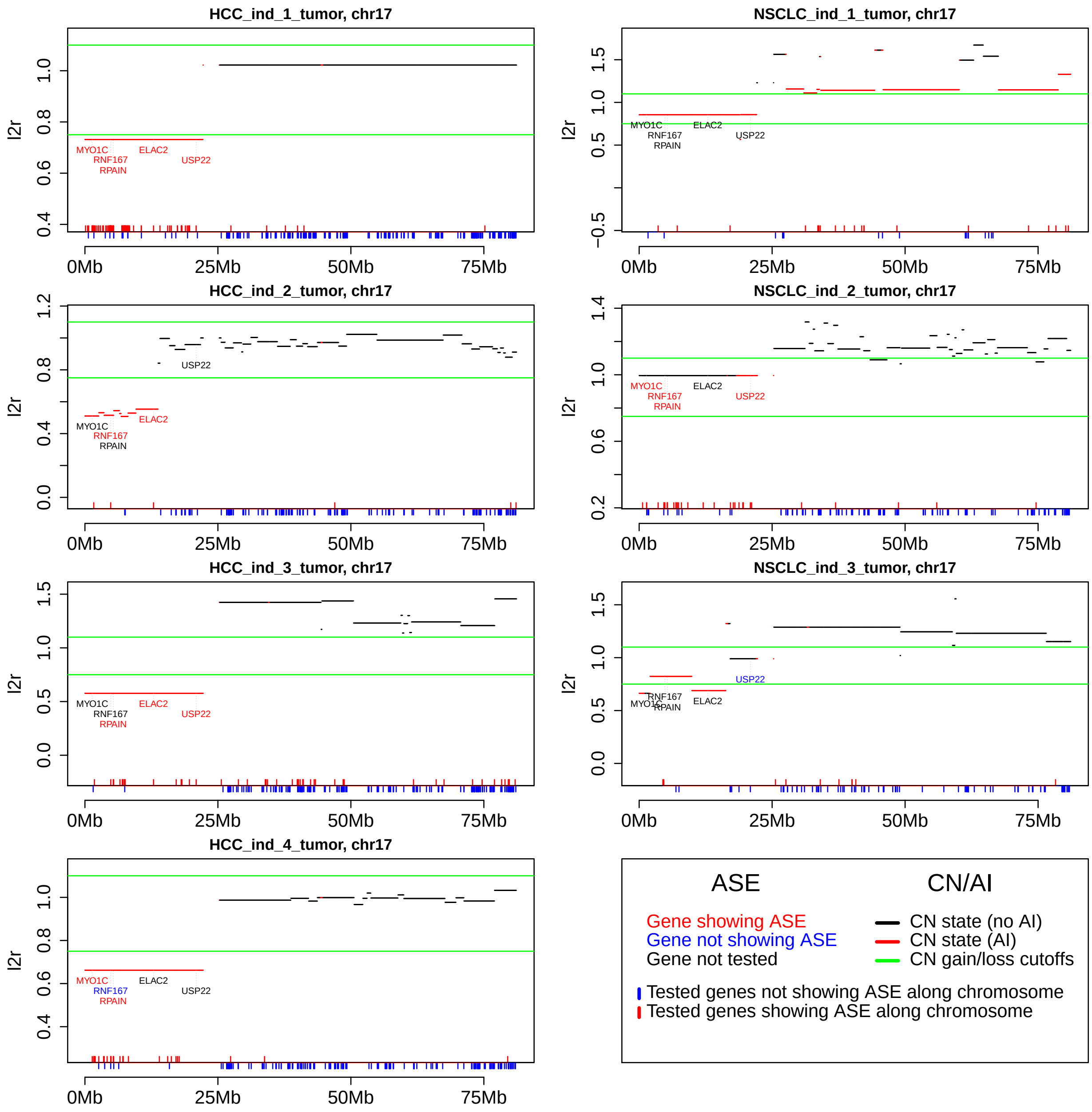
HCC_ind_1_tumor, chr17
NSCLC_ind_1_tumor, chr17
HCC_ind_2_tumor, chr17
NSCLC_ind_2_tumor, chr17
HCC_ind_3_tumor, chr17
NSCLC_ind_3_tumor, chr17
HCC_ind_4_tumor, chr17
l2r
0Mb
25Mb
50Mb
75Mb
MYO1C
RNF167
RPAIN
ELAC2
USP22
ASE
CN/AI
Gene showing ASE
Gene not showing ASE
Gene not tested
CN state (no AI)
CN state (AI)
CN gain/loss cutoffs
Tested genes not showing ASE along chromosome
Tested genes showing ASE along chromosome

Figure S9

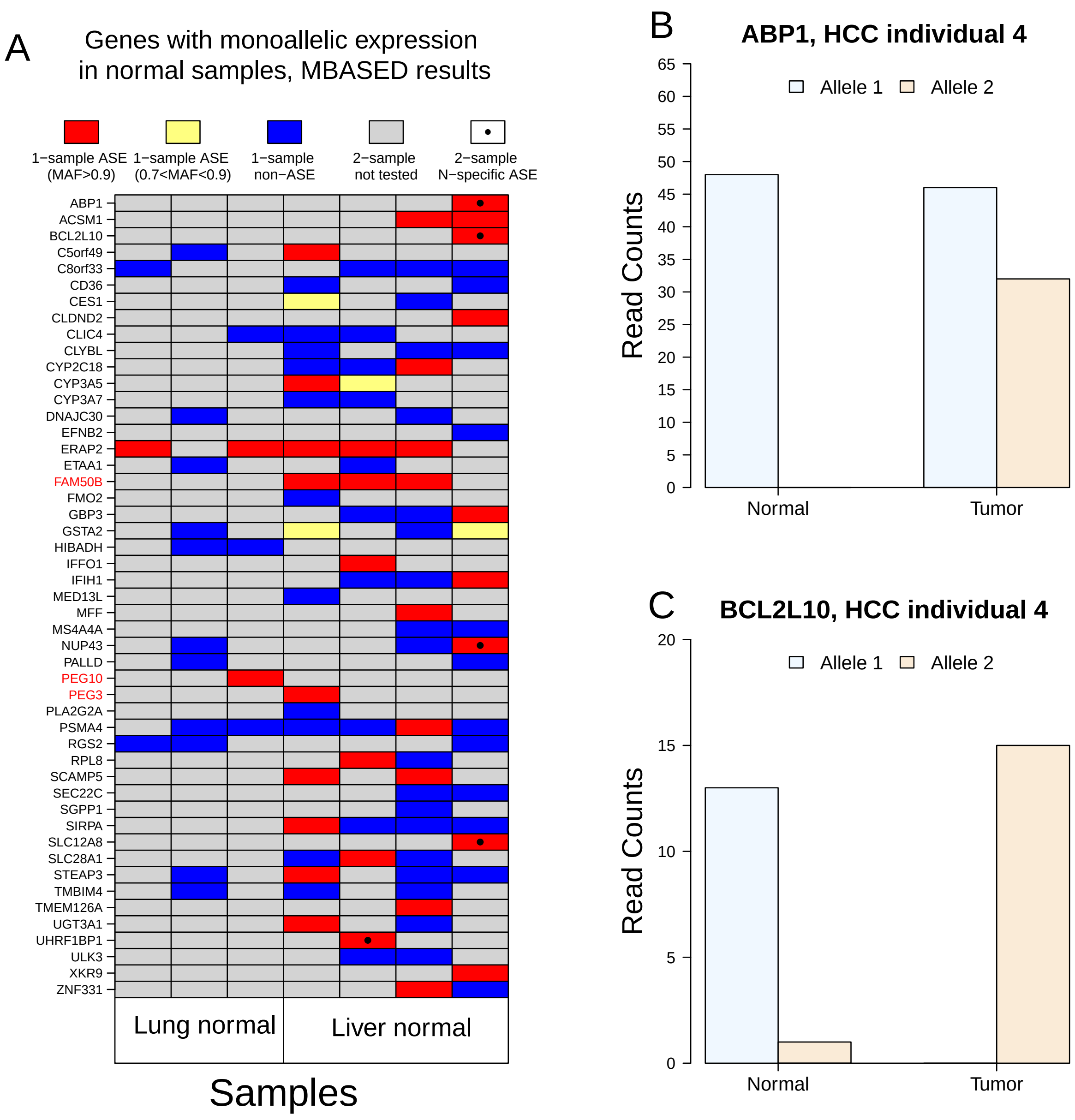
A
Genes with monoallelic expression in normal samples, MBASED results
1−sample ASE (MAF>0.9)
1−sample ASE (0.7<MAF<0.9)
1−sample non−ASE
2−sample not tested
2−sample N−specific ASE
ABP1
ACSM1
BCL2L10
C5orf49
C8orf33
CD36
CES1
CLDND2
CLIC4
CLYBL
CYP2C18
CYP3A5
CYP3A7
DNAJC30
EFNB2
ERAP2
ETAA1
FAM50B
FMO2
GBP3
GSTA2
HIBADH
IFFO1
IFIH1
MED13L
MFF
MS4A4A
NUP43
PALLD
PEG10
PEG3
PLA2G2A
PSMA4
RGS2
RPL8
SCAMP5
SEC22C
SGPP1
SIRPA
SLC12A8
SLC28A1
STEAP3
TMBIM4
TMEM126A
UGT3A1
UHRF1BP1
ULK3
XKR9
ZNF331
Lung normal
Liver normal
Samples
B
ABP1, HCC individual 4
Allele 1
Allele 2
Read Counts
0
5
10
15
20
25
30
35
40
45
50
55
60
65
Normal
Tumor
C
BCL2L10, HCC individual 4
Allele 1
Allele 2
Read Counts
0
5
10
15
20
Normal
Tumor

Figure S10

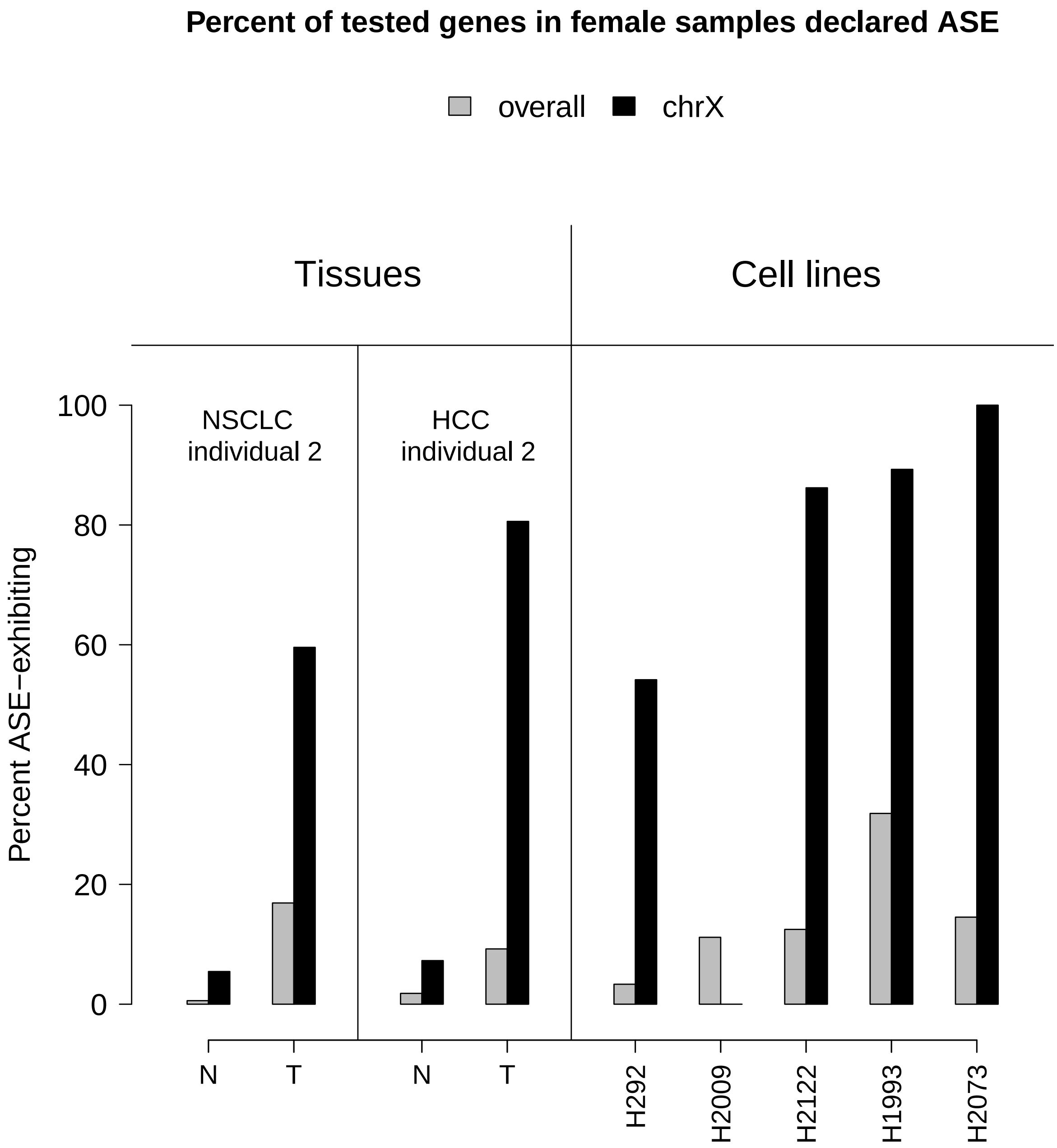
Percent of tested genes in female samples declared ASE
overall
chrX
Tissues
Cell lines
NSCLC
individual 2
HCC
individual 2
Percent ASE−exhibiting
0
20
40
60
80
100
N
T
N
T
H292
H2009
H2122
H1993
H2073

Figure S11

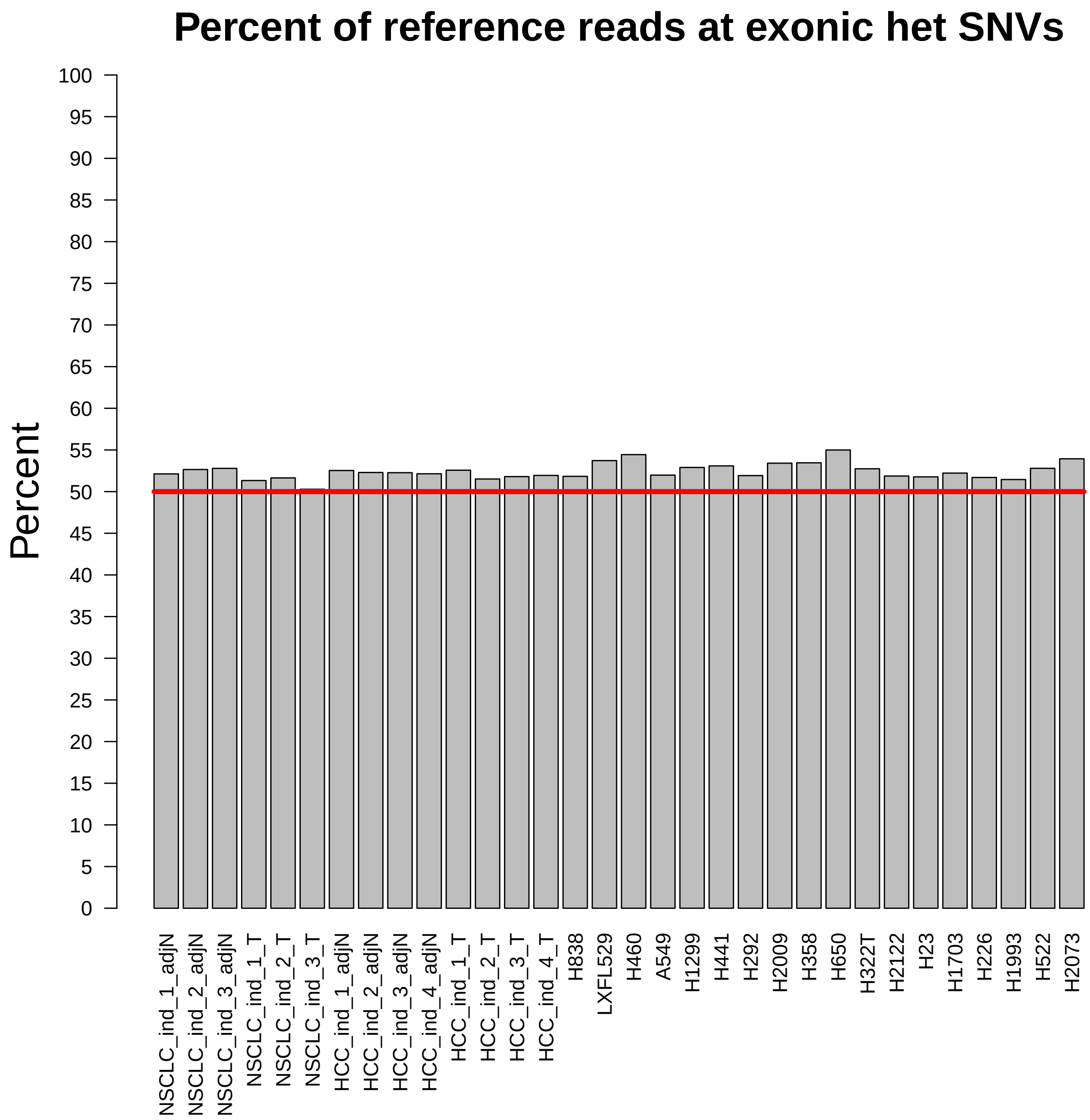

Percent of reference reads at exonic het SNVs
Percent
100
95
90
85
80
75
70
65
60
55
50
45
40
35
30
25
20
15
10
5
0
NSCLC_ind_1_adjN
NSCLC_ind_2_adjN
NSCLC_ind_3_adjN
NSCLC_ind_1_T
NSCLC_ind_2_T
NSCLC_ind_3_T
HCC_ind_1_adjN
HCC_ind_2_adjN
HCC_ind_3_adjN
HCC_ind_4_adjN
HCC_ind_1_T
HCC_ind_2_T
HCC_ind_3_T
HCC_ind_4_T
H838
LXFL529
H460
A549
H1299
H441
H292
H2009
H358
H650
H322T
H2122
H23
H1703
H226
H1993
H522
H2073

Figure S12

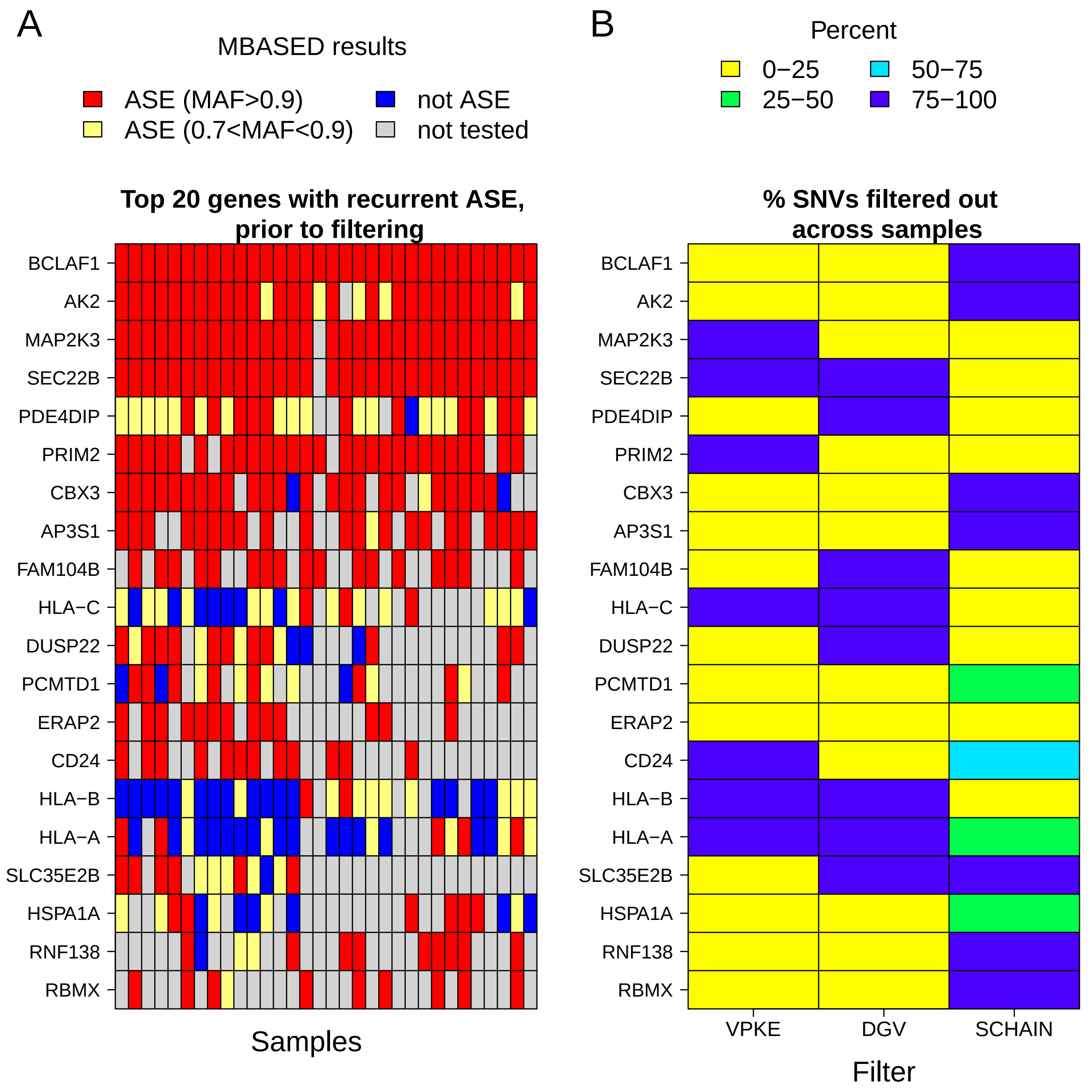
A
MBASED results
ASE (MAF>0.9)
ASE (0.7<MAF<0.9)
not ASE
not tested
Top 20 genes with recurrent ASE, prior to filtering
BCLAF1
AK2
MAP2K3
SEC22B
PDE4DIP
PRIM2
CBX3
AP3S1
FAM104B
HLA−C
DUSP22
PCMTD1
ERAP2
CD24
HLA−B
HLA−A
SLC35E2B
HSPA1A
RNF138
RBMX
Samples
B
Percent
0−25
25−50
50−75
75−100
% SNVs filtered out across samples
VPKE
DGV
SCHAIN
Filter

Figure S13

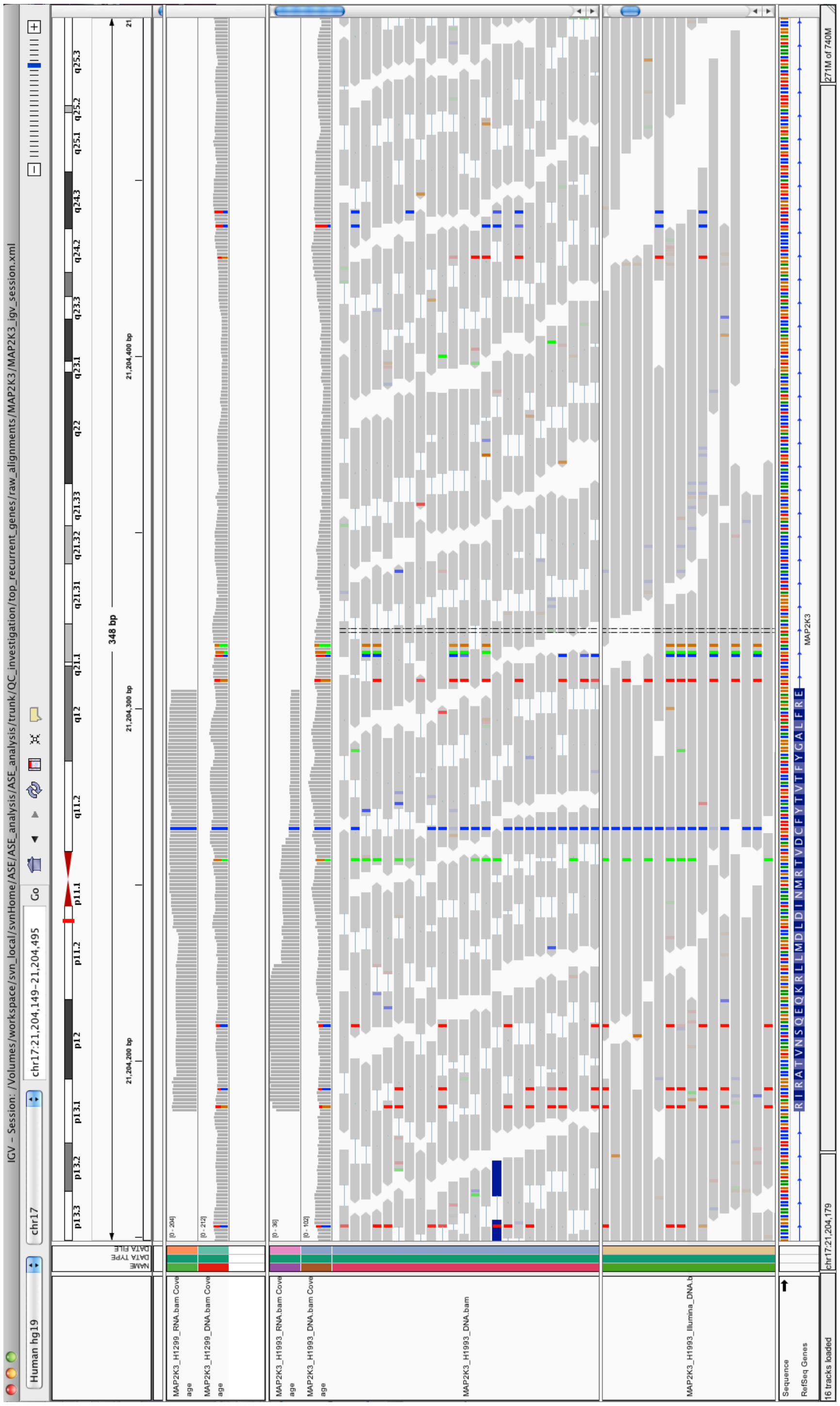

Figure S14

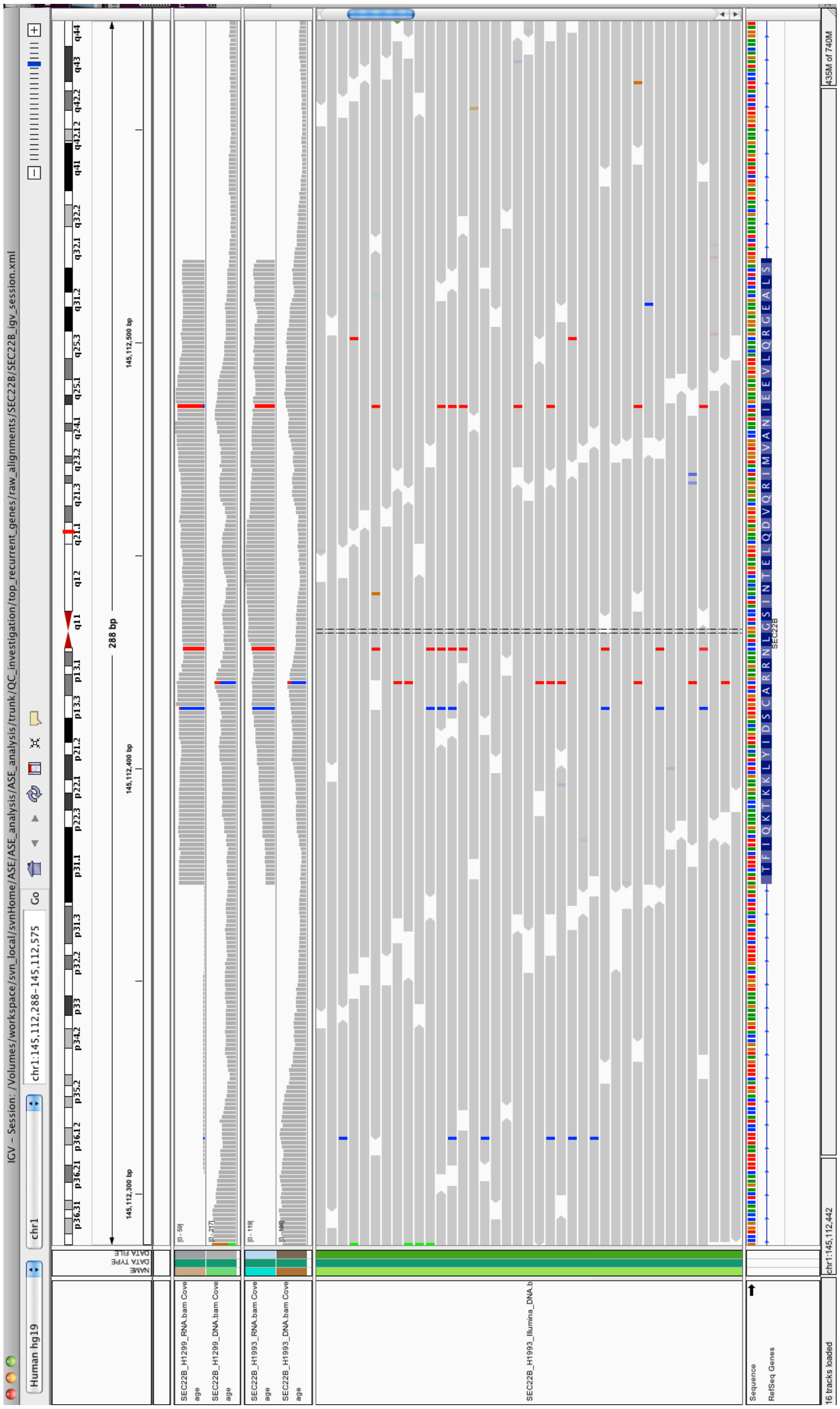

IGV - Session: /Volumes/workspace/svn_local/svnHome/ASE/ASE_analysis/ASE_analysis/trunk/QC_investigation/top_recurrent_genes/raw_alignments/SEC22B/SEC22B_igv_session.xml
Human hg19
chr1
chr1:145,112,288-145,112,575
Go
288 bp
145,112,300 bp
145,112,400 bp
145,112,500 bp
SEC22B_H1299_RNA.bam Coverage
SEC22B_H1299_DNA.bam Coverage
SEC22B_H1993_RNA.bam Coverage
SEC22B_H1993_DNA.bam Coverage
SEC22B_H1993_Illumina_DNA.b
Sequence
RefSeq Genes
SEC22B
16 tracks loaded
chr1:145,112,442
435M of 740M

Figure S15

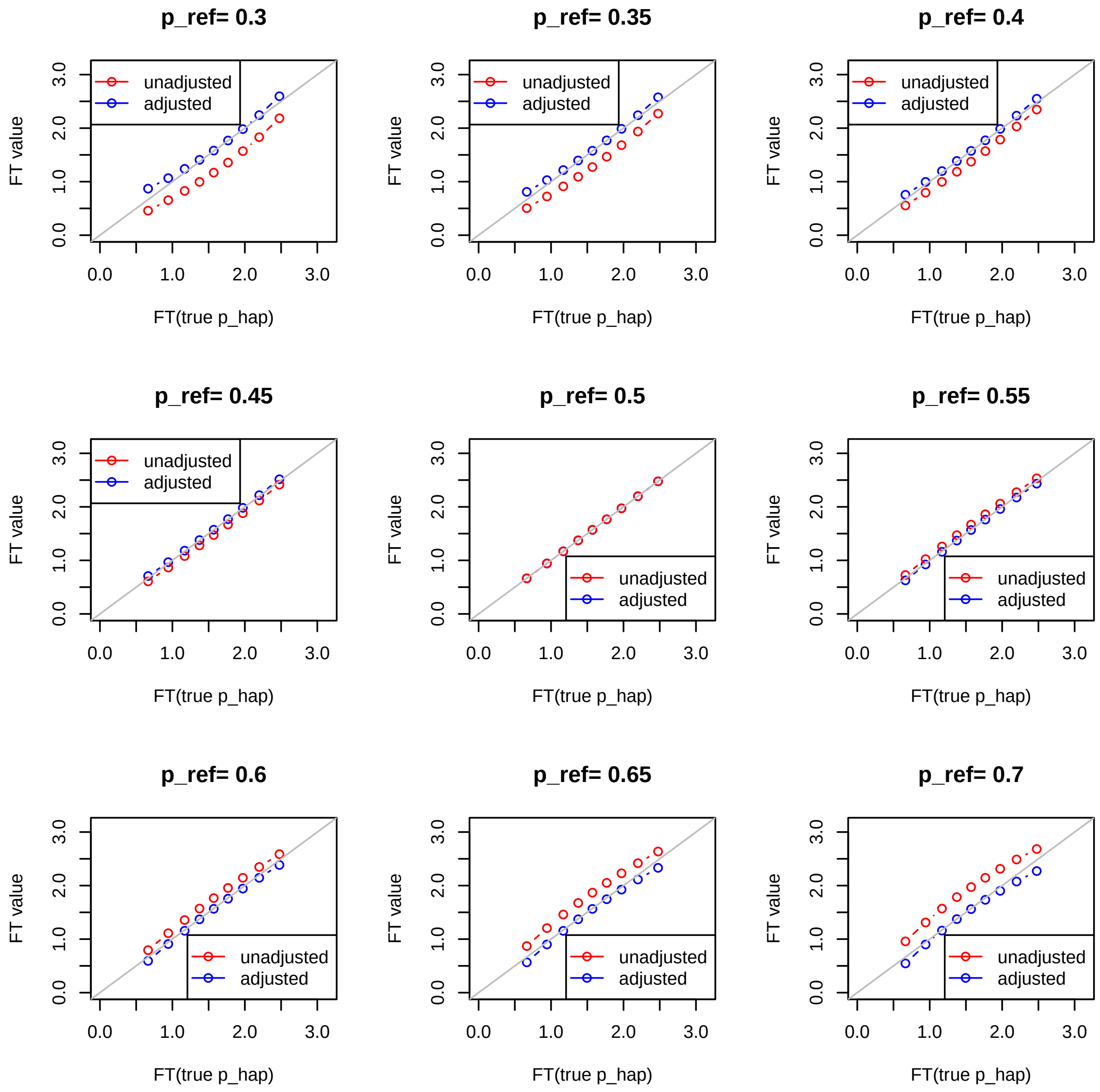
p_ref= 0.3
p_ref= 0.35
p_ref= 0.4
p_ref= 0.45
p_ref= 0.5
p_ref= 0.55
p_ref= 0.6
p_ref= 0.65
p_ref= 0.7
unadjusted
adjusted
FT value
FT(true p_hap)
0.0
1.0
2.0
3.0

Figure S16

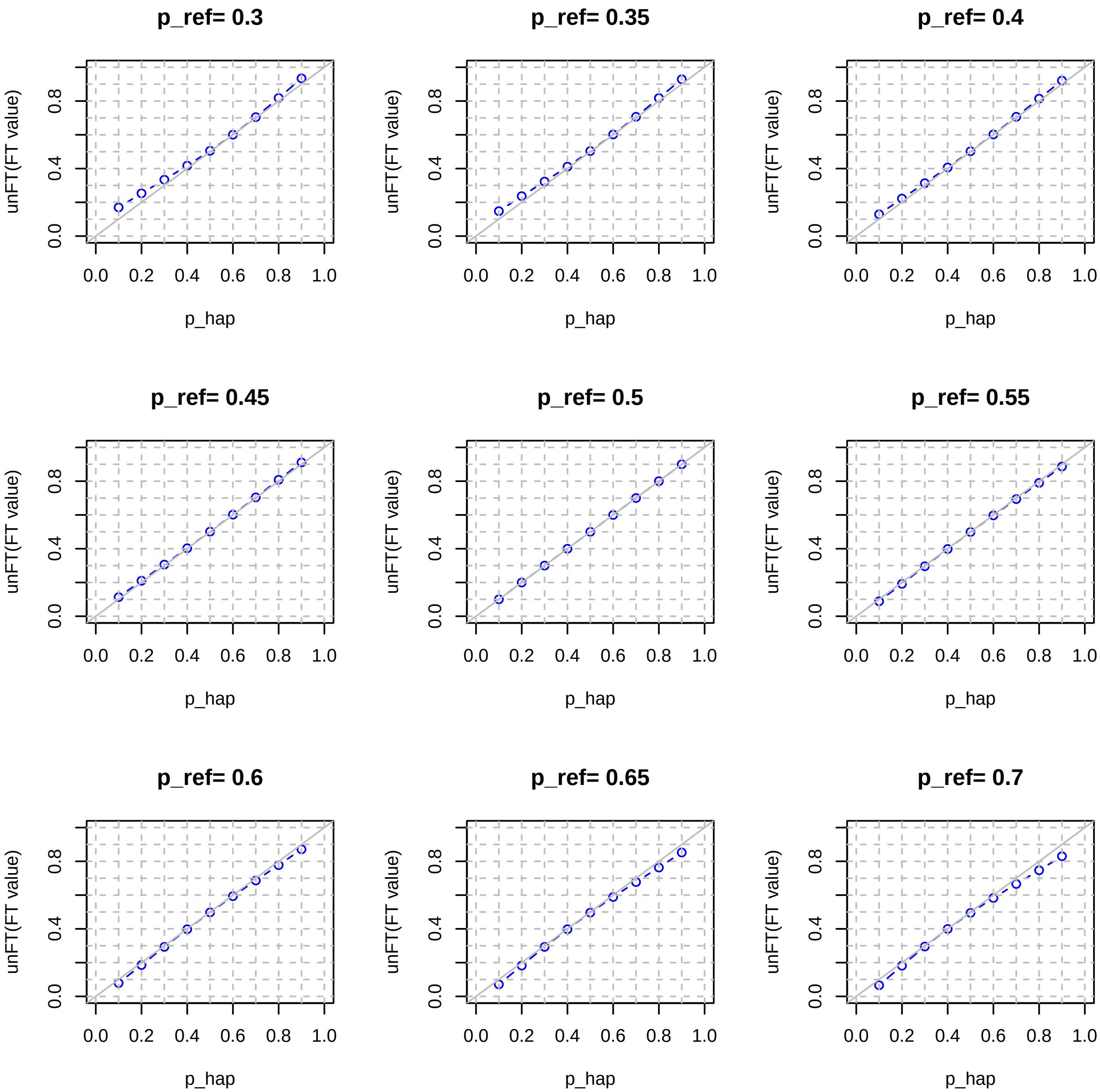
p_ref= 0.3
p_ref= 0.35
p_ref= 0.4
p_ref= 0.45
p_ref= 0.5
p_ref= 0.55
p_ref= 0.6
p_ref= 0.65
p_ref= 0.7
unFT(FT value)
p_hap
0.0
0.4
0.8
0.0
0.2
0.4
0.6
0.8
1.0

Figure S17

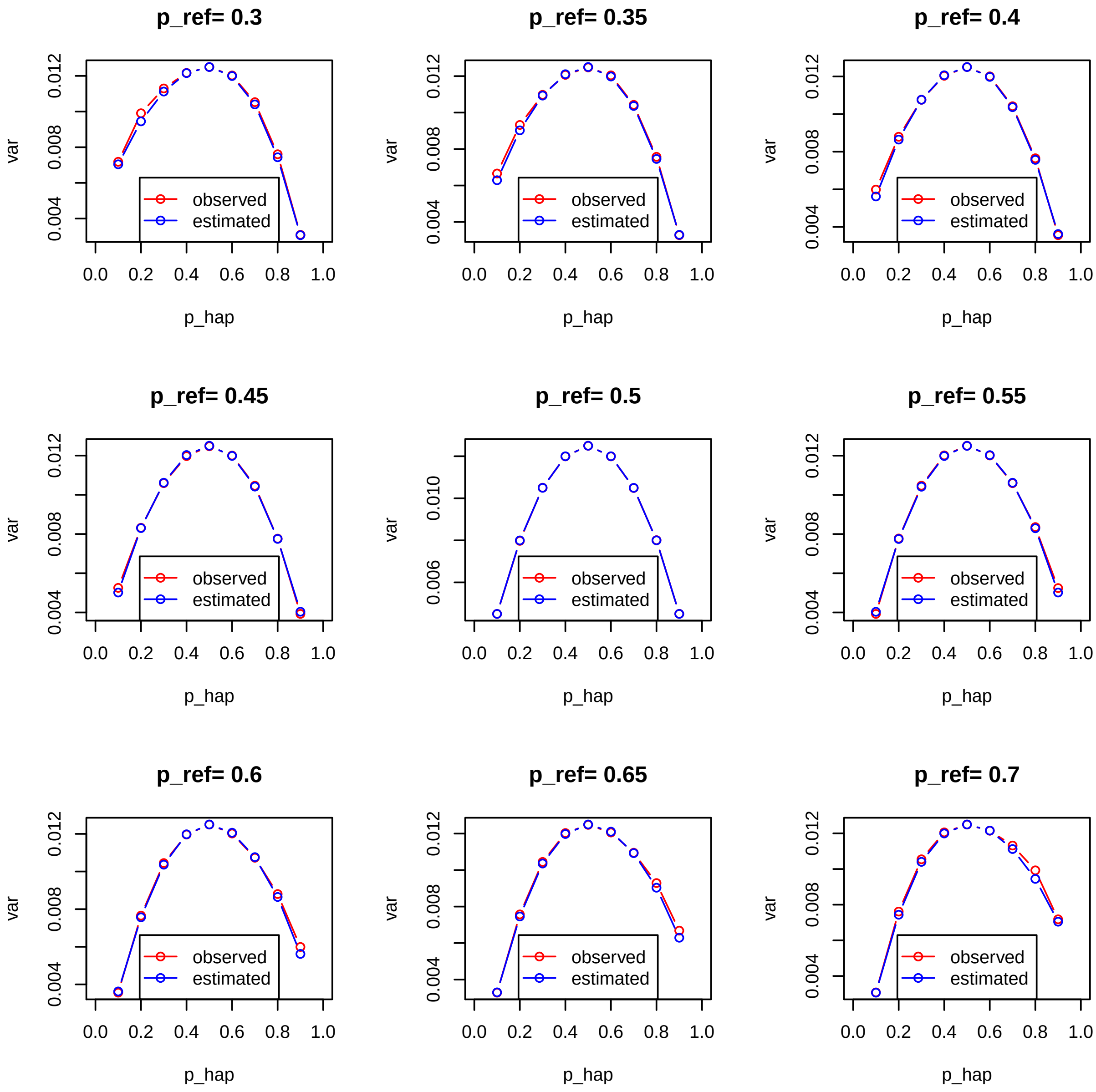
p_ref= 0.3
p_ref= 0.35
p_ref= 0.4
p_ref= 0.45
p_ref= 0.5
p_ref= 0.55
p_ref= 0.6
p_ref= 0.65
p_ref= 0.7
var
p_hap
observed
estimated
0.004
0.006
0.008
0.010
0.012
0.0
0.2
0.4
0.6
0.8
1.0

Figure S18

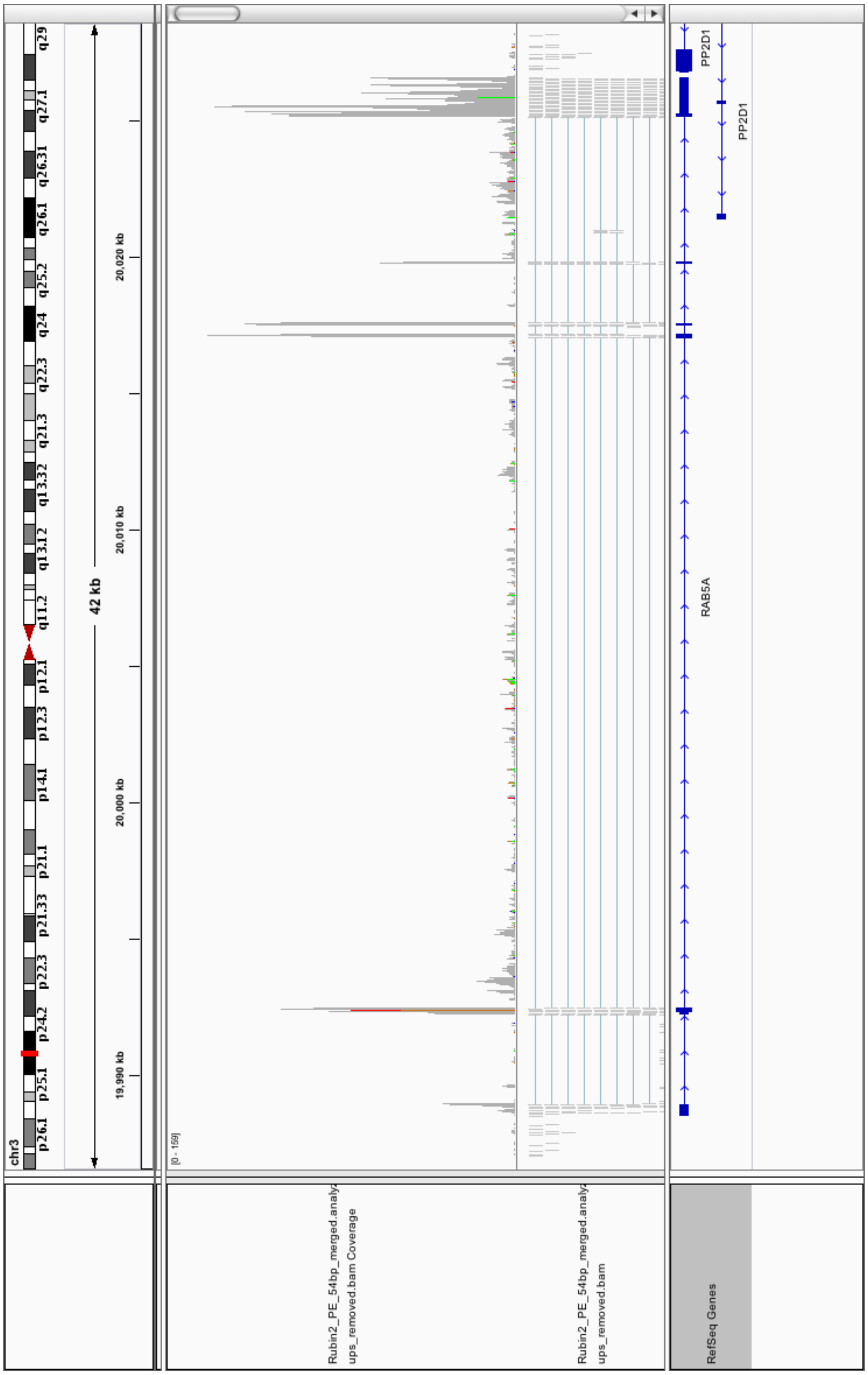
chr3
42 kb
19,990 kb
20,000 kb
20,010 kb
20,020 kb
RAB5A
PP2D1
PP2D1
RefSeq Genes

Figure S19

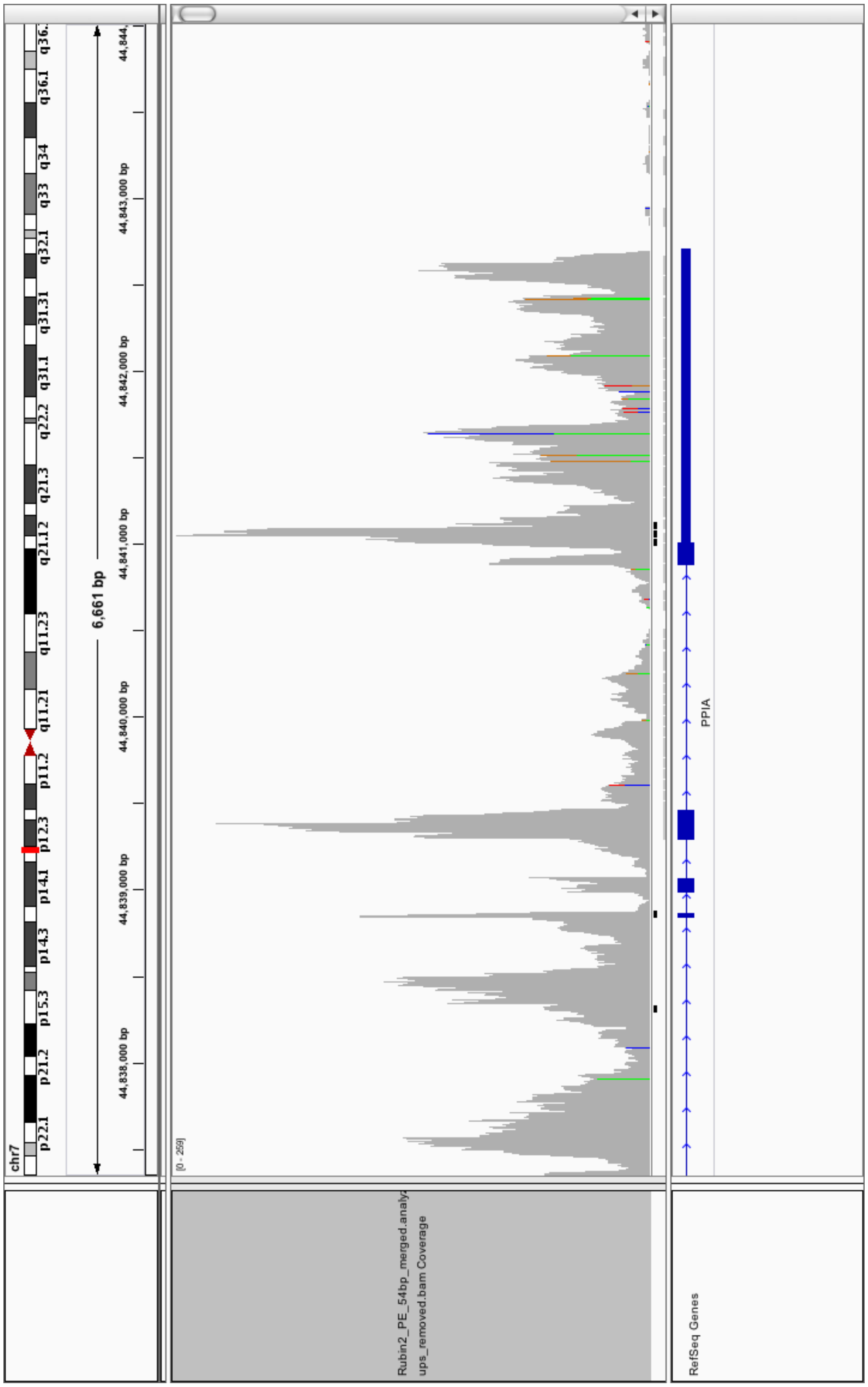
chr7
6,661 bp
44,838,000 bp
44,839,000 bp
44,840,000 bp
44,841,000 bp
44,842,000 bp
44,843,000 bp
[0 - 259]
Rubin2_PE_54bp_merged.analy
ups_removed.bam Coverage
PPIA
RefSeq Genes

Figure S20

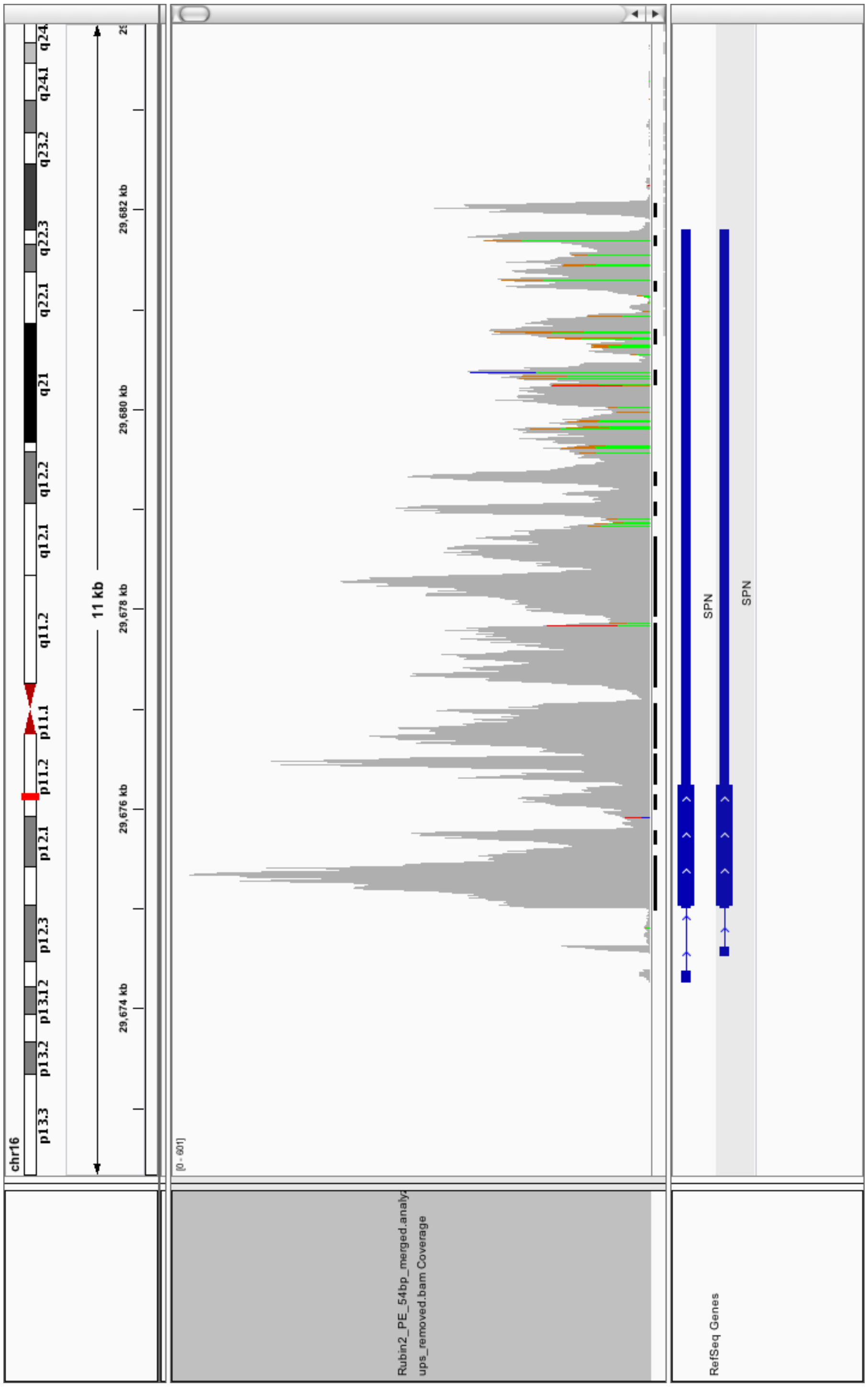
chr16
p13.3
p13.2
p13.12
p12.3
p12.1
p11.2
p11.1
q11.2
q12.1
q12.2
q21
q22.1
q22.3
q23.2
q24.1
11 kb
29,674 kb
29,676 kb
29,678 kb
29,680 kb
29,682 kb
[0 - 601]
Rubin2_PE_54bp_merged.analy
ups_removed.bam Coverage
SPN
SPN
RefSeq Genes

Figure S21

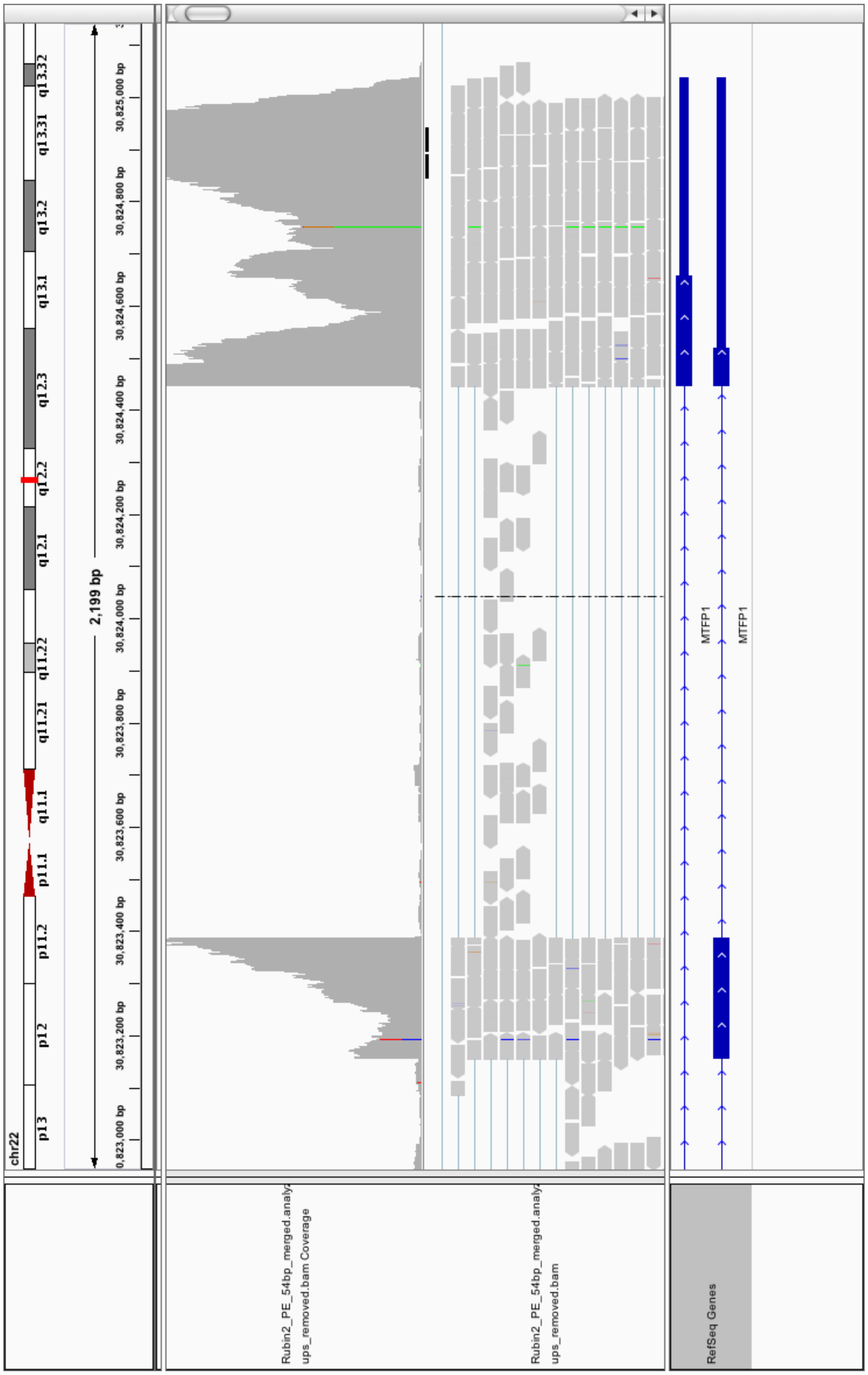

Figure S22

Supplementary Tables

See Additional File 2 for Tables S1 and S2.

Table S1. Summary of samples used in the study.

Table S2. All functional somatic mutations in canonical RefSeq transcripts. Uniprot_swissprot_accession: accession identifier used to look up affected protein domains in UniProt database. AA_change: amino acid effect, based on Variant Effect Predictor. Mutant_allele_haplotype: whether the mutant allele falls onto a major or minor haplotype, as called by MBASED. CN: copy number status of the gene in the sample, one of 'gain', 'loss', 'neutral', 'unknown'. AI: allelic imbalance status of the gene in the sample, one of 'yes', 'no', 'unknown'. ASE_major_allele_frequency: MBASED-derived estimate of MAF. ASE_adjusted_pvalue: Benjamini-Hochberg adjusted (within a sample) MBASED-derived ASE p-value. ASE_adjusted_het_pvalue: Benjamini-Hochberg adjusted (within a sample) MBASED-derived inter-loci variability p-value. Effect: Variant Effect Predictor output. SIFT: SIFT output. PolyPhen: PolyPhen output. Affected_domain_VEP: affected domains, according to Variant Effect Predictor. Affected_domain_uniprot: affected domains, according to UniProt annotation.

Supplementary Data Sets

See Additional Files 3, 4 and 5 for Data Sets S1, S2, and S3, respectively

Data Set S1. ASE results for all genes tested in analysis of NA12878 individual. Gene: gene name. Chr: chromosome. SNV_location: locations of SNVs used to test ASE in the gene (multiple SNVs are separated by ';'). SNV_haplotypeClusterIDs: haplotype clusters assigned to SNVs (SNVs with the same haplotype cluster ID have reads in common, are merged into a single locus and have their reads combined into a single locus count). Hap1Alleles: SNV alleles of first haplotype (either ref or alt). Hap1Allele_counts: read counts supporting first haplotype at each SNV (total counts for each haplotype cluster are shown). Hap2Allele_counts: read counts supporting second haplotype at each SNV. phased_MBASED_Hap1AlleleStatus: whether the haplotype1 is major or minor (the status is the same for alleles within same haplotype within a gene). phased_MBASED_MAF: MBASED-derived estimate of MAF when true haplotypes are supplied. phased_MBASED_pvalue: MBASED-derived ASE p-value when true haplotypes are supplied. phased_ adj_MBASED_pvalue: Benjamini-Hochberg adjusted (within a sample) MBASED-derived ASE p-value when true haplotypes are supplied. phased_MBASED_ILV_pvalue: MBASED-derived inter-loci variability p-value (NA for single-locus genes) when true haplotypes are supplied. phased_adj_MBASED_ILV_pvalue: Benjamini-Hochberg adjusted (within a sample) MBASED-derived inter-loci variability p-value when true haplotypes are suppled. phased_MBASED_ASE: whether gene passes all ASE cutoffs when true haplotypes are supplied. unphased_MBASED_Hap1AlleleStatus: whether the haplotype1 allele is assigned to major or minor haplotype by pseudo-phasing when the true haplotypes are not supplied to MBASED. unphased_MBASED_MAF: MBASED-derived estimate of MAF when the true haplotypes are not supplied to MBASED. unphased_MBASED_pvalue: MBASED-derived ASE p-value when the true haplotypes are not supplied to MBASED. unphased_ adj_MBASED_pvalue:

Benjamini-Hochberg adjusted (within a sample) MBASED-derived ASE p-value when the true haplotypes are not supplied to MBASED. unphased_MBASED_ILV_pvalue: MBASED-derived inter-loci variability p-value (NA for single-locus genes) when the true haplotypes are not supplied to MBASED. unphased_adj_MBASED_ILV_pvalue: Benjamini-Hochberg adjusted (within a sample) MBASED-derived inter-loci variability p-value when the true haplotypes are not supplied to MBASED. unphased_MBASED_ASE: whether gene passes all ASE cutoffs when the true haplotypes are not supplied to MBASED. Skelly_Hap1AlleleStatus: whether haplotype1 is found to be major or minor by the method of Skelly et. al (based on posterior median of haplotype 1 frequency). Skelly_MAF: major allele frequency based on the method of Skelly et al. (posterior median of the frequency of the major haplotype). Skelly_posterior_probability: posterior probability of ASE based on the method of Skelly et al. (derived according to method tutorial). Skelly_ASE: whether gene passes ASE cutoffs (imposed by us) based on output of the method of Skelly et al.

Data Set S2. ASE results for all genes tested in 1-sample MBASED analysis. SNV_location: locations of SNVs used to test ASE in the gene (multiple SNVs are separated by ';'). SNV_haplotypeClusterIDs: haplotype clusters assigned to SNVs (SNVs with the same haplotype cluster ID have reads in common, are merged into a single locus and have their reads combined into a single locus count). Hap1Alleles: SNV alleles assigned to first haplotype (either ref or alt, the assignment is arbitrary but is consistent for SNVs in the same haplotype cluster). Hap1Allele_counts: read counts supporting first haplotype at each SNV (total counts for each haplotype cluster are shown). Hap2Allele_counts: read counts supporting second haplotype at each SNV. MBASED_MAF: MBASED-derived estimate of MAF. MBASED_pvalue: MBASED-derived ASE p-value. Adj_MBASED_pvalue: Benjamini-Hochberg adjusted (within a sample) MBASED-derived ASE p-value. MBASED_ILV_pvalue: MBASED-derived inter-loci variability p-value (NA for single-locus genes). Adj_MBASED_ILV_pvalue: Benjamini-Hochberg adjusted (within a sample) MBASED-derived inter-loci variability p-value. ASE: whether gene passes all ASE cutoffs in the sample. CN: copy number status of the gene in the sample, one of 'gain', 'loss', 'neutral', 'unknown' (CN status is 'unknown' for all genes in normal samples). AI: allelic imbalance status of the gene in the sample, one of 'yes', 'no', 'unknown' (AI status is 'unknown' for all genes in normal samples). Imprinting_status: imprinting status according to geneimprint.com, if known.

Data Set S3. ASE results for all genes tested in 2-sample MBASED analysis. SNV_location: locations of SNVs used to test ASE in the gene (multiple SNVs are separated by ';'). SNV_haplotypeClusterIDs: haplotype clusters assigned to SNVs (SNVs with the same haplotype cluster ID have reads in common, are merged into a single locus and have their reads combined into a single locus count). Hap1Alleles: SNV alleles assigned to first haplotype (either ref or alt, the assignment is arbitrary but is consistent for SNVs in the same haplotype cluster). Sample1_hap1Allele_counts, sample2_hap1Allele_counts: read counts supporting first haplotype at each SNV, in sample1 and sample2, respectively (total counts for each haplotype cluster are shown). Sample1_hap2Allele_counts, sample2_hap2Allele_counts: read counts supporting second haplotype at each SNV. MBASED_MAF_difference: MBASED-derived estimate of MAF difference (sample1 MAF - sample2 MAF). MBASED_pvalue: MBASED-derived ASE p-value. Adj_MBASED_pvalue: Benjamini-Hochberg adjusted (within a sample) MBASED-derived ASE p-value. MBASED_ILV_pvalue: MBASED-derived inter-loci variability p-value (NA for single-locus genes). Adj_MBASED_ILV_pvalue: Benjamini-Hochberg adjusted (within a sample) MBASED-derived inter-loci variability p-value. ASE: whether gene passes all ASE cutoffs in the sample. CN: copy number status of the gene in sample1, one of 'gain', 'loss',

‘neutral’, ‘unknown’ (CN status is ‘unknown’ for all genes in normal samples). AI: allelic imbalance status of the gene in sample1, one of ‘yes’, ‘no’, ‘unknown’ (AI status is ‘unknown’ for all genes in normal samples). Imprinting_status: imprinting status according to geneimprint.com, if known.
